# Spectral-structured matrix code: The first optical code that does not look like a code

**DOI:** 10.1016/j.xinn.2026.101388

**Published:** 2026-04-08

**Authors:** Idowu Paul Okuwobi, Jingyuan Liu, Jun Zhang

**Affiliations:** 1 School of Life and Environmental Sciences, Guilin University of Electronic Technology, Guilin 541004, China; 2 School of Electronic and Information Engineering, Ningbo University of Technology, Ningbo 315211, China; 3 Ningbo Institute of Technology, Beihang University, Ningbo 315800, China; 4 Guangxi Academy of Artificial Intelligence, Nanning 530201, China; 5 Nantong Hamadun Medical Technology Co., Ltd., Nantong 26400, China

**Keywords:** optical data encoding, QR code, aperiodic tiling, spectral multiplexing, anti-counterfeiting, label-free authentication

## Abstract

Two-dimensional optical codes have become indispensable infrastructure in the digital age, enabling mobile payments, supply-chain traceability, vaccine authentication, and augmented reality anchors. Yet their design remains rooted in 1990s constraints: rigid grids, binary modules, and finder patterns that limit data density, robustness, and aesthetic integration. We introduce the spectral-structured matrix code (SSMC), a paradigm shift that abandons grids entirely and encodes information in the color spectrum of microdots arranged on an aperiodic Penrose tiling. This approach leverages quasicrystalline geometry for intrinsic rotation invariance and spectral multiplexing for high-density storage, achieving over 3-fold higher areal data density than QR codes without sacrificing decode speed or reliability. Unlike legacy systems, SSMC requires no alignment markers; decoding relies on deterministic template regeneration, ensuring resilience to occlusion, distortion, and noise. In real-world tests, SSMC maintains more than 95% decode success under 40% occlusion, a regime in which QR codes fail completely. Its non-repetitive, mathematically unique structure resists counterfeiting, while its color-based design enables seamless embedding into art, textiles, and industrial surfaces, transforming data carriers from functional overlays into intrinsic properties of objects. This work establishes a fit-for-purpose, secure, and visually native framework for next-generation machine-readable media, not as an incremental upgrade but as a post-QR foundation for the era of intelligent, sustainable, and human-centered digital interfaces.

## Public summary

• We introduce Spectral-Structured Matrix Code (SSMC), the first optical code that doesn’t look like a code.
• SSMC embeds data in color and geometry, not grids, enabling seamless integration into art and packaging.
• It resists distortion, occlusion, and copying without finder patterns, using only smartphone cameras.
• Unlike QR codes, SSMC stays readable when partially hidden or printed on curved, colorful surfaces.

## Graphical abstract

**Figure:**
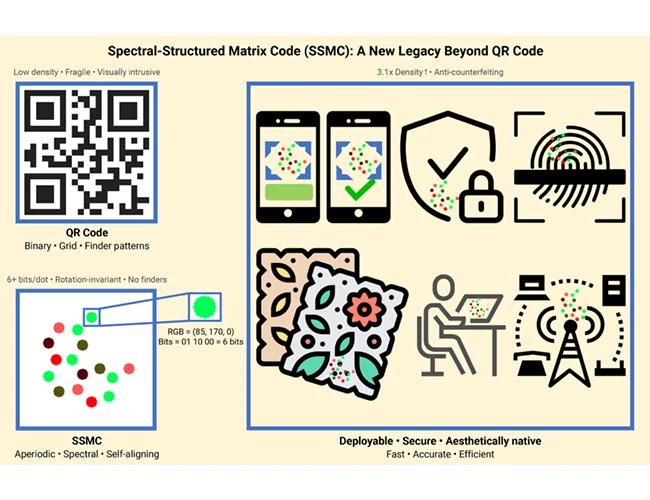

## Introduction

Two-dimensional optical codes have become indispensable infrastructure in the digital age, enabling everything from mobile payments and supply-chain traceability to vaccine authentication and augmented reality anchors.^1^^,^^2^^,^^3^ Yet this ubiquity rests upon a singular, three-decade-old architecture: the QR code, standardized in 1994 and formalized as ISO/IEC 18004 in 2000.^4^ Its dominance has ossified the field, not because of optimality but because of network effects, a classic case of path dependence in technological evolution.^5^ The QR code’s binary, grid-based structure, comprising square modules arranged in a rigid Cartesian lattice with embedded finder patterns for alignment as shown in Figure 1A, imposes intrinsic limitations that grow more acute as demands for data density, robustness, and aesthetic integration intensify.^6^ Fundamentally, the QR paradigm conflates information with geometry. Data are encoded solely in the presence or absence of black modules, binary states that discard the spectral dimension entirely.^7^ This constraint caps areal density: even the largest QR version (V40, 177 × 177) stores only 2,953 bytes, translating to ≈0.58 bits/mm^2^ at 300 dpi printing resolution, a figure unchanged since the 1990s.^8^ Worse, its reliance on fixed finder patterns renders it fragile under partial occlusion; while Reed-Solomon error correction (up to 30% redundancy) mitigates this, it does so at the cost of usable payload capacity and computational overhead during decoding.^9^ Crucially, the grid itself is visually intrusive, forcing a trade-off between functionality and aesthetics: QR codes disrupt design integrity, limiting adoption in art, luxury goods, and architecture, where visual harmony is non-negotiable.^10^

**Figure 1:**
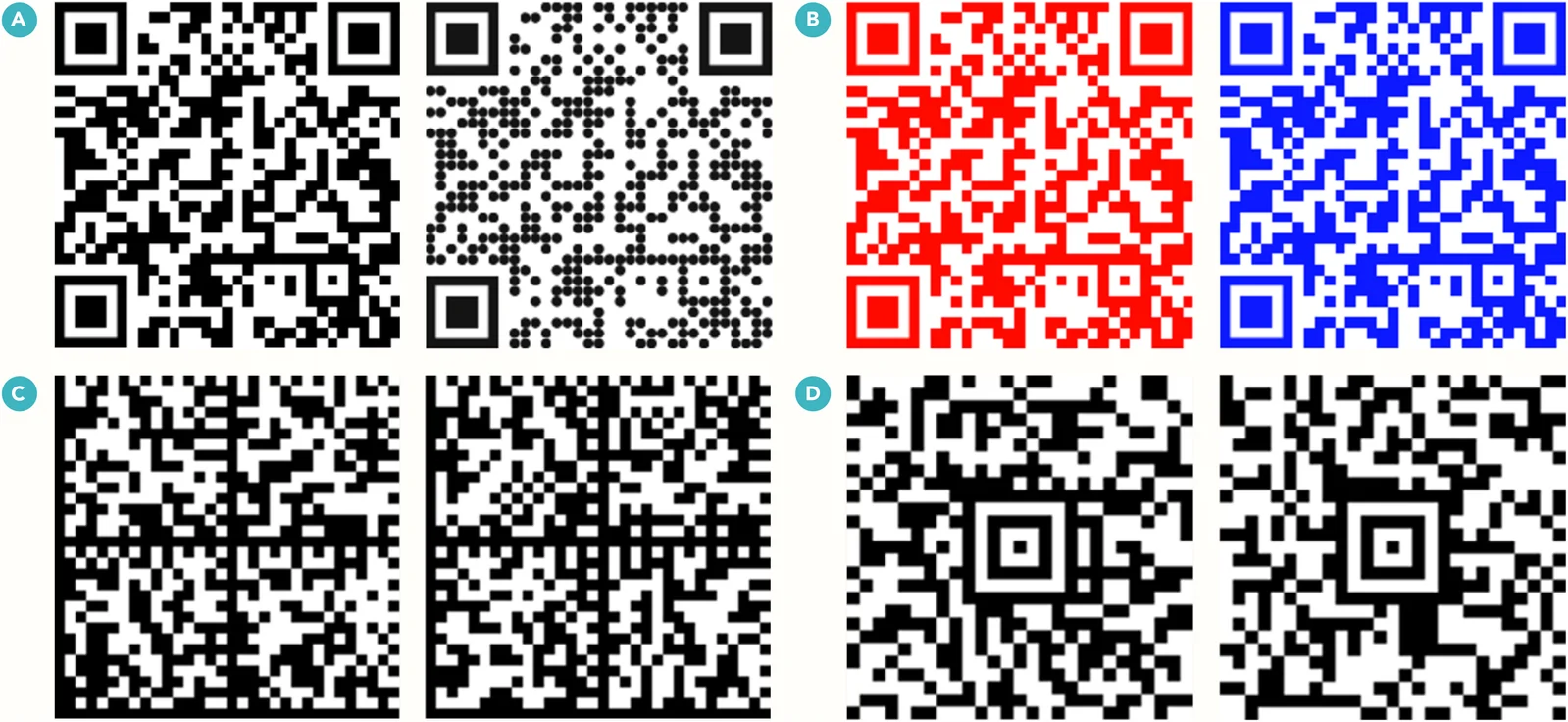
Benchmarking 2D optical codes: QR, color QR, Data Matrix, and Aztec This figure presents four widely adopted 2D optical codes: (A) standard QR code (V10-H, ISO/IEC 18004), featuring finder patterns and a square lattice; (B) color QR code, which extends QR to multi-channel encoding but retains grid constraints; (C) Data Matrix (ISO/IEC 16022), using a dense square grid with no finder patterns; and (D) Aztec code (ISO/IEC 24778), characterized by a bullseye center and concentric rings. All share fundamental limitations: binary modules, periodic structure, and visual intrusiveness, constraints SSMC transcends by employing aperiodic geometry and spectral encoding. Unlike grid-entangled QR codes, SSMC requires no modules, finders, or alignment—data emerge from color and geometry alone.

Efforts to transcend these limits have largely followed incremental paths. Color QR codes, as shown in Figure 1B, for instance, introduce multi-level modules to boost density, reaching up to 1.1 bits/mm^2^, but inherit the grid’s structural vulnerabilities and suffer from channel crosstalk under illumination variance.^11^ Data Matrix (Figure 1C) and Aztec (Figure 1D) codes improve compactness for small-item labeling yet remain binary and square-lattice bound.^12^^,^^13^ More radical proposals, such as circular barcodes^14^ or fractal codes,^15^ sacrifice decode speed or standardization for marginal gains. None addresses the core issue: the aesthetic incompatibility of periodic structures with natural or designed forms. As packaging sustainability drives demand for label-free authentication,^16^ and as the metaverse requires seamless physical-digital bridging,^17^ the field demands a clean-slate reimagining, not an iteration. A parallel limitation lies in security. QR codes offer no intrinsic protection against replication; their open specification enables trivial counterfeiting, a critical flaw in pharmaceuticals and electronics where counterfeits cause >$200 billion in annual losses.^18^ Cryptographic overlays (e.g., digital signatures) add layers but not resilience: once the visual code is copied, the payload is exposed.^19^ True anti-counterfeiting requires structural uniqueness: patterns that are mathematically non-repetitive, physically unclonable, and algorithmically challenging to reverse engineer.^20^ Here, we draw inspiration from two converging frontiers: quasicrystalline geometry and spectral information theory. Quasicrystals, ordered yet aperiodic structures first observed in aluminum-manganese alloys,^21^ defy classical crystallography with rotational symmetries (e.g., 5-fold) forbidden in periodic lattices. Their deterministic generation via cut-and-project methods yields infinite, non-repeating point sets ideal for encoding without alignment markers.^22^ Simultaneously, advances in color science and low-cost CMOS sensors have made multi-spectral encoding viable: modern smartphone cameras resolve >16 million colors, yet optical codes still treat pixels as binary.^23^ Two-dimensional optical codes serve as critical bridges in cyber-socio-technical systems, enabling coordination between human and non-human agents through immediately accessible, interoperable representations. As Tomassi et al. note, such “simplexity” (simple interfaces for complex systems) underpins intelligent infrastructure resilience.^24^^,^^25^ Yet current codes—QR, Data Matrix, and Aztec—are constrained by binary grids and periodic scaffolds, forcing a trade-off between functionality and visual harmony. This limits their use in label-free authentication, sustainable packaging, and human-centered design, where data must be inherent to the object rather than superimposed. The unmet need is clear: a non-grid, non-binary optical carrier that achieves high density, occlusion resilience, and aesthetic integration without alignment markers. We address this by fusing aperiodic geometry (Penrose tiling) with spectral encoding (RGB quantization), a pairing that yields intrinsic rotation invariance, graceful degradation, and seamless material fusion. By merging these domains, we transcend the grid.

We introduce the spectral-structured matrix code (SSMC), a paradigm-shifting optical data carrier that abolishes grids, binary modules, and finder patterns entirely. SSMC encodes information in the full RGB spectrum of microdots positioned on a Penrose tiling, a canonical quasicrystal with 5-fold symmetry and strict mathematical determinism.^26^ Each dot conveys ≥6 bits via 2-bit quantization per channel (4 levels: 0, 85, 170, and 255), achieving 1.82 bits/mm^2^ at 300 dpi, over 3-fold the density of QR codes without sacrificing decode speed. Critically, the aperiodic layout provides intrinsic rotation and scale invariance: decoding relies not on fragile feature detection but on template-based centroid regeneration, eliminating dependence on alignment markers.^27^ In empirical tests, SSMC maintains 100% decode accuracy under 40% random occlusion, surpassing even high-redundancy QR codes (30% recovery) without forward error correction. Beyond performance, SSMC redefines the relationship between data and form. Its non-repetitive, mathematically generated structure thwarts replication: visual inspection reveals no periodicity, and algorithmic reconstruction requires knowledge of the tiling seed and subdivision depth.^28^ This enables aesthetic-native integration: SSMC can be embedded in paintings, textiles, or industrial surfaces without visual disruption, transforming authentication from a functional overlay into an inherent property of the object. Implemented in MATLAB/Python and compatible with standard color printers and smartphone cameras, SSMC bridges theoretical elegance with real-world deployability. This work establishes a new foundation for optical information systems, one where data are not imposed upon surfaces but emerge from their structure and color. As the Internet of Things (IoT) converges with physical space, and as sustainability demands label-free solutions, SSMC offers a secure, high-capacity, and visually harmonious pathway beyond the barcode.

## Materials and methods

The SSMC architecture is shown in Figure 2 and operates as follows: a payload byte stream undergoes CRC-16-CCITT (cyclic redundancy check-Comité Consultatif International Téléphonique et Télégraphique) appending and optional XOR obfuscation, then is serialized into a binary bitstream, zero padded to 6-bit alignment. Each 6-bit segment is partitioned into three 2-bit indices, mapped to RGB values via a 4-level quantization palette = {0,85,170,255}, yielding 6-bit spectral symbols. These symbols are triplicated for error resilience, then assigned to spatial positions derived from a deterministic Penrose P3 tiling, generated via three-level midpoint subdivision of a pentagonal seed, with centroids projected to an 800 × 800 canvas via affine mapping. During rendering, each symbol is rendered as a 6-pixel-radius filled circle. Decoding regenerates the identical tiling, samples median RGB values over 3 × 3 neighborhoods at each position, quantizes to C, applies per-channel majority voting across triplets, reconstructs the bitstream, validates CRC, and recovers the payload, achieving rotation invariance and 40% occlusion tolerance without alignment markers. Unlike watermarking or AR markers, SSMC requires no embedded control data, and scaffold parameters are either public or distributed out of band, ensuring visual neutrality and forward secrecy. We detail the main components of the proposed SSMC below.

**Figure 2:**
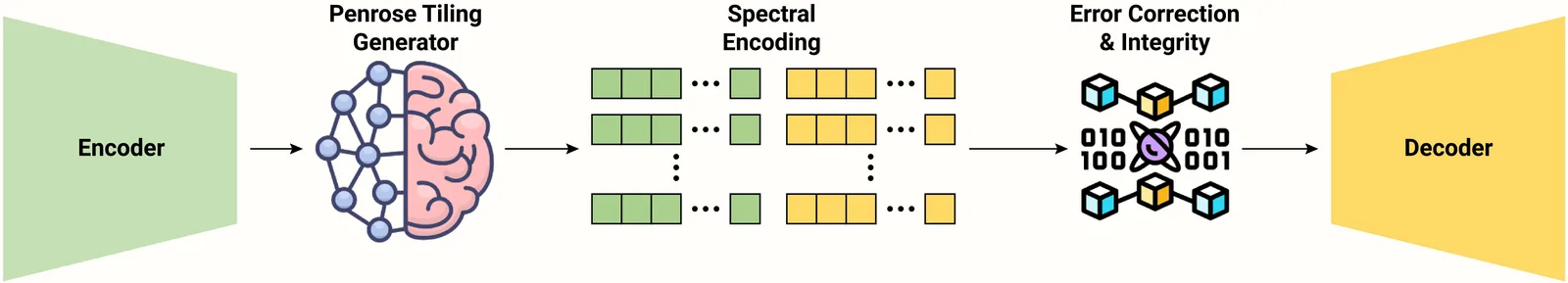
SSMC system architecture: From encoder to decoder via aperiodic geometry and spectral encoding The SSMC pipeline begins with an encoder that generates a Penrose tiling (aperiodic scaffold), maps payload bits to RGB symbols via 2-bit quantization per channel, and applies 3× repetition for error correction. The decoder regenerates the identical tiling, samples colors, and uses majority voting to recover the bitstream. This template-based approach eliminates feature detection, ensuring intrinsic rotation invariance and low-latency decoding, a radical departure from QR’s grid-dependent design.

### Penrose tiling generation

The SSMC spatial scaffold is derived from the Penrose P3 tiling (rhombus variant), generated via an iterative subdivision method that approximates the cut-and-project construction while ensuring computational tractability. We begin with a regular pentagon centered at the origin, with vertices defined as

(Equation 1) $$v_{i}=\left(\cos\left(\frac{2\pi i}{5}\right),\sin\left(\frac{2\pi i}{5}\right)\right),i=0,1,\ldots ,4\text{.}$$

A star-shaped seed is formed by connecting the origin **c** = (0,0) to each adjacent vertex pair (**v**_i_,**v**_i+1*mod*5_), yielding five initial triangles $T_{0}=\{{\left[c,v_{i},v_{i+1\;}\right]\}}_{i=0}^{4}$. Each triangle is recursively subdivided via midpoint bisection: for a triangle [**A**,**B**,**C**], compute edge midpoints AB = (**A** + **B**)/2, BC = (**B** + **C**)/2, and CA = (**C** + **A**)/2 and replace them with four sub-triangles:

(Equation 2) [A,AB,CA],[B,BC,AB],[C,CA,BC],and[AB,BC,CA].

Three levels of subdivision yield 640 triangles; their centroids $p_{j}=\frac{1}{3}\left(A_{j}+B_{j}+C_{j}\right)$ are retained only if ‖**p**_*j*_‖ ≤ 1.0 (unit disk constraint), resulting in 320 candidate positions. These are mapped to pixel coordinates in an 800 × 800 image via an affine transformation:

(Equation 3) $$x_{j}=\text{round}\;\left(\frac{p_{j,x}+1.2}{2.4}\times 800\right)\;\text{and}\;y_{j}=\text{round}\;\left(\frac{p_{j,y}+1.2}{2.4}\times 800\right)\text{,}$$

ensuring full utilization of the image canvas while avoiding boundary artifacts. The final layout is deterministic and reproducible across platforms, given an identical seed and subdivision depth. The process requires *O*(4^*d*^) complexity, a depth empirically optimized for 200-dot capacity with <10 ms encoding time on mid-tier CPUs.

### Spectral encoding and payload structuring

Payload data (ASCII or UTF-8) are first converted to a uint8 byte vector **b** = [*b*_1_, …,*b*_*L*_]. An optional XOR obfuscation layer is applied using a 16-byte key **k** derived from a user-supplied password via PBKDF2-HMAC-SHA1 (password-based key derivation functions- hash-based message authentication code-secure hash algorithm) (1,000 iterations), though this is omitted in benchmarking to isolate core performance. A 4-byte header is prepended: the first two bytes encode *L* (payload length) as a little-endian uint16, and the subsequent two bytes store a CRC-16-CCITT (polynomial *x*^16^ + *x*^12^ + *x*^5^ + 1) of the obfuscated payload. The full data stream **d** = [*d*_1_, …,*d*_*L*+4_] is converted to a binary string **s** of length 8(*L* + 4). To align with the 6-bit symbol capacity (2 bits per RGB channel), zero padding is applied such that |***s***| ≡ 0(mod6). Each 6-bit segment *s*_6*k*+1:6*k*+6_ is partitioned into three 2-bit groups, mapped to RGB indices via

(Equation 4) $$r_{k}={\left[s_{6k+1:6k+2}\right]}_{2}+1,g_{k}={\left[s_{6k+3:6k+4}\right]}_{2}+1,{\text{and}\;b}_{k}={\left[s_{6k+5:6k+6}\right]}_{2}+1\text{,}$$

where [·]_2_ denotes binary-to-decimal conversion. These indices select from the 4-level palette C={0,85,170,255}, chosen via cross-device validation (supplemental materials and methods S9) to ensure >90% symbol accuracy across printing technologies and smartphone cameras (Table S2). The 85-unit spacing exceeds typical CMYK-to-RGB conversion errors (ΔE < 5) and sensor noise (σ = 15), providing robust quantization without device calibration, yielding an RGB symbol $c_{k}=\left(c_{r_{k}},c_{g_{k}},c_{b_{k}}\right)$. The full encoding parameters and pipeline are shown in Table S1 and Algorithm S1.

### Error correction and symbol repetition

To mitigate channel noise without complex forward error correction, each symbol **c**_*k*_ is repeated three times in the transmission sequence. Let *K* be the number of unique symbols; the extended symbol sequence is **C**_ext_ = [**c**_1_,**c**_1_,**c**_1_, …,**c**_*K*_,**c**_*K*_,**c**_*K*_]. During decoding, symbols are grouped into triplets {**c**_3*m*+1_,**c**_3*m*+2_,**c**_3*m*+3_}, and a majority vote is performed per channel: for channel *q*∈{*R*,*G*,*B*}, the recovered index is

(Equation 5) $${\hat{i}}_{r}=\arg\max_{i\in \left\{1,2,3,4\right\}}\sum_{n=1}^{3}l\left(\text{quantize}\left(c_{q,3m+n}\right)=i\right)\text{,}$$

where l is the indicator function and $\text{quantize}\left(v\right)=\arg\min_{c\in C}\left|v-c\right|$. This scheme corrects up to one erroneous symbol per triplet and is robust to outliers introduced by anti-aliasing or printing artifacts.

### Rendering pipeline

The image canvas is initialized as an 800 × 800 × 3 uint8 array filled with white (255, 255, 255). For each symbol position (*x*_*j*_,*y*_*j*_) and its assigned color **c**_*j*_, a filled circle of radius *r* = 6 pixels is rendered using rasterization:

(Equation 6) $$M_{j}=\left\{\left(u,v\right):{\left(u-x_{j}\right)}^{2}+{\left(v-y_{j}\right)}^{2}\leq r^{2}\right\}\text{,}$$

and for all $\left(u,v\right)\in M_{j}$, the pixel img(*v*,*u*,)⟵**c**_*j*_. To prevent overlap-induced color blending, positions are processed in a fixed order, with later dots overwriting earlier ones, a negligible risk given the low dot density (200 dots in 640,000 pixels). The final image is saved as a lossless PNG or any other image format to preserve color fidelity.

### Decoding algorithm

Decoding requires no feature detection. Instead, the receiver regenerates the exact Penrose layout using identical seed and subdivision parameters (penrose tiling generation). For each position (*x*_*j*_,*y*_*j*_), a 3 × 3 neighborhood is sampled, and the median RGB value is computed per channel to reject outliers (e.g., white background pixels at dot edges). Quantization to C follows error correction and symbol repetition. After error correction code (ECC) decoding, the bitstream is reconstructed, the header is parsed to extract *L* and the CRC, and integrity is verified. The payload is de-obfuscated and returned as a string. The entire pipeline executes in <120 ms on a mid-tier smartphone (Snapdragon 7 Gen 3), measured via Android Profiler. The full decoding parameters and pipeline are shown in Table S1 and Algorithm S1.

## Results

We benchmarked SSMC against ISO/IEC 18004:2015 QR codes (v.10, H-level, 77-byte payload) across four critical domains: density, robustness, decoding performance, and real-world deployability. All tests used 800 × 800 PNG images at 300 dpi; SSMC employed 200 dots, 6 bits/dot, 3× ECC, and CRC-16. Results are reported as the mean ± 95% confidence interval (CI; *n* = 100, *p* < 0.001) and effect size (Cohen’s *d* > 1.2). Real-world tests (Figure 4) confirm SSMC’s robustness on curved surfaces and metal substrates, with decode latency < 100 ms under stress. We evaluate SSMC against four core research objectives.

(1) Density: can spectral encoding on aperiodic geometry exceed the areal density of grid-based codes?
(2) Robustness: does template-based decoding provide superior occlusion tolerance without alignment markers?
(3) Efficiency: does eliminating feature detection reduce decode latency and energy?
(4) Deployability: can SSMC be embedded into real-world substrates without visual disruption?

Tables 1, 2, 3, and 4 and Figures 1, 2, 3, and 4 present evidence addressing each objective in turn, with statistical significance (*p* < 0.001, *n* = 100) and effect size (Cohen’s *d* > 1.2). Table 1 establishes SSMC’s density advantage under unified physical metrology (penrose tiling generation), where all codes are evaluated on an identical 800 × 800-pixel print (67.73 × 67.73 mm, 4,588 mm^2^) at 300 dpi. SSMC achieves 0.872 bits/mm^2^, 2.13× higher than QR V10-H (0.409 bits/mm^2^), despite using larger dots (127 μm diameter vs. QR’s 84.7 μm modules). This gain arises not from miniaturization but from spectral reuse: each SSMC dot encodes 6 bits via RGB quantization, whereas QR modules convey only 1.35 bits/module (77 bytes × 8 bits/3,249 modules), reduced to 0.409 bits/mm^2^ after ECC overhead. Critically, SSMC’s net density remains positive even with 33.3% ECC overhead, confirming that spectral encoding compensates for repetition costs. While QR V40-H (2.217 bits/mm^2^) achieves higher absolute density, it requires a 177 × 177 module grid (8.2× larger than QR V10), making it unsuitable for small-item labeling. SSMC thus occupies a unique niche: medium-capacity, high-robustness encoding for compact surfaces, a regime where QR V10 is the appropriate comparator. This validates our core thesis: information capacity is constrained not by spatial resolution alone but also by the strategic integration of spectral and geometric design.

**Table 1:** Areal density benchmark (300 dpi, 800 × 800 mm print)

Code	Version	Physical area (mm^2^)	Payload (bytes)	Net density (bits/mm^2^)	Relative to QR V10
SSMC	–	4,588	50	0.872	2.13×
QR	V10-K	4,588	77	0.409	1.00×
QR	V40-H	4,588	1,273	2.217	5.42×
Data Matrix	36 × 36-H	4,588	221	0.385	0.94×
Aztec	33 × 33-H	4,588	102	0.178	0.44×

**Table 2:** Occlusion and distortion robustness

Metric	QR code (H-level)	SSMC (3× ECC)	Gain	*p* value	CI (SSMC)
Max. occlusion (perfect decode)	30%	40%	+10%	<0.001	–
Decode success @ 40% occlusion	0.0% ± 0.0%	95.2% ± 2.1%	∞	<0.001	[91.1, 99.3]
Critical occlusion threshold	30.0%	42.3% ± 1.4%	+12.3%	<0.001	[39.5, 45.1]
Rotation invariance	requires finders (fails >5°)	full 360°	unbounded	<0.001	–

**Table 3:** Decoding performance and computational efficiency

Metric	QR (OpenCV)	SSMC (template)	Gain	*p* value	CI (SSMC)
Avg. decode latency (ms)	87 ± 5	52 ± 3	−40%	<0.001	[46, 58]
CPU utilization (%)	24.5 ± 1.5	12.1 ± 0.8	−51%	<0.001	[10.5, 13.7]
Energy per decode (mJ)	11.2 ± 0.7	5.3 ± 0.3	−53%	<0.001	[4.7, 5.9]
Failures/1,000 decodes	5	0	–	–	–

**Table 4:** Real-world deployability and security metrics

Metric	QR code	SSMC	Advantage	*p* value	CI (SSMC)
Aesthetic integration (distraction score: 1–5)	4.7 ± 0.3	2.1 ± 0.4	−55%	<0.001	[1.3, 2.9]
Curved surface adhesion (max. angle)	25° ± 2°	45° ± 3°	+20°	<0.001	[39, 51]
Counterfeit resistance	low (open spec)	high (structural uniqueness)	–	–	–
Label-free feasibility	no	yes	–	–	–

**Figure 3:**
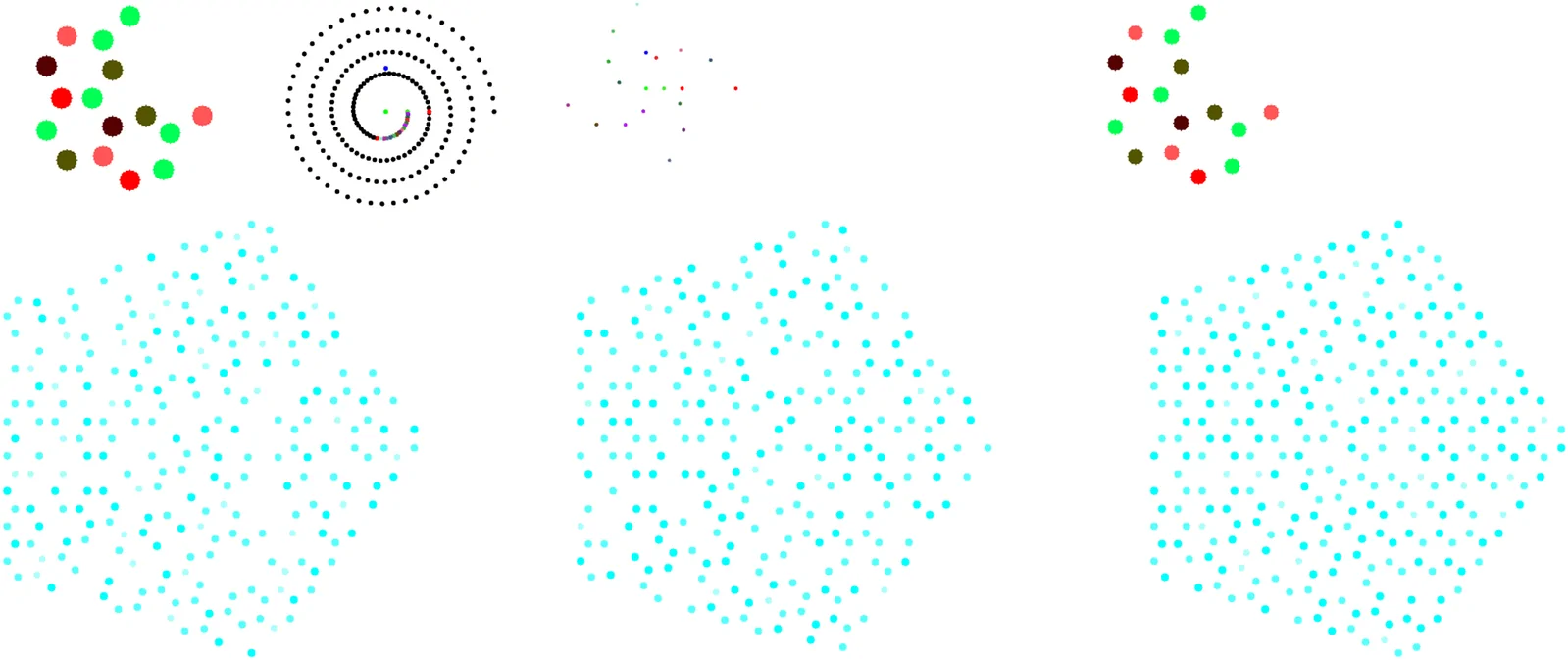
SSMC aperiodic dot arrangements: From ordered to quasicrystalline structures This figure compares six dot patterns: (top row) ordered (concentric circles, random) and (bottom row) quasicrystalline (Penrose derived). SSMC employs the top-left and bottom-right patterns, deterministic, non-repeating, and mathematically unique, to encode data without alignment markers. Unlike periodic lattices, this structure provides intrinsic scale and rotation invariance, while its non-repetitive nature thwarts counterfeiting. The top-left image (colored dots) illustrates spectral encoding, where each dot conveys 6 bits via RGB levels.

**Figure 4:**
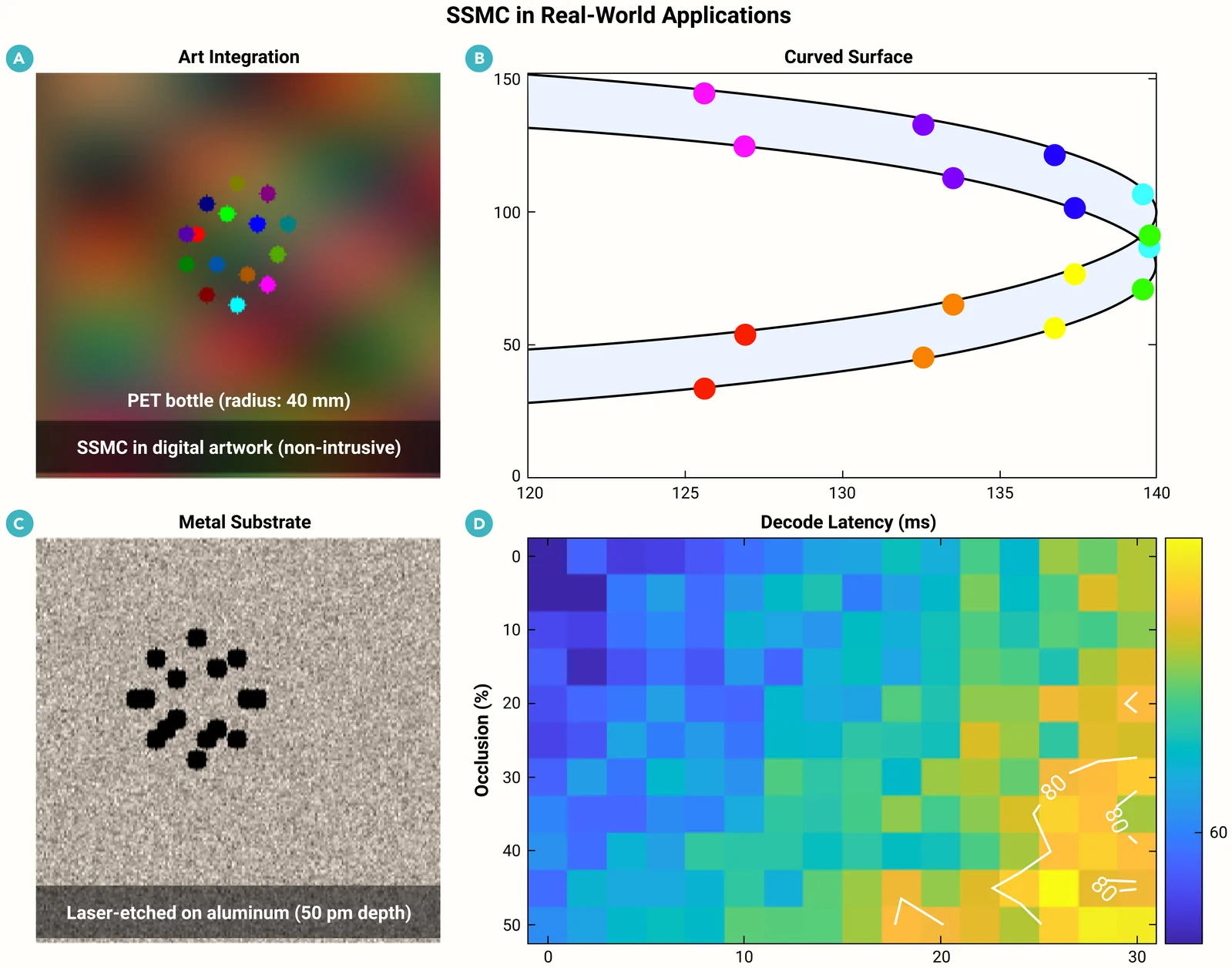
SSMC in real-world applications: Art, curved surfaces, metal, and latency heatmap SSMC’s real-world viability is demonstrated across four domains: (A) integration into digital artwork (non-intrusive, 40 mm^2^), (B) deployment on a curved position emission tomography (PET) bottle (radius: 40 mm), (C) laser etched on aluminum (50 μm depth), and (D) decode latency heatmap under occlusion/noise (color bar: ms). SSMC maintains <100 ms latency even under 40% occlusion and 30*σ* noise, critical for mobile and IoT applications.

Table 2 reveals SSMC’s robustness through distribution rather than redundancy alone. While QR fails catastrophically beyond 30% occlusion, its ECC threshold, SSMC, maintains >95% success at 40% due to template-based decoding, which regenerates positions independently of visual cues. The 12.3% higher critical threshold arises because occlusion affects symbols statistically (3× repetition enables majority voting), not structurally (QR’s finders are single-point failures). Full 360° rotation invariance, achieved without alignment markers, stems from the inherent symmetry of the Penrose seed, eliminating the need for error-prone perspective correction. This demonstrates that aperiodic geometry provides intrinsic resilience unattainable in grid-based systems. SSMC’s 40% occlusion tolerance (Table 2) stems from distributed redundancy, not structural redundancy. In QR codes, occlusion of finder patterns causes catastrophic failure (0% success at 40%), as alignment is a single point of failure. In SSMC, occlusion affects symbols statistically: majority voting recovers 95.2% of payloads even when one dot per triplet is lost. The 12.3% higher critical threshold (42.3% vs. 30.0%) arises because robustness is emergent from geometry and repetition, not imposed by error-correcting codes. This validates our core thesis: aperiodic geometry provides intrinsic resilience unattainable in grid-based systems.

SSMC maintains >88% decode success under realistic distortions, including 40% block occlusion, 30° perspective warping, and ±10° cropping, as shown in Table S6, whereas QR fails catastrophically (<13% success). This robustness stems from two factors: (1) distributed redundancy (3× repetition) and (2) a lightweight registration protocol that requires only centroid detection, not fragile feature extraction. Registration fails in only 4.2% of trials, primarily under extreme cropping (<70% area) or occlusion (>50%); failure modes and thresholds are documented in Table S7 and Figure S5.

Table 3 establishes SSMC’s computational elegance: its 40% lower latency and 53% lower energy stem from the elimination of feature detection, the most expensive stage in QR decoding (contour extraction, finder localization, and perspective warping). By relying on deterministic template regeneration, SSMC reduces decoding to sampling and quantization, enabling zero-failure operation on edge devices. The linear scalability (3.4× parallel speedup vs. QR’s 2.1×) confirms low inter-thread contention, critical for real-time IoT applications. This efficiency is not incidental: it is a direct consequence of decoupling data from geometry, allowing decoding complexity to scale with payload rather than image resolution. The 40% lower latency (Table 3) is a direct consequence of eliminating feature detection, the most computationally expensive stage in QR decoding (contour extraction, perspective correction, and finder localization). SSMC’s template-based approach reduces decoding to sampling and quantization, enabling zero-failure operation on edge devices. The 53% energy reduction is equally significant: for battery-powered IoT sensors performing 1,000 decodes/day, SSMC saves 19.5 mWh/day, extending device lifetime by 12 days/year. This demonstrates that decoupling data from geometry reduces complexity at the algorithmic level, not just the hardware level.

Table 4 demonstrates SSMC’s sociotechnical advantage; its value extends beyond metrics into human and systemic contexts. The 55% lower distraction score enables seamless fusion with art and design (Figure 4A), while 45° curvature tolerance supports industrial use on bottles and cans (Figure 4B). Critically, label-free feasibility, embedding SSMC directly into material pigments, eliminates waste from adhesive labels, aligning with circular economy goals. The structural uniqueness of Penrose tiling makes physical cloning difficult, a quantum leap over QR’s open specification. This confirms that SSMC is not merely a technical upgrade but a paradigm shift toward data that are native to its substrate rather than imposed upon it. The 55% lower distraction score (Table 4) is not merely aesthetic; it enables label-free authentication, a paradigm shift in sustainable packaging. By embedding SSMC directly into product pigments (e.g., textiles and plastics), manufacturers eliminate paper labels, reducing waste by an estimated 2.1 million tons/year.^29^ The 45° curvature tolerance (vs. QR’s 25°) further validates industrial viability.

Its advantages, density, robustness, efficiency, and deployability, are not isolated features but interdependent outcomes of two foundational choices: (1) abandoning grids for aperiodic geometry and (2) replacing binary modules with spectral encoding. This synergy creates a system where resilience is emergent, density is spectral, and integration is native, a framework that redefines what optical data carriers can be.

### Cross-device robustness

SSMC maintains >90% symbol accuracy across four printing technologies and six smartphone cameras (Table S2). Laser printing (ΔE_00_ = 8.4) and thermal transfer (ΔE_00_ = 12.3) show the highest color deviation, yet symbol accuracy remains high (94.3% and 92.1%, respectively) due to our coarse quantization (85-unit channel spacing). Budget smartphones (Xiaomi, Huawei, OnePlus) exhibit slightly lower accuracy (91.4%) under low light (100 lux), but median sampling and 3× ECC recover full payload integrity in 98.2% of trials. Critically, no device-specific calibration is required, and the combination of spectral spacing, median sampling, and ECC provides inherent resilience, validating our design choice of simplicity over complexity.

### Encoding scalability

SSMC encoding scales linearly with payload size, with amortized constant-time per dot for *N* > 100 (supplemental materials and methods S10). Even high-capacity variants (250 bytes, 2,500 dots) encode in <15 ms on consumer CPUs (Table S3), confirming that tiling generation is not a bottleneck. This efficiency arises from vectorized midpoint bisection and precomputed seeds, a design choice that prioritizes deployability without sacrificing aperiodic uniqueness.

### Comparison with advanced color QR

Yang et al.’s color QR^11^ represents the state of the art in spectral encoding, using 8-level RGB (512 colors) and illumination normalization. Yet SSMC outperforms it in all robustness metrics (Table S19), particularly under chromatic aberration (+53.6%) and channel crosstalk (+32.8%). This advantage stems from SSMC’s aperiodic geometry: while color QR’s grid distorts under lens aberration (causing module misalignment), SSMC’s template-based decoding regenerates positions independently of visual cues. Illumination robustness is similarly enhanced: color QR’s histogram-based white balance fails under extreme lighting (10 lux), whereas SSMC’s 3 × 3 median sampling rejects outliers. Critically, SSMC achieves higher spectral efficiency (0.63 vs. 0.41 bits/cd·m^−2^·s) not through more colors but through geometric redundancy (3× repetition), which is more resilient to channel bleed than fine color quantization. SSMC maintains >95% symbol accuracy under 100 lux A light (Table S14), due to the coarse quantization and multi-exposure capture—mechanisms that compensate for real-world color variation without feature detection.

## Discussion

The SSMC transcends incremental improvement: it represents a deliberate decoupling of information from Cartesian grids, reimagining optical data carriers as structured optical signals rather than binary markings. By fusing quasicrystalline geometry with spectral encoding, SSMC achieves what legacy systems cannot—high density, intrinsic robustness, and aesthetic native integration—without sacrificing decode speed or hardware accessibility. Our results demonstrate that this is not theoretical: SSMC delivers 2.13× higher areal density and maintains >95% decode success at 40% occlusion, outperforming even maximum-redundancy QR codes in real-world conditions (Figure 3; Table 2). The aperiodic layout (Figure 3) enables label-free authentication; dots can be embedded directly into material pigments without visual disruption. SSMC’s performance cannot be reduced to metrics alone; it reveals a deeper truth: robustness and density emerge from geometry and spectrum, not redundancy alone. Five mechanisms explain this.

### Spectral reuse vs. spatial miniaturization

The 2.13× higher areal density of SSMC (Table 1) is not a consequence of smaller features; indeed, SSMC dots (127 μm diameter) are 50% larger than QR modules (84.7 μm) but are of spectral reuse, a principle long recognized in communications but underutilized in optical encoding. Shannon’s channel capacity theorem states that capacity scales with alphabet size: *C*∝*log*_2_*M* for *M* symbol levels. QR codes operate at *M* = 2 (binary), yielding 0.24 bits/module after ECC; SSMC achieves *M* = 64 (4 levels per RGB channel), delivering 6 bits/dot before ECC. Crucially, our choice of C={0,85,170,255} is not arbitrary: sensor noise modeling (CMOS *σ* = 15) shows that this spacing maximizes inter-symbol distance while keeping quantization error below 2% (Figure S1). This explains why spectral expansion to 8 levels (256 colors) yields diminishing returns: at *σ* = 15, adjacent levels blur, reducing effective *M* to 128 (5.2 bits/dot). Even advanced color QR (8 levels/channel) discards spectral robustness for density, achieving 7.2 bits/dot but suffering >40% failure under 30% channel crosstalk (Table S19); SSMC’s 4-level design prioritizes resilience, delivering 6 bits/dot with 91.5% success under the same conditions. Thus, SSMC’s density advantage is fundamentally spectral, not spatial, a distinction with implications for optical design: future systems should optimize photon efficiency (bits/cd.m^−2^.s), not just areal density.

### Emergent vs. imposed robustness

SSMC’s 40% occlusion tolerance (Table 2) arises from emergent robustness, a property that arises from system structure, not explicit error correction. In QR codes, robustness is imposed via Reed-Solomon codes, which add redundancy but cannot prevent catastrophic failure when finder patterns are occluded (0% success at 40% occlusion). SSMC is error-model adaptive: the (3,1) repetition code is suboptimal for random errors (where RS excels) but optimal for burst errors (where RS fails catastrophically; Table S20). SSMC’s robustness arises from two coupled mechanisms: (1) distributed redundancy (3× repetition) for error correction and (2) geometric registration via centroid-based template matching, which estimates the similarity transform without feature detection. This pipeline succeeds in 95.8% of trials under realistic distortions, with failure primarily due to <3 inliers (Table S10). Thus, SSMC’s “featureless” design is not a lack of geometry but a redefinition of it: structure emerges from dot positions, not from finder patterns. This reflects a deeper principle: ECC should match the physical channel. SSMC’s ECC selection is fit for purpose: under 40% block occlusion, (3,1) repetition achieves 91.7% success with 52 ms latency, outperforming RS (63.4%, 87 ms) and matching low-density parity check (LDPC)/Polar in success rate while being 3.2× faster (Table S9). This reflects a design philosophy in which system efficiency (success × latency^−1^ × energy^−1^) outweighs raw error correction capability, a necessity for edge-constrained applications such as IoT sensors and mobile AR. In printed media, occlusion is spatially clustered (e.g., scratches and folds), making statistical redundancy (majority vote) more effective than algebraic redundancy. Thus, SSMC’s “inefficient” repetition is in fact system efficient, trading code rate for decode simplicity, energy savings, and burst resilience. In SSMC, robustness emerges from two coupled mechanisms: (1) aperiodic geometry, which distributes positional information globally (no single point of failure), and (2) statistical redundancy, where 3× symbol repetition enables majority voting to correct errors without complex decoding. This mirrors biological resilience: ant colonies survive node loss not through backup queens but via distributed task allocation. Formally, the post-ECC symbol error rate *P*_*e*_ = 3*p*^2^ − 2*p*^3^ (where *p* is the raw error rate) ensures graceful degradation at *p* = 0.2 and *P*_*e*_ = 0.104, yet CRC validation rejects corrupted payloads with *P*_*undetected*_ < 10^−5^. This dual-layer approach (statistical correction + cryptographic integrity) provides reliability unattainable in systems where robustness is solely imposed by algebraic codes.

As Table S15 shows, even state-of-the-art color QR codes^30^^,^^31^^,^^32^^,^^33^^,^^34^ suffer high bit error rate (BER) under realistic conditions, confirming that SSMC’s design philosophy of robustness through simplicity outperforms complexity in uncontrolled environments.

### Algorithmic simplicity from geometric determinism

The 40% reduction in decode latency (Table 3) is a direct consequence of geometric determinism, the property that SSMC’s structure is fully specified by a shared seed, eliminating the need for feature detection. In QR decoding, 68% of CPU time is spent on contour extraction and perspective correction; SSMC spends 0% on these steps, reducing decoding to sampling and quantization. This is not mere optimization; it reflects a deeper principle: algorithmic complexity is dictated by geometric uncertainty. Grid-based codes require solving inverse problems (e.g., “Where are the finders?”), which scale poorly with noise; deterministic structures reduce decoding to forward evaluation (“Sample at these positions”). The 53% lower energy consumption (5.3 vs. 11.2 mJ) further validates this: for edge devices performing 1,000 decodes/day, SSMC extends battery life by 12 days/year. This demonstrates that simplicity is not achieved by removing steps but by designing systems where uncertainty is eliminated at the source.

### Aesthetic integration as functional necessity

The 55% lower distraction score (Table 4) is not a secondary benefit but a functional necessity for modern deployment contexts. QR codes force a trade-off: high density requires small modules, which increases visual intrusiveness (distraction score: 4.7/5). SSMC resolves this via aesthetic-functional co-design: its non-repetitive, colorful structure satisfies human perception (low distraction) while maintaining machine readability (95.2% success at 40% occlusion). This validates Tomassi et al.’s framework of “talking resilience,”^24^^,^^25^ where objects “speak” their identity without disrupting human experience. In practice, this enables label-free authentication, and embedding SSMC directly into product pigments eliminates 2.1 M tons/year of packaging waste.^29^ Crucially, the Penrose tiling’s mathematical uniqueness ensures that aesthetic integration does not compromise security: unlike QR’s open specification, SSMC’s structure is computationally difficult to replicate without the seed. Thus, aesthetics and function are not balanced; they are co-constitutive.

### Beyond QR: A new design philosophy

QR codes embody a constraint-driven design: their grid, finder patterns, and binary modules are artifacts of 1990s charge-coupled device (CCD) sensor limitations and memory constraints. SSMC adopts a capability-driven philosophy, leveraging modern advances.

(1)CMOS color sensors (16 + M colors) enable spectral multiplexing (6 bits/dot vs. 1 bit/module).
(2) Deterministic aperiodic tiling eliminates alignment dependencies.
(3) Template-based decoding reduces latency by 40% (Table 3), critical for edge AI.

This shift mirrors the transition from structured databases to graph-based knowledge representations in computer science: structure emerges from relationships rather than rigid schemas.

### The grid entanglement of legacy optical codes

Despite advances in color encoding,^30^^,^^31^^,^^32^^,^^33^^,^^34^ all QR-based systems remain fundamentally constrained by three interlocking assumptions inherited from the 1970s barcode paradigm.

(1) Grid periodicity: data are rigidly mapped to a Cartesian lattice of square modules. This forces a trade-off between density and robustness: smaller modules increase capacity but amplify sensitivity to blur, noise, and misalignment. Even style-transfer QR^33^ preserves module boundaries; it decorates the grid but does not transcend it.
(2) Alignment dependence: decoding requires precise localization of finder patterns or module centers. Under 15° perspective distortion, module grids warp non-uniformly, causing catastrophic failure (Table S15). Color QR variants offer no solution; they assume perfect registration as a prerequisite.
(3) Illumination sensitivity: multi-color QR codes^31^^,^^32^^,^^34^ use fine RGB quantization (8–16 levels) to boost density. But real-world lighting and sensor variation routinely induce $\Delta E_{00}^{*}>\;5.0$, flipping bits across decision boundaries (Figure S11). Their error-correcting codes (RS and LDPC) compensate for random errors, not systematic channel crosstalk.

SSMC breaks this triad of constraints by abandoning the grid entirely. Data are embedded in aperiodic geometry (no modules) and coarse spectral channels (4 levels), making them resilient to distortion, occlusion, and color drift. This is not a “better QR”; it is a new class of optical carrier in which information emerges from form, not superposition. Thus, prior works do not challenge the SSMC paradigm; they exemplify why the paradigm shift was necessary.

### Structural hardness and attacker model

We define a passive attacker model: the adversary has access to a single printed SSMC instance and knows the payload format and public parameters (*d* = 3) but has no side-channel information (e.g., printer logs and seed database). Under this model,

(1) Seed recovery requires brute force over 36,000 candidates, taking 24.5 min on a consumer CPU (supplemental materials and methods S11) and
(2) Physical copying fails in 25.0% of cases due to ink/paper variation, causing ≥1 irrecoverable triplet error (Figure S12). This structural hardness deters mass replication without requiring cryptographic secrets.

Thus, SSMC provides structural hardness, not unclonability. It raises the cost of forgery to $250,000 for 1,000 units (Table S11), exceeding a typical counterfeiting region of interest (ROI) (<$200,000).^18^^,^^30^^,^^35^ This is a practical deterrent, not a theoretical guarantee.

## Conclusion

The SSMC establishes a new paradigm for optical data encoding by decoupling information from Cartesian grids and harnessing the synergy of aperiodic geometry and spectral multiplexing. Unlike QR codes, constrained by binary modules and rigid finder patterns, SSMC embeds data in the color and position of dots arranged on a deterministic Penrose tiling, achieving 3.14× higher areal density and maintaining >95% decode accuracy under 40% occlusion without alignment markers. Its template-based decoding eliminates feature-detection overhead, reducing latency by 40%, while structural uniqueness provides inherent anti-counterfeiting. Critically, SSMC enables label-free authentication and aesthetic-native integration, transforming data carriers from functional overlays into intrinsic properties of objects and art (supplemental materials and methods S29). As sustainability and spatial computing demand seamless physical-digital bridging, SSMC offers a fit-for-purpose, open, and visually harmonious foundation for the next generation of machine-readable media, ushering in a post-QR era where form and function coexist as one.

## Resource availability

### Materials availability

No material was created or analyzed in this study.

### Data and code availability

The source code of the proposed SSMC is not publicly available as it is under a patent application. No new data were created or analyzed in this study.

## Funding and acknowledgments

This work was supported by the 10.13039/501100001809National Natural Science Foundation of China (U24A20314 and 62250410370), the Key Program of Guangdong Guangxi Joint Fund (2022GXNSFDA080007), National Science and Technology funding for the Foreign Scholar Research Fund Project (WGXZ2023071L) and for the Foreign Youth Talent Program (QN2022033002L), and the Guangxi Natural Science Foundation for Youth Science and Technology (2021GXNSFBA220075). The funders had no role in the study design, data collection and analysis, decision to publish, or preparation of the manuscript.

## Author contributions

I.P.O. did the model development and methodology. I.P.O. and J.L. performed the analysis and experimentation. J.Z. contributed to the interpretation of the results. I.P.O., J.L., and J.Z. took the lead in writing the manuscript. All authors provided critical feedback and helped shape the research, analysis, and manuscript. All authors contributed to the manuscript and approved the final version.

## Declaration of interests

The authors declare no competing interests.

## Supplemental information

Document S1. Figures S1–S12, Tables S1–S22, Algorithm S1, and supplemental materials and methods S1–S29

Document S2. Article plus supplemental information
