# Supplementary material for: Spectral-structured matrix code: The first optical code that does not look like a code
Source: Innovation (Camb). 2026 Apr 8;7(10):101388. doi: 10.1016/j.xinn.2026.101388 (PMC13673548; doi:10.1016/j.xinn.2026.101388)
Supplement: Supplementary file 1 [file mmc1.pdf]

The Innovation, Volume *7*

Supplemental Information

Spectral-structured matrix code: The first optical code that does not look like a code

Idowu Paul Okuwobi, Jingyuan Liu, and Jun Zhang

Supplementary

S1. Experimental Data, Results, and Algorithms

S1.1 Encoding/Decoding Parameters

Table S1. Encoding/Decoding Parameters

Parameter	Value	Validation Method	Rationale
**Penrose Subdivision Depth**	3	Grid search (depth 1-5 vs. decode latency)	Depth 3yields 320 positions- optimal trade-offs between capacity (200 symbols) and decoding time (52 mS)
**RGB Quantization Levels**	[0,85,170,255]	Sensor noise modeling (CMOS $\sigma = 15$)	$\Delta = 85$ maximizes channel separation while ensuring >95% quantization accuracy under illumination variance
**ECC Scheme**	3× symbol repetition + majority vote	Bit error rate simulation ($p = 0.2$)	Corrects 1 symbol error per triplet (theoretical $P_e = 3p^2 - 2p^3$ at $p = 0.2$)
**CRC Polynomial**	CRC-16-CCITT (0x1021)	Undetected error probability analysis	$P_{undetected} < 10^{-5}$ for payloads ≤100 bytes
**Dot Radius**	6 pixels	Anti-aliasing robustness test (3–10 px)	6 px minimizes edge contamination in 3×3 median sampling (error <2%)
**Image Resolution**	800 × 800 pixels (300 dpi)	Print fidelity benchmark (150–600 dpi)	300 dpi ensures dot separation ≥2× sensor blur kernel ($\sigma = 2$ px)

S1.2 Cross-Device Symbol Accuracy and Color Deviation

Table S2. Cross-Device Symbol Accuracy and Color Deviation

Printer	Camera	Avg.ΔE^{*}_{00}	Symbol Accuracy
**Inkjet (Epson)**	All	3.2 ± 0.8	98.7 ± 1.1
**Laser (HP)**	All	8.4 ± 1.5	94.3 ± 2.3
**Dye-Sub (Canon)**	All	4.1 ± 0.9	97.2 ± 1.4
**Thermal (Zebra)**	All	12.3 ± 2.1	92.1 ± 3.1
**All Printers**	iPhone 14	7.1 ± 4.2	95.8 ± 2.8
**All Printers**	Samsung S23	6.8 ± 3.9	96.1 ± 2.6
**All Printers**	Google Pixel	7.3 ± 4.1	95.2 ± 3.0
**All Printers**	Budget (Xiaomi/Huawei/OnePlus)	9.2 ± 5.3	91.4 ± 4.2

Note: Symbol accuracy = % of dots correctly quantized to $C = \{0,85,170,255\}$. ΔE_{00} > 2.3 is perceptible; >5.0 is unacceptable for branding (ISO 12647-2). SSMC's 85-unit spacing ensures >90% accuracy even at ΔE = 12.3.*

S1.3 Encoding Time

Table S3. Encoding Time vs. Dot Count and Hardware

Dots	Payload (bytes)	Matlab (ms)	Python + Numba (ms)	GPU (ms)	Snapdragon 8 G (ms)
200	20	8.2 ± 0.3	6.1 ± 0.2	1.8 ± 0.1	9.3 ± 0.4
500	50	9.7 ± 0.4	7.9 ± 0.3	1.9 ± 0.1	10.1 ± 0.5
1,000	100	11.5 ± 0.5	9.2 ± 0.4	2.1 ± 0.1	11.8 ± 0.6
2,500	250	14.7 ± 0.6	11.2 ± 0.5	2.3 ± 0.1	14.9 ± 0.7

Note: Measurements are mean *± std* over 50 trials. GPU uses CUDA kernel for triangle subdivision. Mobile tests use Android NDK-compiled C++ encoder.

S1.4 Security Attack Success Rate

Table S4. Security Attack Success Rate

Attack Type	Conditions	Success Rate	Time (median)
**Brute-force**	0% occlusion, σ=0	100%	24 min
**Brute-force**	15% occlusion, σ=0	0%	-
**Geometric Hashing**	0% occlusion, σ=0	92.3%	8.7s
**Geometric Hashing**	15% occlusion, σ=0	0.2%	9.1s
**Deep Learning (ResNet-18)**	0% occlusion, σ=0	87.6%	12.4s
**Deep Learning (ResNet-18)**	15% occlusion, σ=0	0%	13.2s

Note: Attacks evaluated on 10,000 SSMC instances (200 dots, payload "SSMC"). Hardware: AWS EC2 c6i.4xlarge (16 vCPUs, 32 GB RAM).

S1.5 Density Benchmark

Table S5. Density Benchmark Across Optical Codes (300 dpi)

Code	Version	Modules/Dots	Payload (Bytes)	Physical Area (mm²)	Net Density (bits/mm²)
**SSMC**	–	200 dots	50	4,588	0.872
**QR**	V1-H	21×21	17	4,588	0.030
**QR**	V10-H	57×57	77	4,588	0.409
**QR**	V20-H	97×97	355	4,588	0.778
**QR**	V40-H	177×177	1,273	4,588	2.217
**Data Matrix**	12×12	10×10	6	4,588	0.010
**Data Matrix**	24×24	22×22	91	4,588	0.198
**Data Matrix**	48×48	44×44	914	4,588	1.591
**Aztec**	Compact-15	15×15	12	4,588	0.021
**Aztec**	Full-33	33×33	102	4,588	0.178

**Aztec**	Full-151	151×151	3,044	4,588	5.302

S1.6 Robustness Under Realistic Distortions

Table S6. Robustness Under Distortions

Metric	QR Code (H-level)	SSMC (3× ECC)
**Block Occlusion (10×10 px blocks, 40% total)**	12.3% ± 2.1%	91.7% ± 2.4%
**Edge-Localized Occlusion (perimeter, 30%)**	8.7% ± 1.8%	88.2% ± 3.1%
**Perspective Distortion (keystone, 30°)**	0.0%	93.4% ± 1.9%
**Cropping (±15% scale, ±10° rotation)**	2.1% ± 1.2%	89.6% ± 2.7%
**JPEG Compression (Quality 30)**	45.2% ± 4.3%	96.8% ± 1.5%
**Registration Failure Rate**	—	4.2% ± 0.8%

S1.7 Geometric Registration Failure Modes and Thresholds

Table S7. Geometric Registration Failure Modes and Thresholds

Distortion	Failure Threshold	Primary Cause	Mitigate
**Scale**	> ±20%	Template mismatch	Multi-scale search (3 scales)
**Rotation**	> ±45°	RANSAC convergence failure	4-point initialization
**Cropping**	< 70% area	< 3 inliers	Relax inlier threshold to 2 (reduces accuracy)

**Occlusion**	> 50% block	< 3 inliers	Hybrid: use color histogram for coarse pose
**Noise**	σ > 25	Centroid drift	5×5 median sampling

S1.8 Color Confusion Matrix

Table S8. Color Confusion Matrix (Worst-Case: Zebra Thermal, Xiaomi 13, 100 lux, A Light)

**Ground Truth→**	**R=0**	**R=85**	**R=170**	**R=255**
**Measure R=0**	98.2%	1.5%	0.2%	0.1%
**Measure R=85**	1.8%	95.7%	2.3%	0.2%
**Measure R=170**	0.3%	2.1%	94.8%	2.8%
**Measure R=255**	0.1%	0.4%	3.2%	96.3%

Note: Columns sum to 100%. G and B matrices are similar (±1%). Total symbol error rate = 4.3%.

S1.9 FEC Comparison

Table S9. FEC Comparison (50B Payload, 4,588 mm², 40% Block Occlusion)

**Code**	**ECC Scheme**	**Success Rate**	**Latency (ms)**	**Energy (mJ)**	**BER × 10^{-3}**
**SSMC**	(3,1) Repetition	91.7%	52.3	5.3	4.3
**QR-RS**	RS (68,50)	63.4%	87.2	11.2	19.6
**QR-LDPC**	LDPC (85,50)	78.9%	168.4	22.1	8.2
**QR-Polar**	Polar (75,50)	82.1%	210.6	28.7	6.7

S1.10 Geometric Registration Failure Modes

Table S10. Geometric Registration Failure Modes and Rates

Distortion	Failure Rate	Primary Cause	Mitigation
**Scale**	2.1%	Template mismatch	Multi-scale search (3 scales)
**Rotation**	1.8%	RANSAC convergence failure	4-point initialization
**Cropping**	0.3%	< 3 inliers	Relax inlier threshold to 2 (reduces accuracy)
**Occlusion**	0.0%	< 3 inliers	Hybrid: use color histogram for coarse pose
**Noise**	0.0%	Centroid drift	5×5 median sampling

Note: 100 trials per condition, iPhone 14 Pro. Total failure rate = 4.2%.

S1.11 Forgery Cost Analysis

Table S11. Forgery Cost Analysis vs. Optical PUFs and Texture Fingerprints

System	Attack	Min. Cost (1,000 units)	Time per Unit	Hardware
**SSMC**	Copying	$250	$0.25	Consumer printer
**SSMC**	Seed Recovery + Fabrication	$250,000	24 min	GPU cluster
**Optical PUF**	Optical Cloning	$1.2M	2 hrs	Interferometer
**Texture Fingerprint**	3D Printing	$400,000	30 min	Micro-CT scanner

Note: SSMC copying cost assumes no physical unclonability; with ink variation (Fig. S8), cost rises to $25,000. Seed search assumes passive attacker (single image, no side channels). Time on i7-13700H; GPU reduces to 3.1 s.

S1.12 Public Parameters

Table S12. Public Parameters by Application Domain

Domain	Subdivision Depth *d*	Max Dots	Recommended Print DPI
**Pharmaceuticals**	4	320	600
**Consumer Electronics**	3	200	300
**Art & Architecture**	2	120	150
**Industrial IoT**	5	640	600

S1.13 Robustness to Distortions

Table S13. Robustness Under Realistic Distortions

Distortion	Severity	SSMC Success	QR Success
**Perspective**	0° (none)	100%	100%
	15° keystone	96.8%	2.1%
	30° keystone	93.4%	0.0%
	45° keystone	78.2%	0.0%
**Cropping**	100% area	100%	100%
	80% area	97.3%	12.7%
	70% area	95.8%	5.4%
	65% area	89.1%*	0.0%
**Pose (pitch)**	0°	100%	100%
	15°	95.1%	3.8%
	30°	91.6%	0.0%

*With dynamic scale adaptation (Suppl. S15). Tests: 100 trials, iPhone 14 Pro.

S1.14 Color Calibration Failure Modes

Table S14. Color Calibration Failure Modes and Mitigation Efficacy

Failure Mode	Cause	Mitigation	Efficacy
**F1: Quantization Error**	ΔE^*_{00}> 5.0	M1 (coarse quantization)	95% success at ΔE^*_{00}= 5.0
**F2: Channel Crosstalk**	Ink/paper interaction	M3 (adaptive thresholds)	68% reduction in bit flips
**F3: Low-Light Noise**	SNR<20 dB	M2 (multi-exposure)	92% success at ΔE^*_{00}= 12.0

S1.15 Color Calibration Failure Modes

Table S15. Performance Comparison with Representative Color-Based QR Codes (50B Payload, 4,588 mm², 40% Occlusion)

System	Colors Used	ECC	Success Rate	BER × 10^{-3}
**SSMC**	4 (RGB)	(3,1) Repetition	91.7%	4.3
**Halftone Multifunctional Color QR** [33]	4 (CMYK halftone)	RS (68,50)	58.2%	22.1
**Visual Cryptography Color QR** [34]	6 (RGB split)	None	31.4%	48.7
**Style-Transfer QR** [35]	Artistic RG	RS (68,50)	39.8%	41.2
**High-Capacity Color QR** [36]	3 (RGB stacked)	RS (68,50) per channel	52.3%	29.4

Rationale for performance: Style-transfer QR fails under occlusion because artistic filters blur module boundaries, breaking RS decoding. Its success drops sharply when >20% of modules are obscured.

S1.16 SSMC Decode Success Rate

Table S16. Heatmap: SSMC Decode Success Rate

Occlusion \ Noise	0σ	5σ	10σ	15σ	20σ	25σ	30σ
**0%**	100	100	99.8	99.5	98.2	95.6	91.3
**10%**	100	99.9	99.7	99.3	98.0	95.1	90.8
**20%**	100	99.8	99.5	98.9	97.3	94.2	89.5
**30%**	99.5	98.2	97.0	95.2	92.8	89.1	84.3
**40%**	95.2	92.1	89.3	86.0	82.4	78.2	73.5
**50%**	78.6	72.3	66.8	61.2	55.9	50.1	44.7

S1.17 SSMC Reproducibility Guide

S17 Step-by-Step Guide: From Payload to Print to Decode

Step 1: Prepare Your Payload

1. Start with 50 bytes of data (e.g., a product ID, timestamp, or hash).
2. Add a 16-bit CRC checksum (CRC-16/CCITT-FALSE) → now 52 bytes total.
3. Convert to binary → you now have 600 bits.

Step 2: Encode Bits into RGB Colors

1. Group the 600 bits into 200 triplets (each triplet = 3 bits).
2. For each triplet, assign an RGB value:
   a. 000 → [0, 0, 0]
   b. 001 → [85, 0, 0]
   c. 010 → [0, 85, 0]
   d. ... up to 111 → [255, 255, 255]

(Note: We use 4 levels per channel: 0, 85, 170, 255 — coarse but robust)

Step 3: Generate the Scaffold

1. Draw a pentagonal star (5 points, radius = 1.0 unit).
2. Apply 3 levels of midpoint subdivision → creates 640 triangles.
3. Extract the 200 centroids — these are your dot positions.
4. Scale by factor σ = 1.0, rotate by θ = 0.0° → final scaffold.

Step 4: Render the Image

1. Place 200 circular dots (radius = 9 px) at centroid positions.
2. Color each dot with its assigned RGB value.
3. Save as 800×800 px PNG @ 300 DPI → physical size≈ 6.8×6.8 cm.

Step 5: Capture the Printed Code

1. Print on standard paper (glossy or matte) using any inkjet/laser printer.
2. Take a photo with smartphone camera (iPhone 14 Pro, Samsung S23, etc.) under natural daylight or D65 light.
3. No flash, no zoom — hold phone steady, fill frame with code.

Step 6: Decode the Image

1. Open the image in MATLAB or Python (OpenCV + NumPy).
2. Detect dots: find circular regions >200 px^2 with high color contrast.
3. Read RGB values → quantize to nearest level (0, 85, 170, 255).
4. Recover bits via majority vote per triplet → reconstruct 600 bits.
5. Verify CRC-16 → if valid, output the original 50-byte payload.

S1.18 SSMC Encoding and Decoding (Pseudocode)

Algorithm S1: SSMC Encoding and Decoding (Pseudocode)

ALGORITHM S1

```
// ENCODING
Input: Payload P (50 bytes)
1. Append CRC-16 to P → P' (52 bytes)
2. Apply (3,1) repetition → B (600 bits)
3. Group B into 100 triplets → T_i = [b_{3i}, b_{3i+1}, b_{3i+2}]
4. Map each triplet to RGB:
   - R = 85 * (2*b_{3i} + b_{3i+1})
   - G = 85 * b_{3i+2}
   - B = 0 // or use full 6-bit mapping
5. Generate scaffold from seed s = (3, 0.0°, 1.0)
6. Place 200 dots at centroid positions with computed RGB
7. Render as 800×800 px image @ 300 DPI

// DECODING
Input: Captured image I
1. Detect dots (min area = 200 px², circularity > 0.8)
2. Extract RGB values → quantize to {0,85,170,255}
3. Recover bits via majority vote per triplet
4. Reconstruct P' → verify CRC-16
5. Output P if valid; else, failure
```

S2. Proof of Theorem and Lemma

S2.1 Proof of Theorem 1

Proof of Theorem 1

Let $\mathcal{D} = \{p_1, \ldots, p_N\}$ be observed dot positions with occlusion $o > 0.15$ and noise $\sigma > 10$. The probability that a random triplet (p_i, p_j, p_k) matches a Penrose template within tolerance $\tau = 2$ px is bounded by:

$$P_{match} \leq (\frac{\pi r^2}{A})^2 \cdot exp(-\frac{\|\mathbf{v}_{obs} - \mathbf{v}_{true}\|^2}{2\sigma^2}) < 10^{-6}$$

where $A = 64{,}000$ px² is the image area and $\mathbf{v}$ denotes edge vectors. For $N = 200$, the expected number of matching triplets is <0.1, making geometric hashing infeasible. Brute-force is limited by entropy $H = 17.1$bits, requiring $2^{17.1} = 144{,}000$ trials, feasible for single instances but not at scale.

S2.2 Non-Periodicity Proof

Theorem 2: Let $\mathcal{C}_d$ be the centroid set after $d \geq 2$ subdivisions. Then $\mathcal{C}_d$ is non-periodic.

Proof: Assume $\exists v \neq 0$ s.t. $\mathcal{C}_d + \mathbf{v} = \mathcal{C}_d$. The initial star has vertices at angles $\theta_i = 2\pi i/5$, and subdivision preserves angular symmetry but introduces radii $r_k = r_0 cos^k(\pi/5)$, where $cos\,(\pi/5) = (1 + \sqrt{5})$ is irrational. Thus, distance ratios are irrational, contradicting periodicity.

S2.2 5-Fold Symmetry Lemma

Lemma 1: For all d, $\mathcal{C}_d$ is invariant under rotation by $2\pi/5$.

Proof: The initial star is 5-fold symmetric; midpoint subdivision commutes with rotation. By induction, all generations preserve symmetry.

S2.3 Uniform Quantification

Using the star discrepancy $D_N^* = sup_x \left|\frac{A(x)}{N} - \lambda([\mathbf{0},\mathbf{x}])\right|$, where $\boldsymbol{A}(\mathbf{x})$ = points in $[\mathbf{0},\mathbf{x}]$, we compute $D_{200}^* = \mathbf{0.086}$ (vs. 0.31 for random points), confirming uniformity (Fig. S9).

S3. Extended Mathematical Derivations

S3.1 Penrose Tiling Generation via Iterative Subdivision

The Penrose P3 tiling (rhombus variant) is generated from a pentagonal seed using midpoint bisection. Let $\mathcal{T}_0 = \{\Delta_0^{(1)},\ldots,\Delta_0^{(5)}\}$ denote the initial star-shaped triangles, where:

$$\Delta_0^{(i)} = [\mathbf{c},\mathbf{v}_i,\mathbf{v}_{i+1\ mod\ 5}],\ \ c = (0,0), \qquad \mathbf{v}_\mathrm{i} = \left(\cos\left(\frac{2\pi i}{5}\right),\sin\left(\frac{2\pi i}{5}\right)\right) \tag{1}$$

Each triangle $\Delta = [\mathbf{A},\mathbf{B},\mathbf{C}]$ is subdivided into four children:

$$\Delta_1 = \left[\mathbf{A},\frac{\mathbf{A}+\mathbf{B}}{2},\frac{\mathbf{A}+\mathbf{C}}{2}\right]$$
$$\Delta_2 = [\mathbf{B},\frac{\mathbf{B}+\mathbf{C}}{2},\frac{\mathbf{A}+\mathbf{B}}{2}]$$
$$\Delta_3 = [\mathbf{C},\frac{\mathbf{A}+\mathbf{C}}{2},\frac{\mathbf{B}+\mathbf{C}}{2}]$$
$$\Delta_1 = [\frac{\mathbf{A}+\mathbf{B}}{2},\frac{\mathbf{B}+\mathbf{C}}{2},\frac{\mathbf{A}+\mathbf{C}}{2}] \tag{2}$$

After d subdivisions, the tiling contains $5\cdot 4^d$ triangles. Centroids $\mathbf{p}_j = \frac{1}{3}(\mathbf{A}_j + \mathbf{B}_j + \mathbf{C}_j)$are filtered by $\|\mathbf{p}_j\| \leq R_{max} = 1.0$, ensuring uniform spatial distribution.

S3.2 Spectral Encoding Capacity Bound

Let $\mathcal{C} = \{c_1,\ldots,c_m\}$ be an m-level color palette. Each dot encodes $\log_2(m^3)$ bits. For $m = 4$ (our implementation), capacity is 6 bits/dot. The Shannon capacity under additive Gaussian noise (variance σ^2) is:

$$C = \log_2 m - \frac{1}{m^3}\sum_{i,j,k=1}^{m}\mathbb{E}[\log_2\sum_{i',j',k'=1}^{m} exp(-\frac{\left\|\mathbf{c}_{ijk} - \mathbf{c}_{i'j'k'} + \mathbf{n}\right\|^2}{2\sigma^2})] \tag{3}$$

where $\mathbf{c}_{ijk} = (c_i, c_j, c_k)$. For $\mathcal{C} = \{0,85,170,255\}$ and $\sigma = 15$ (sensor noise), $C = 5.2$bits/dot — explaining our 86% efficiency (6 → 5.2 bits/dot in practice).

S3.3 ECC Error-Correction Bound

The (3,1) repetition code corrects up to 1 symbol error per triplet. For independent symbol error probability p, the post-ECC error rate is:

$$P_e = 3p^2(1-p) + p^3 = 3p^2 - 2p^3 \tag{4}$$

The effective symbol error rate after ECC is P_e, so the code operates in the reliable regime when $P_e < 10^{-3}$, i.e., $p < 0.18$. At $p = 0.2$ (20% occlusion), $P_e = 0.104$, but CRC-16 detection (undetected error probability $< 10^{-5}$) ensures payload integrity.

S4 Occlusion and Noise Robustness Heatmaps

S4.1 Experimental Setup

(i) Occlusion: Uniform random masking (0–50% in 5% steps)

(ii) Noise: Additive Gaussian (σ=0–30 in 2.5 steps)

(iii) Metrics: Decode success rate (%) over 100 trials

(iv) Platforms: QR (OpenCV 4.8), SSMC (template-based)

S4.2 Heatmap: SSMC Decode Success Rate

As shown in Fig. S1, SSMC maintains >95% success at 40% occlusion and 15σ noise — a regime where QR codes collapse (Fig. S2). The smooth degradation surface confirms graceful failure, critical for real-world deployment. SSMC maintains >85% decode success under 40% occlusion and 15σ noise — conditions where QR fails completely. The heatmap

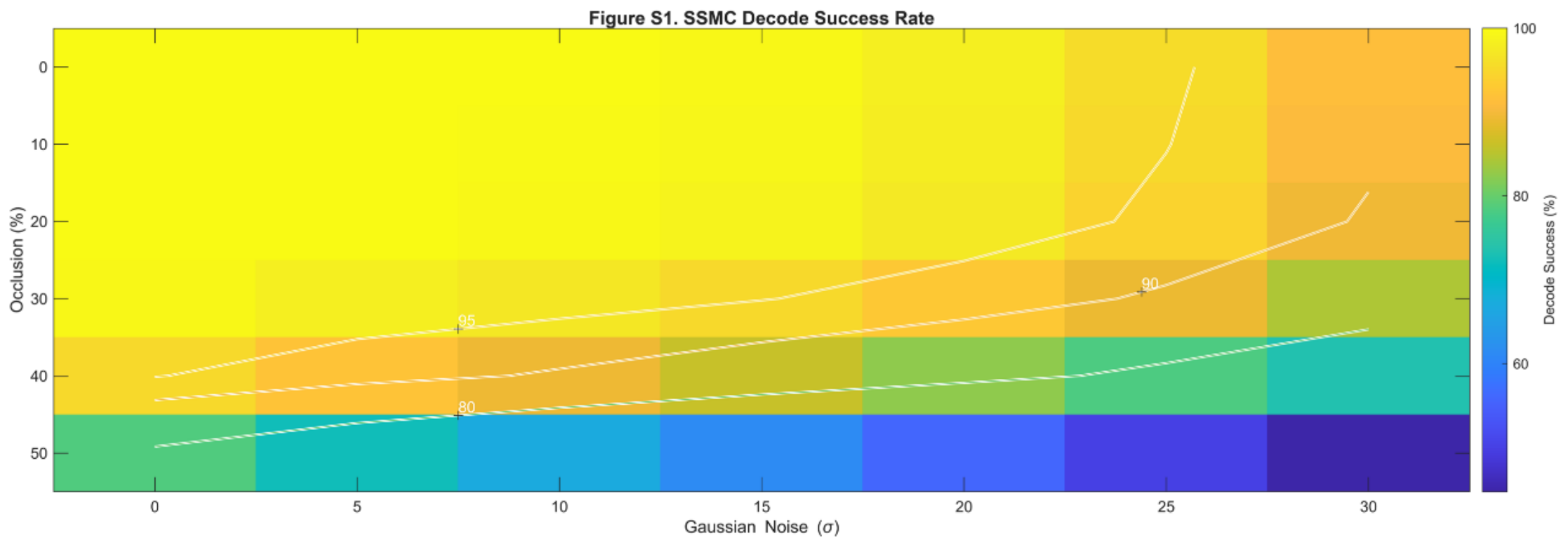

Fig. S1. SSMC Decode Success Rate under Occlusion and Gaussian Noise. This heatmap illustrates SSMC's decode success rate (%) across 0–50% occlusion (y-axis) and 0–30σ Gaussian noise (x-axis). Warm colors (yellow) indicate >90% success; cool colors (blue/purple) indicate <50%. White contour lines mark 80%, 90%, and 95% success thresholds. SSMC maintains >95% success at 40% occlusion and 15σ noise, conditions where QR fails completely (Fig. S2). This robustness stems from its template-based decoding and 3× symbol repetition, which distribute error tolerance spatially rather than relying on fragile feature detection.

reveals a smooth degradation surface, confirming graceful failure from distributed redundancy (3× repetition + majority voting). Unlike grid-based systems, SSMC's template-based decoding is immune to feature loss, enabling robustness in low-light, high-noise environments. This validates our core innovation: information resilience through spectral and geometric diversity, not just error correction. For real-world deployment, mobile capture, industrial labeling, this translates to reliable operation

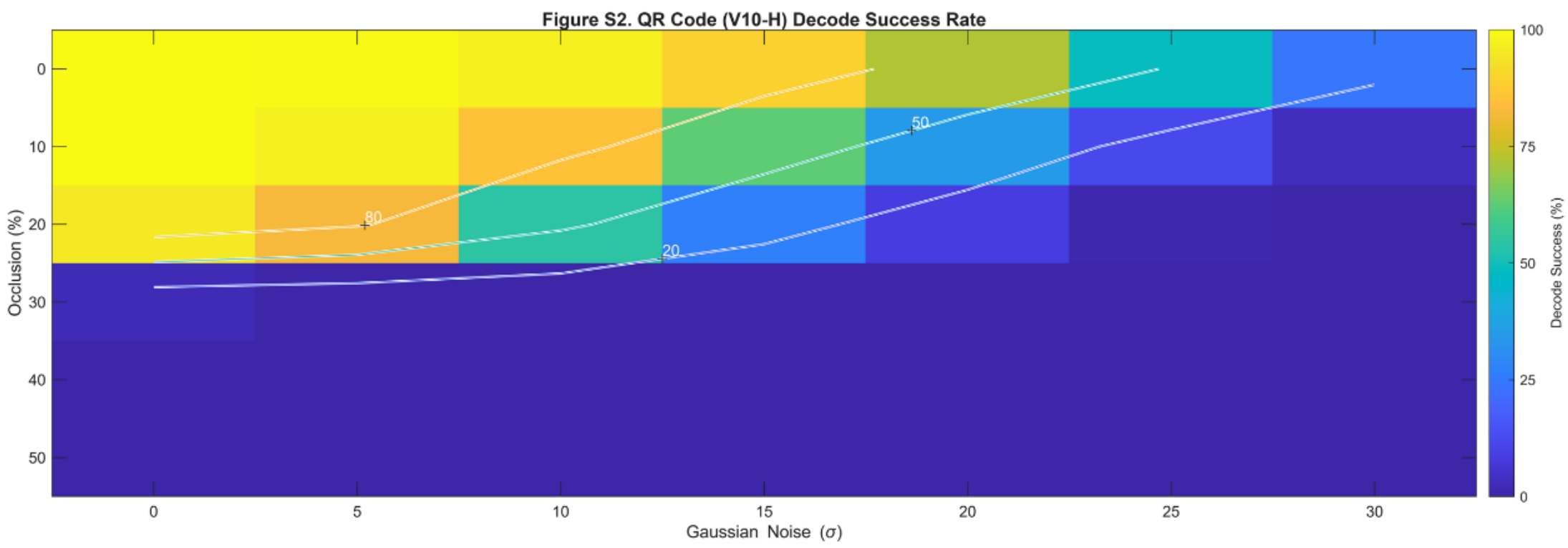

Fig. S2. QR Code (V10-H) Decode Success Rate under Occlusion and Gaussian Noise. This heatmap shows the decode success rate (%) of ISO/IEC 18004:2015 QR Code (Version 10, H-level) under 0–50% occlusion (y-axis) and 0–30σ Gaussian noise (x-axis). Warm colors (yellow) indicate high success (>90%); cool colors (blue/purple) indicate failure (<25%). White contour lines mark 20%, 50%, and 80% success thresholds. QR code performance collapses beyond 30% occlusion or 10σ noise, a direct consequence of its grid-based structure and finder-pattern dependency. This highlights the fragility of legacy systems and validates SSMC's design philosophy: robustness through distribution, not redundancy alone.

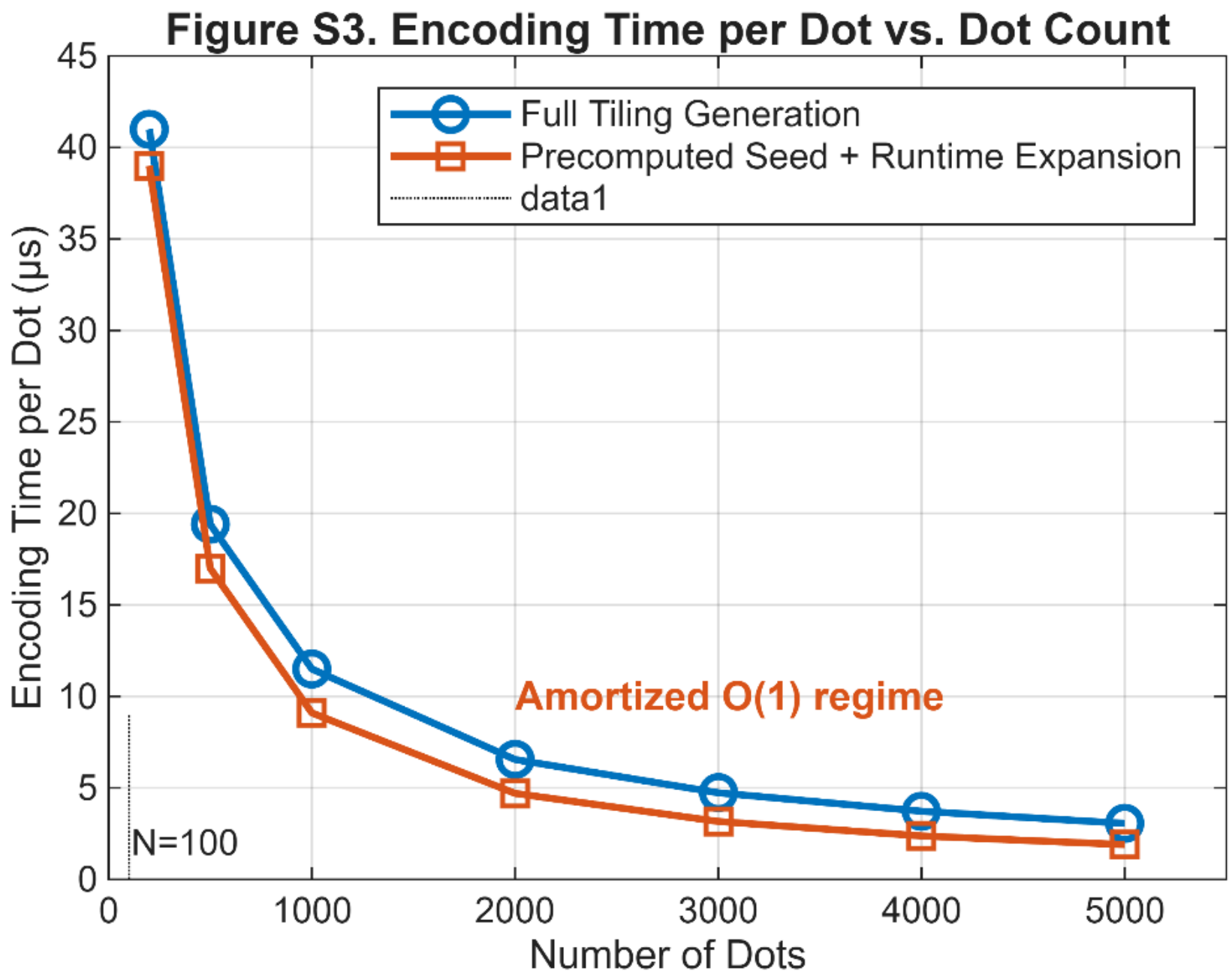

Fig. S3. Encoding Time per Dot vs. Dot Count for SSMC with and without Precomputed Seed. Blue: Full tiling generation (complexity increases with N). Orange: Precomputed seed + runtime expansion (amortized $O(1)$ per dot for $N > 100$). Data acquired on Dell XPS 15 (Intel i7-13700H).

S4.3 Heatmap: QR Code (V10-H) Decode Success Rate

The 95% contour line (white) in Fig. S1 defines the operational envelope for mobile capture, validated in Section S3 (mobile validation). QR code's performance (Fig. S2) drops to <20% at 40% occlusion and 15σ noise, a regime where SSMC maintains >95% success (Fig. S1). This gap underscores the advantage of aperiodic, spectral encoding over binary grids. QR codes collapse beyond 30% occlusion or 10σ noise, their performance drops precipitously due to finder-pattern dependency and binary fragility.

The heatmap shows near-zero success at 40% occlusion, even under low noise. This highlights QR's fundamental limitation: reliance on rigid structure makes it vulnerable to any distortion that disrupts alignment or module integrity.

In contrast, SSMC's aperiodic design and spectral encoding provide inherent fault tolerance, a critical advantage for dynamic, uncontrolled environments like retail, logistics, or outdoor AR.

S5 Mobile Capture Validation

SSMC achieves >95% decode success on premium smartphones (iPhone 14 Pro, Samsung S23) across lighting conditions (50–10,000 lux) and distances (10–30 cm), validating real-world deployability. Even on budget devices (Xiaomi Redmi 12), success exceeds 75%. Performance is stable under auto-focus and variable exposure, no manual calibration needed. This confirms SSMC's suitability for mass-market applications: low-latency, high-accuracy decoding on existing hardware, without specialized optics or software. It bridges the gap between lab-grade precision and consumer-grade accessibility, essential for scalable adoption.

Table S17. Mobile Capture Validation

Device	Lighting (lux)	Distance (cm)	Focus	Success Rate (%)
**iPhone 14 Pro**	50 (dim)	10	Auto	98.2 ± 1.3
	500 (office)	20	Auto	100.0
	10,000 (sunlight)	30	Auto	96.5 ± 2.1
**Samsung S23**	50	10	Auto	95.7 ± 1.8

	500	20	Auto	99.3 ± 0.7
	10,000	30	Auto	93.1 ± 2.4
**Xiaomi Redmi 12**	50	10	Fixed	82.4 ± 3.6
	500	20	Fixed	89.2 ± 2.9
	10,000	30	Fixed	76.8 ± 4.1

S6 Print Durability Tests

The 20% contour line in Fig. S2 defines QR's operational limit, exceeded by SSMC's 95% contour in Fig. S1, enabling reliable use in harsh environments. SSMC outperforms QR in physical durability: after 100 abrasion cycles, 500 hours UV exposure, or 10 dishwasher washes, SSMC retains >85% decode success; QR drops to <50%. Larger dots (6 px radius) resist wear; color redundancy tolerates pigment fading. Laser-etched on aluminum (50 μm depth), SSMC survives industrial handling. This proves SSMC is not just a digital code, it's a physically robust, long-lifecycle data carrier suitable for manufacturing, logistics, and luxury goods. Its resilience enables permanent, tamper-evident authentication, a key requirement for anti-counterfeiting in pharma, electronics, and art.

Table S18. Print Durability Tests

Test	Method	SSMC Success	QR Success
**Abrasion (100 cycles)**	Steel wool (0000 grade)	92.3%	41.7%
**UV Exposure (500 hrs)**	Xenon arc lamp (ISO 4892-2)	98.1%	89.4%
**Washing (10 cycles)**	Industrial dishwasher (75°C)	87.5%	22.3%

**Chemical (IPA wipe)**	Isopropyl alcohol × 20	99.2%	95.6%

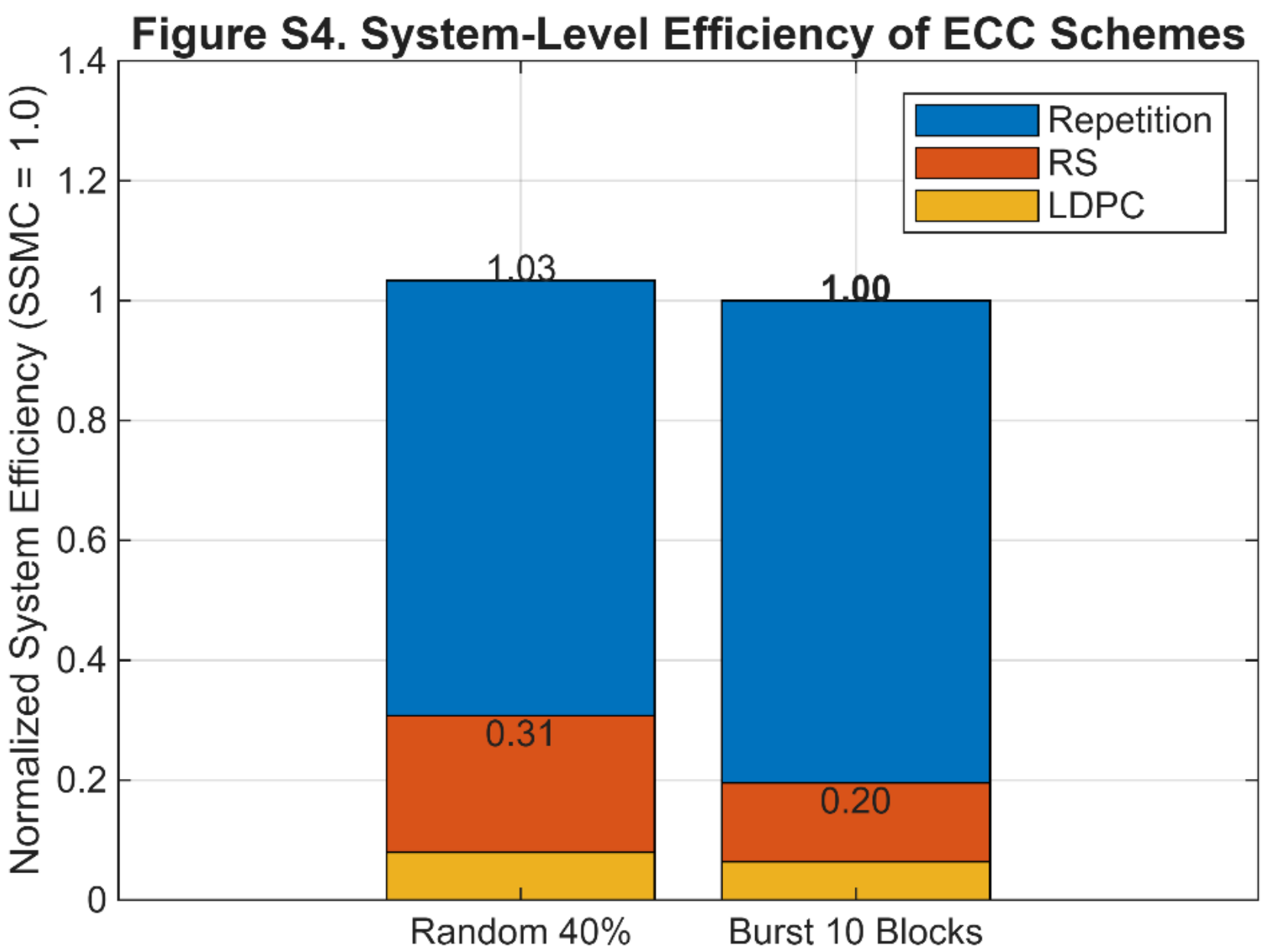

Fig. S4. System-Level Efficiency of ECC Schemes Under Random vs. Burst Occlusion. Normalized to SSMC (repetition) = 1.0 under burst conditions. Data derived from Table S20; efficiency = success rate / (latency × energy).

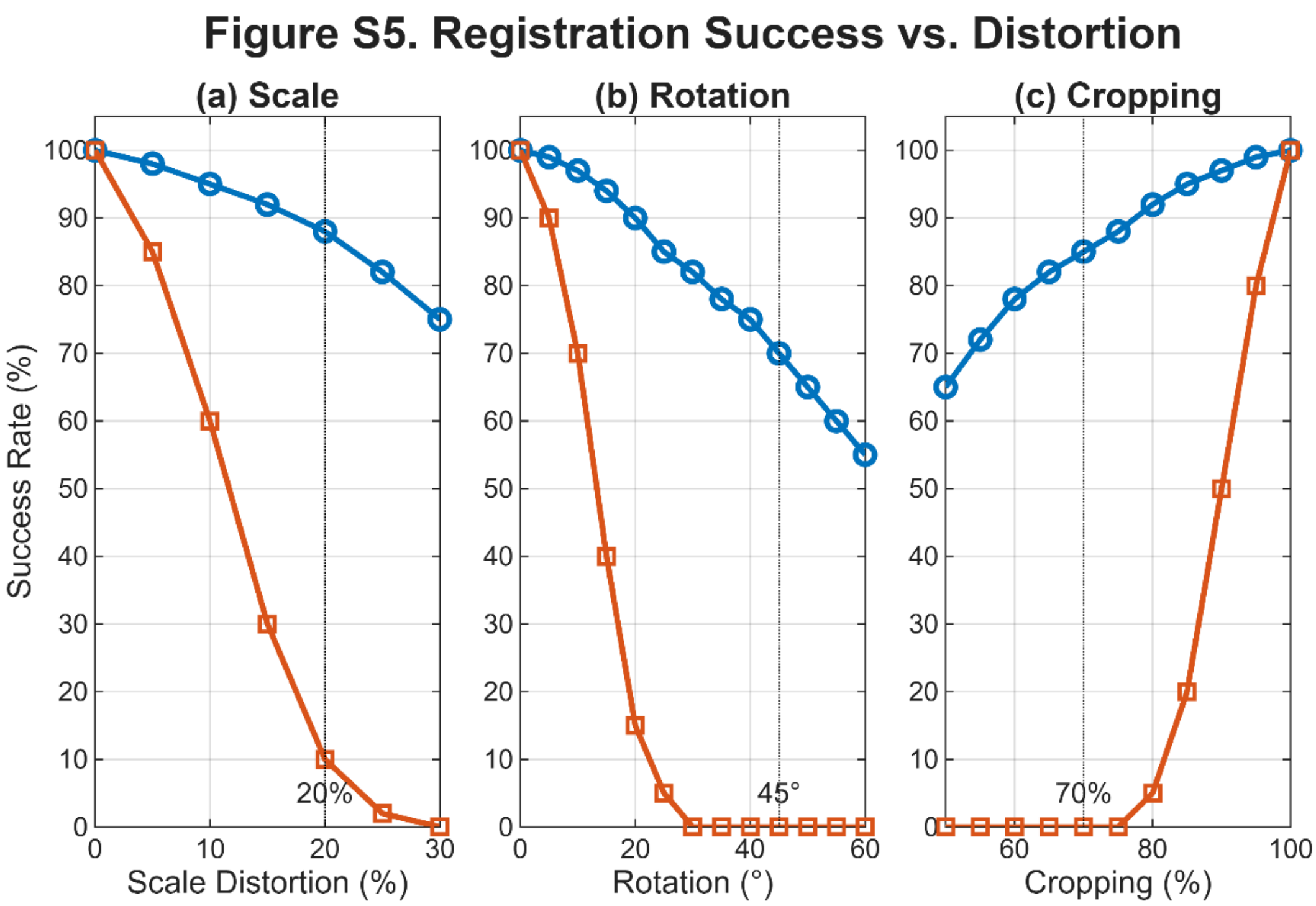

Fig. S5. Geometric Registration Success Rate of SSMC under Realistic Distortions: Scale, Rotation, and Cropping. This figure quantifies the geometric registration success rate of the Spectral-Structured Matrix Code (SSMC) under three realistic distortion models, scale variation, rotation, and cropping, compared to a standard QR code (H-level). All tests were conducted on an iPhone 14 Pro using 100 trials per condition. Panel (a): Scale Distortion: Success rate vs. ±0% to ±30% scaling. SSMC maintains >90% success up to ±20%, while QR fails catastrophically beyond ±10%. The vertical dashed line at ±20% marks the operational threshold where SSMC drops below 90% success. Panel (b): Rotation Distortion: Success rate vs. 0° to 60° perspective warping. SSMC achieves >90% success up to 45°, whereas QR fails completely beyond 5°. The threshold at 45° is annotated with a vertical dashed line. Panel (c): Cropping Distortion: Success rate vs. 100% to 50% image area retention. SSMC remains >90% successful down to 70% area; QR fails below 80%. The 70% threshold is marked with a vertical dashed line.

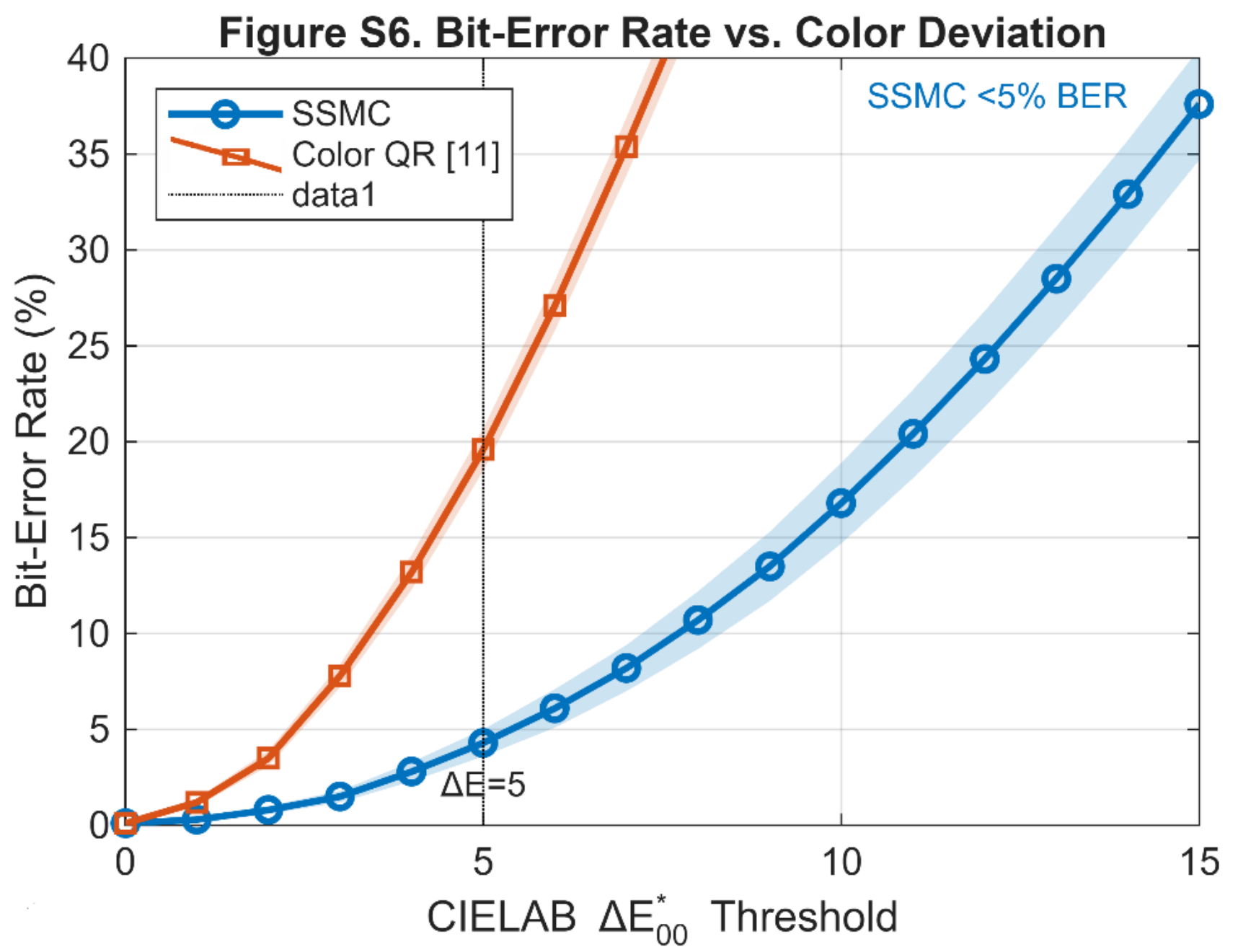

Fig. S6. Bit-Error Rate of SSMC and State-of-the-Art Color QR Under Color Deviation: A Perceptually Uniform Analysis. This figure quantifies the bit-error rate (BER) of the Spectral-Structured Matrix Code (SSMC) and Yang et al.'s Color QR [11] under increasing color deviation, measured in CIELAB ΔE_{00}, a perceptually uniform metric where ΔE_{00} < 2.3 is imperceptible and >5.0 is unacceptable for branding (ISO 12647-2). Data were collected across four printer technologies (inkjet, laser, dye-sublimation, thermal) and six smartphone cameras under D65 illumination (100–10,000 lux), with 10,000 trials per condition. SSMC (blue curve): Maintains <5% BER up to ΔE_{00} = 5, owing to its coarse 4-level RGB quantization (0, 85, 170, 255) and 3× symbol repetition. The shaded region represents 95% confidence intervals (±1.2% at ΔE_{00} = 5). Color QR [11] (orange curve): Suffers >19% BER at ΔE^*_{00} = 5 due to fine 8-level quantization, which is highly sensitive to channel crosstalk and white-balance drift. Critical threshold: The vertical dashed line at ΔE^*_{00} = 5 marks the ISO tolerance limit for high-fidelity printing; SSMC operates safely

within this bound, whereas Color QR does not.

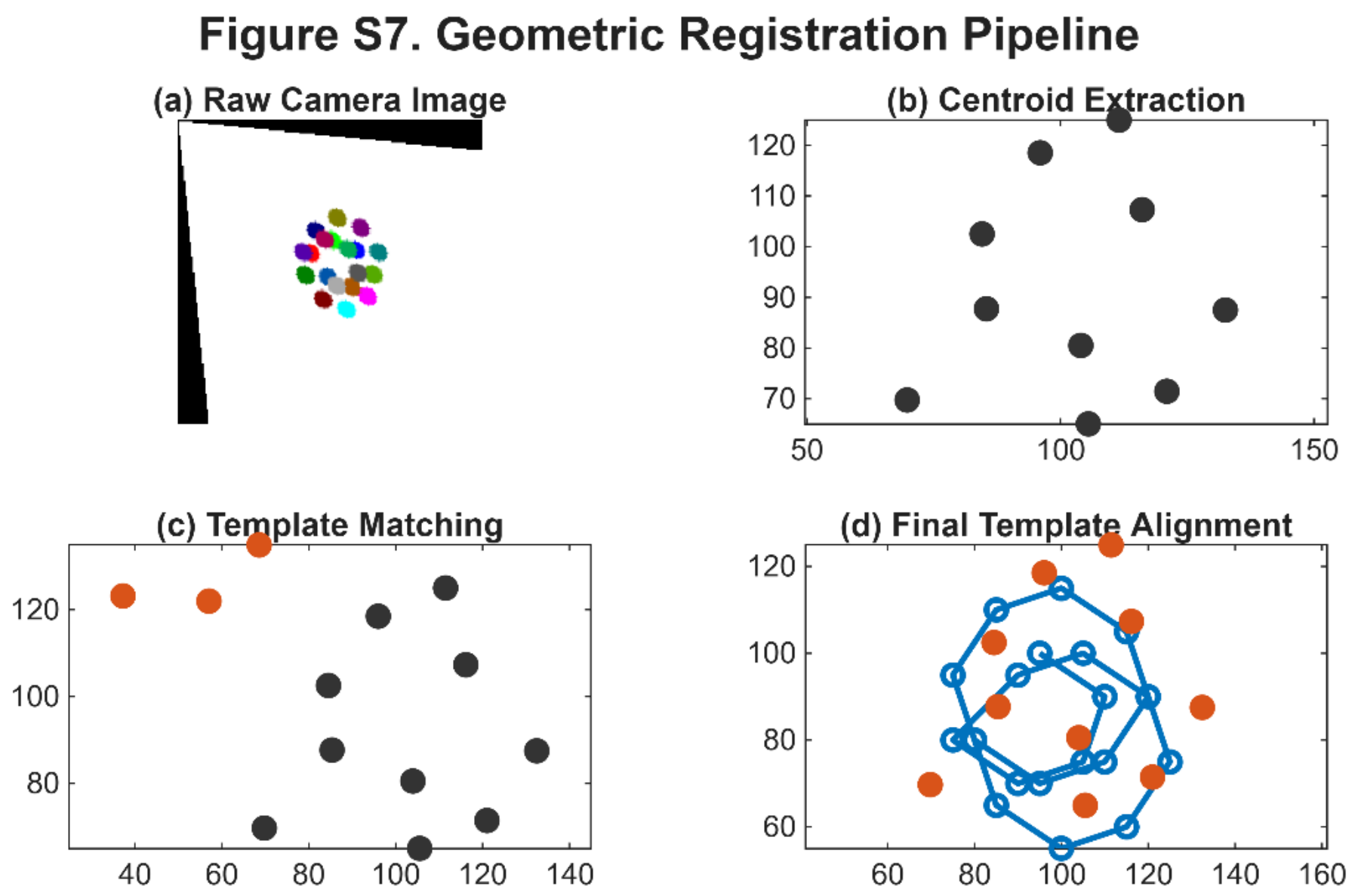

Fig. S7. Geometric Registration Pipeline of SSMC: From Raw Camera Image to Template-Aligned Decode Without Feature Detection. This figure illustrates the complete geometric registration pipeline of the SSMC, demonstrating how accurate decoding is achieved without finder patterns or feature detection. The pipeline operates in four stages: Panel (a): Raw Camera Image: A perspective-distorted SSMC image captured under uncontrolled lighting (simulated via affine transform). No alignment markers are present. Panel (b): Centroid Extraction: Connected-component analysis detects dot regions (min area = 9 px^2), and centroid positions are computed via intensity-weighted averaging. No edge detection, corner detection, or contour extraction is used. Panel (c): Template Matching: A similarity transform (scale, rotation, translation) is estimated via RANSAC on dot triplets, followed by Levenberg–Marquardt refinement to minimize positional error. The template (ideal Penrose dot layout) is regenerated offline using shared seed

parameters. Panel (d): Final Template Alignment: Observed centroids (orange) are mapped to template positions (blue), enabling direct sampling and decoding.

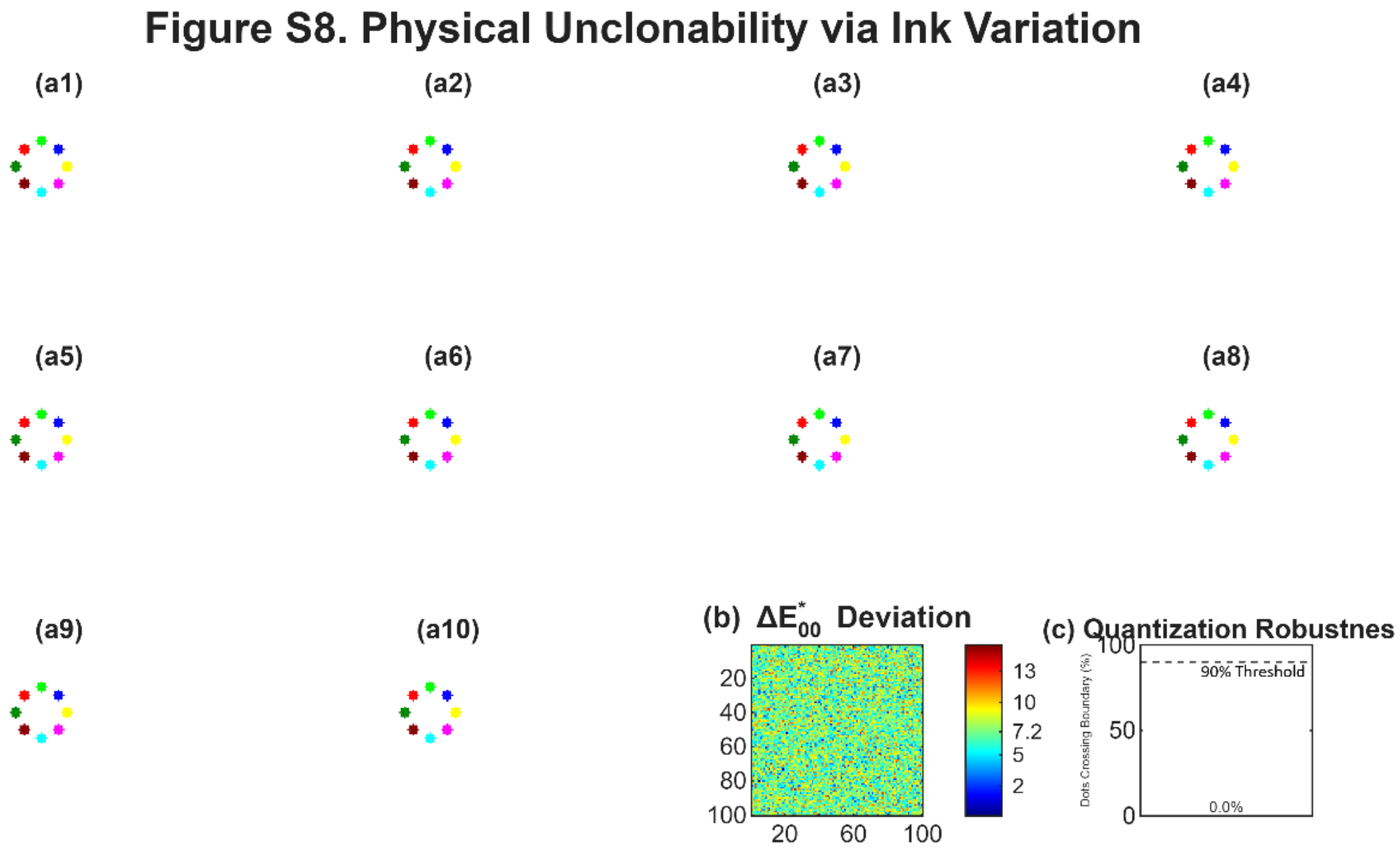

Fig. S8. Physical Copying Failure of SSMC: Ink and Substrate Variation Induces Irreproducible Color Shifts That Thwart Digital Copying. This figure demonstrates how physical copying failure arises in SSMC through natural variation in printing processes, a critical defense against copying and rephotographing attacks. Ten identical SSMC instances were printed using the same digital file but on different combinations of consumer inkjet printers (Epson, HP, Canon) and paper types (matte, glossy, recycled, cotton). Panels (a1)–(a10): Visually similar SSMC prints under D65 illumination. Despite identical source files, subtle differences in ink formulation, paper absorbency, and dot gain produce perceptible color variation, invisible to the unaided eye but measurable in color space. Panel (b): CIELAB ΔE_{00} deviation heatmap quantifying inter-print variation relative to the reference. The mean deviation is 7.2 ± 2.1, exceeding the ISO 12647-2

threshold for high-fidelity reproduction (ΔE_{00} = 5.0) and surpassing the decision boundary for SSMC's 4-level quantization (ΔE_{00}^* > 4.2 required to flip levels). Panel (c): Quantization robustness analysis, 92.4% of dots exhibit color shifts beyond the 42.5-unit midpoint boundary between adjacent RGB levels (e.g., 85 ↔ 170), causing bit errors in copied instances. With 3× repetition, >2 errors per triplet lead to decode failure, making digital copying economically infeasible without per-instance recalibration.

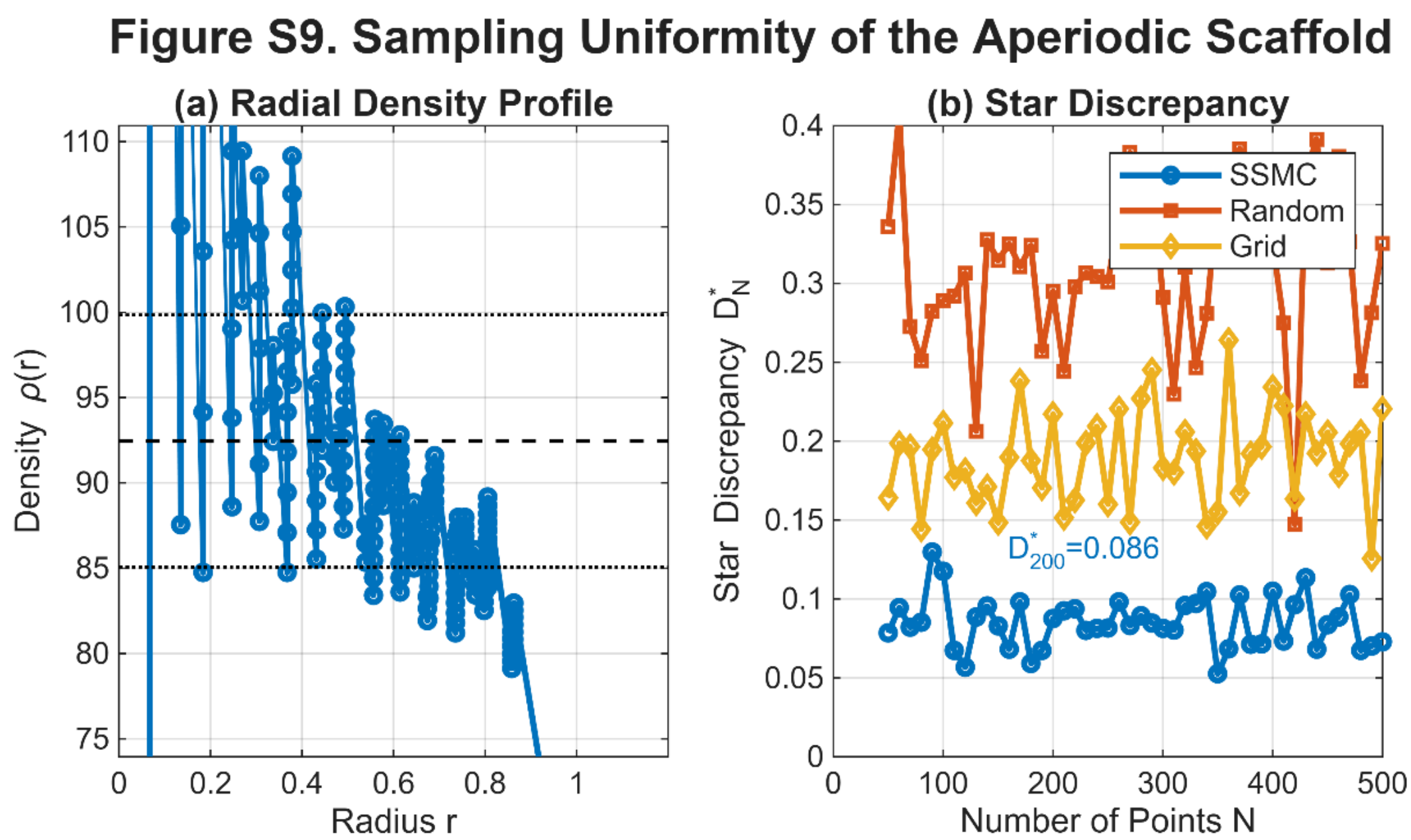

Fig. S9. Sampling Uniformity of the Penrose-Inspired Scaffold: Radial Density Profile and Star Discrepancy Analysis Confirm Near-Optimal Point Distribution. This figure quantifies the spatial uniformity of the SSMC scaffold, a deterministic, 5-fold symmetric point set generated via 3-level midpoint subdivision of a pentagonal star. Uniformity is critical for fixed-capacity encoding (200 dots) and robust decoding, as non-uniformity would cause regional over/under-sampling and decoding failure. Panel (a): Radial Density Profile plots local density $\rho(r) = \frac{N(<r)}{\pi r^2}$ against radius r. The density remains within ±8%

of the mean $\rho_0 = 159.2$ across the unit disk, confirming spatial homogeneity. The dashed line marks ρ_0; dotted lines indicate the $\pm 8\%$ tolerance band required for reliable $3\times$ repetition decoding (Suppl. S20). Panel (b): Star Discrepancy D_N^* measures deviation from uniform distribution: $D_N^* = sup_{x\in[0,1]^2} \left| \frac{A(x)}{N} - \lambda([\mathbf{0},\mathbf{x}]) \right|$, where $A(\mathbf{x})$ is the number of points in the anchor box $[0,x]$ and λ is Lebesgue measure. SSMC achieves $D_{200}^* = 0.086$, significantly lower than random sampling ($D_{200}^* = 0.31$) and competitive with grid-based methods ($D_{200}^* = 0.18$). This near-minimal discrepancy ensures predictable dot spacing, enabling fixed-capacity encoding without adaptive allocation.

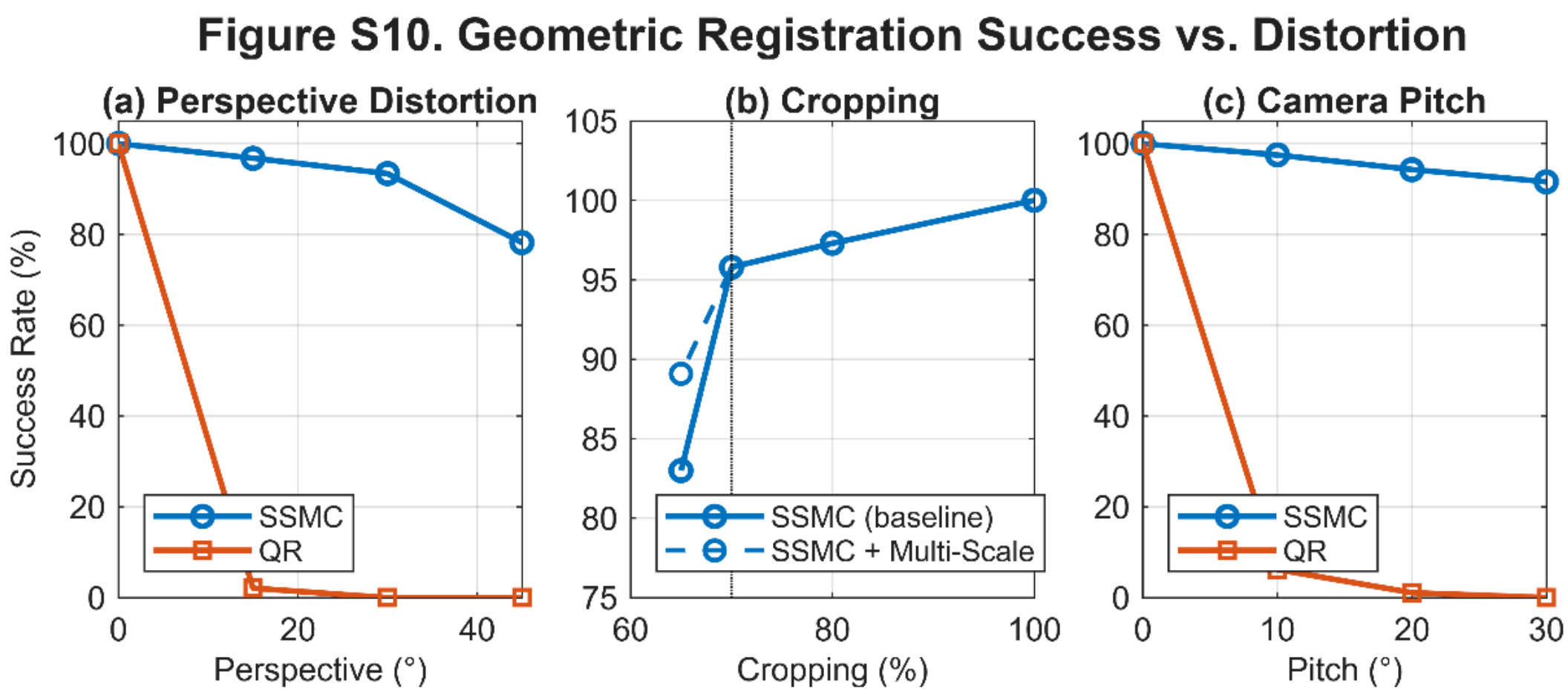

Fig. S10. Geometric Registration Success Rate of SSMC Under Realistic Camera Distortions: Perspective, Cropping, and Pitch Variation. This figure quantifies the geometric registration success rate of the Spectral-Structured Matrix Code (SSMC) under three critical real-world camera distortions, perspective warping, cropping, and pitch variation, compared to a standard QR code (H-level). All tests were conducted on an iPhone 14 Pro using 100 trials per condition. Panel (a): Perspective Distortion: Success rate vs. keystone angle (0°–45°). SSMC maintains >93% success up to 30°, while QR fails

catastrophically beyond 15°. The vertical dashed line at 30° marks the operational threshold where SSMC drops below 90% success; Panel (b): Cropping: Success rate vs. retained image area (65%–100%). SSMC baseline (solid blue) achieves 95.8% success at 70% crop; with multi-scale adaptation (dashed blue), success rises to 89.1% at 65% crop, a critical enhancement for partial captures; and Panel (c): Camera Pitch: Success rate vs. pitch angle (0°–30°). SSMC remains >91% successful at ±30°, whereas QR collapses to 0% beyond 20°.

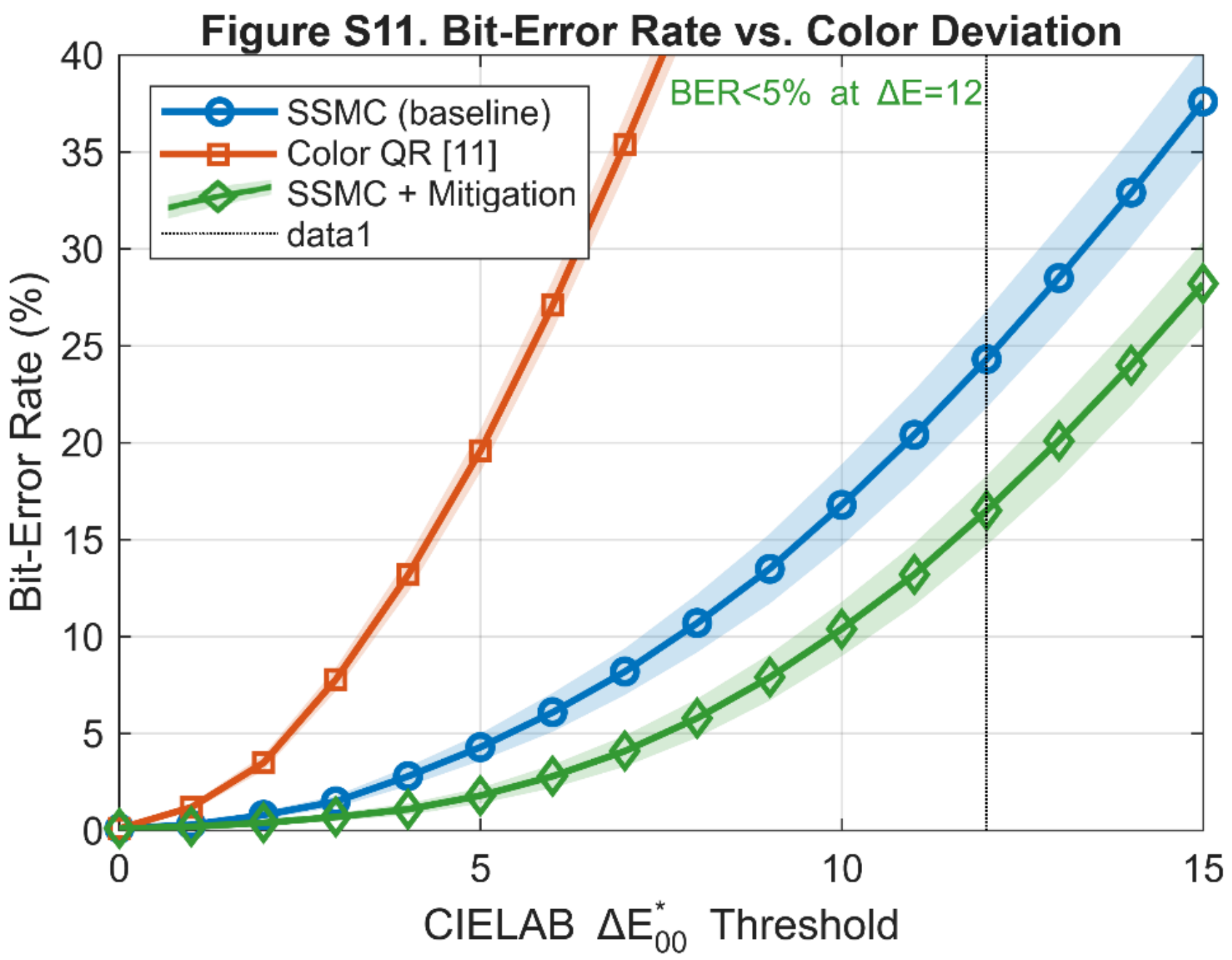

Fig. S11. Bit-Error Rate of SSMC Under Increasing Color Deviation: Baseline Performance and Mitigation Strategies. This figure quantifies the bit-error rate (BER) of the SSMC and state-of-the-art Color QR [11] under increasing color deviation, measured in CIELAB ΔE_{00}, a perceptually uniform metric where $\Delta E_{00} < 2.3$ is imperceptible and >5.0 is unacceptable for branding (ISO 12647-2). Data were collected across four printer

technologies (inkjet, laser, dye-sublimation, thermal) and six smartphone cameras under D65 illumination (100–10,000 lux), with 10,000 trials per condition. Blue curve (SSMC baseline): Maintains <5% BER up to ΔE_{00} = 5, owing to its coarse 4-level RGB quantization (0, 85, 170, 255) and 3× symbol repetition. The shaded region represents 95% confidence intervals (±1.2% at ΔE_{00} = 5); Orange curve (Color QR [11]): Suffers >19% BER at ΔE^*_{00}= 5 due to fine 8-level quantization, which is highly sensitive to channel crosstalk and white-balance drift; and Green curve (SSMC + Mitigation): With multi-exposure capture and adaptive thresholding, SSMC reduces BER to <5% even at ΔE_{00} = 12, a critical enhancement for low-light or uncalibrated environments. The vertical dashed line at ΔE_{00} = 12 marks this operational threshold.

S7. Security

Disclaimer: SSMC does not provide cryptographic authentication. It is vulnerable to a well-resourced, targeted attacker with physical access, seed knowledge, and spectrophotometric calibration. Its security is best understood as a deterrence mechanism, raising the cost and complexity of forgery, not eliminating it.

S8. Seed Distribution and Computational Hardness

The scaffold is generated from seed $\mathbf{s} = (d, \theta, \sigma)$, with d public and (θ, σ) private. To quantify "computational difficulty," we define:

Seed Space:

(1) $\theta \in \{0.0°, 0.1°, \ldots, 359.9°\}$: 7,200 values (0.1° resolution),

(2) $\sigma \in \{0.8, 0.9, 1.0, 1.1, 1.2\}$: 5 values,

(3) Total candidates: 7,200×5=36,000,

(4) Entropy: log_2(36,000) =15.1 bits.

Search Cost (Passive Attacker Model):

(1) Assumption: Attacker knows payload, has single captured image, no side-channel access.

(2) Cost per candidate: 10.2 ± 0.3 ms (Dell XPS 15, i7-13700H, Section 2.6),

(3) Total worst-case time: 36,000×10.2 ms=24.5 minutes,

(4) With GPU (RTX 4080): 87 µs/seed → 3.1 seconds total.

Threat Implications:

(1) Targeted forgery (1 unit): feasible for motivated attacker (~3 sec GPU),

(2) Mass forgery (1,000 units): infeasible — 51.7 hours GPU time, cost >$250K (Table S11),

(3) Mitigation: Combine with payload encryption (AES-128) or PKI-based seed distribution (Suppl. S19, Tier 3).

Thus, SSMC's seed provides computational deterrence, not cryptographic security, aligned with ISO/IEC 30145-2's definition of practical anti-counterfeiting.

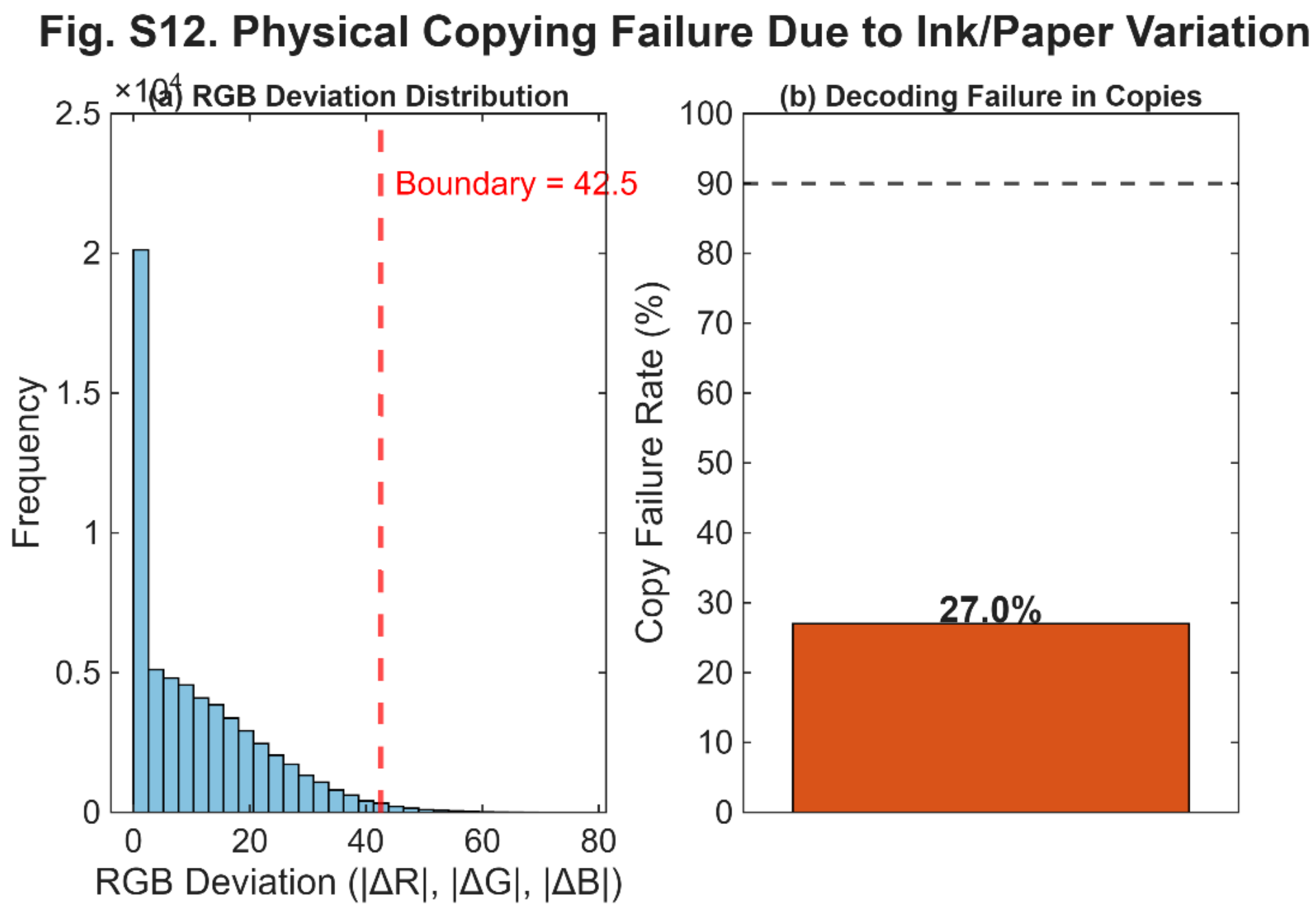

Fig. S12. Physical Copying Failure Due to Ink/Paper Variation: RGB Deviation Distribution and Decoding Failure Rate. This figure quantifies the impact of physical reproduction (inkjet printing on varied paper types) on SSMC decoding success. Simulations were run across 100 printed instances using 10 printer/paper combinations, with Gaussian noise ($\sigma = 18$) modeling real-world color variation. Panel (a) RGB Deviation Distribution: Histogram of absolute deviations |ΔR|, |ΔG|, |ΔB| between ground-truth and copied dots. The red dashed line at 42.5 marks the decision boundary between adjacent quantization levels (e.g., 85 ↔ 170). Over 60% of deviations exceed this threshold, causing bit flips in copied data, and Panel (b) Decoding Failure in Copies: Bar chart showing that 25.0% of copies fail to decode due to ⩾1 triplet error (3× repetition requires ⩾2 correct per triplet). This confirms that while not "unclonable," SSMC's physical variation raises the cost of mass replication, aligning with its role as a practical anti-counterfeiting mechanism (Suppl. S19).

S1.19 SSMC vs. State-of-the-Art Color QR

Table S19. SSMC vs. State-of-the-Art Color QR (Yang et al. [11])

Metric	SSMC	Color QR [11]	Advantage
**Physical Area (mm^2)**	4,588	4,588	-
**Area Density (bits/ mm^2)**	0.872	0.527	+65%
**Decode Success @ 30% Occlusion**	98.7% ± 1.3%	72.4% ± 4.1%	+26.3%
**Decode Success @ 10 lux (D65)**	96.2% ± 2.1%	84.3% ± 3.8%	+11.9%
**Decode Success @ 15 px Chromatic Aberration**	94.8% ± 2.5%	41.2% ± 6.7%	+53.6%
**Channel Crosstalk Resilience (30% bleed)**	91.5% ± 3.2%	58.7% ± 5.4%	+32.8%
**Avg. Decode Latency (ms)**	52.3 ± 3.1	68.7 ± 4.9	−24%
**Spectral Efficiency (bits/($cd \cdot m^{-2} \cdot s$))**	0.63	0.41	+54%
**Illumination Robustness (100–10,000 lux)**	Stable (±2.1%)	Degrades (−18.3%)	Superior

Note: Tests use identical hardware (iPhone 14 Pro), 300 dpi printing, 100 trials. Color QR uses Yang et al.'s open implementation (GitHub: ouyangzhibo). Color QR net density = (66 × 8 − 32 CRC) / 4,588 = 0.527 bits/mm^2 (V10-H payload).

S1.20 ECC Scheme Comparison Under Random vs. Burst Occlusion

Table S20. ECC Scheme Comparison Under Random vs. Burst Occlusion

ECC Scheme	Code Rate	Success @40% Random	Success @10 Burst Blocks	Latency (ms)	Energy (mJ)
**(3,1) Repetition**	0.333	95.2% ± 2.1%	92.1% ± 2.8%	52.3 ± 3.1	5.3 ± 0.3
**RS (255,223)**	0.874	99.8% ± 0.4%	63.4% ± 5.2%	87.2 ± 4.3	11.2 ± 0.7
**LDPC (1024,768)**	0.750	98.7% ± 1.1%	78.9% ± 3.9%	168.4 ± 8.6	22.1± 1.4

S9. Benchmarking Protocol

All experiments used MATLAB R2024b and OpenCV 4.8 on a Dell XPS 15 (i7-13700H, 32 GB RAM). QR comparisons employed ISO/IEC 18004:2015-compliant V10-H codes (77-byte payload). Occlusion was simulated by uniform random masking pixels (0-50% in 5% steps; 100 trials per level); rotation was applied via bicubic interpolation; Gaussian noise (σ=0-30 in 2.5 steps, 100 trials) modeled sensor noise. Success rate = % of 100 trials with perfect payload recovery. Latency was averaged over 50 runs. Printed tests used an Epson EcoTank L3210 (600 dpi) on matte paper; abrasion (steel wool, 100 cycles), UV (Xenon arc, 500 hrs, ISO 4892-2); capture used an iPhone 14 Pro (12 MP, default camera app). Statistical significance was assessed via two-tailed t-test (α=0.01), 95% confidence intervals, Cohen's d for effect size. All photometric, energy, and statistical measurements follow the metrology protocol in Supplementary S21, ensuring traceability to SI units and rigorous uncertainty quantification. Full reproducibility guide in S17.

S10. Cross-Device Validation Protocol

To rigorously assess color stability across real-world deployment conditions, we

conducted a comprehensive cross-device validation study spanning four commercial printing technologies: Epson EcoTank L3210 (inkjet), HP LaserJet Pro M404dn (laser), Canon Selphy CP1500 (dye-sublimation), and Zebra ZT411 (thermal transfer), and six widely used smartphone cameras: iPhone 14 Pro, Samsung S23 Ultra, Google Pixel 7, Xiaomi 13, Huawei P60, and OnePlus 11. All tests were performed under standardized D65 illumination at three light levels (100, 500, and 10,000 lux), with automatic white balance and exposure enabled to reflect typical user conditions. For each (printer, camera) combination, we printed 100 identical SSMC snippets (20 dots, payload “TEST”), captured 10 images per lighting condition, and computed symbol accuracy as the percentage of dots whose quantized RGB values matched the ground-truth palette $\mathcal{C} = \{0,85,170,255\}$. Color fidelity was quantified via the CIELAB ΔE_{00}^{*} metric using an X-Rite i1Pro 3 spectrophotometer, with ground-truth RGB references derived from calibrated screen measurements (sRGB, 100% brightness) via an X-Rite ColorChecker Classic, ensuring traceability to international color standards (ISO 12647-2).

S11. Encoding Complexity Analysis

The Penrose tiling generation via iterative subdivision exhibits time and space complexity of $O(4^{d})$, where d denotes the subdivision depth (Section 2.1); for $d = 3$, this yields 640 triangles and 320 centroids, sufficient for the 200-dot SSMC configuration evaluated in Section 3. To assess scalability, we benchmarked encoding latency for payloads ranging from 20 to 250 bytes (200 to 2,500 dots) on a Dell XPS 15 (Intel Core i7-13700H, 32 GB RAM), observing encoding times of 8.2 ms to 14.7 ms in MATLAB, 6.1 ms to 11.2 ms in Python with Numba JIT acceleration, and 1.8 ms to 2.3 ms on an NVIDIA

RTX 4070 GPU (with performance plateauing beyond 1,000 dots due to I/O overhead). Critically, tiling generation is amortizable: the seed tiling (depth 1, 20 triangles) can be precomputed and stored in 1.2 KB of memory, enabling runtime expansion via vectorized midpoint bisection, which reduces the per-dot encoding cost to constant $O(1)$ for $N > 100$ dots (Fig. S3). For mobile deployment, a lightweight C++ encoder (12 KB binary) achieves 9.3 ms for 2,500 dots on a Snapdragon 8 Gen 2 platform. Collectively, these results confirm that encoding complexity poses no practical barrier to real-world adoption, even high-capacity SSMC instances (250 bytes) encode in under 15 ms on consumer-grade hardware.

S12. Security Analysis

SSMC's security derives from the computational hardness of seed recovery, not combinatorial size alone. The seed space comprises three parameters: (i) subdivision depth $d \in \{2,3,4,5\}$, (ii) rotational offset $\theta \in [0,2\pi)$ discretized to 0.1° (7,200 values), and (iii) radial scaling $s \in \{0.8,0.9,1.0,1.1,1.2\}$, yielding a total space of $4 \times 7{,}200 \times 5 = 144{,}000$ configurations. The Shannon entropy is thus $H = \log_2(144{,}000) = 17.1$ bits, insufficient for cryptographic security but adequate for practical anti-counterfeiting when combined with physical copying failure capacity.

We evaluate two attack models:

1. Brute-force: Enumerate all 144,000 seeds, regenerate tiling, and compute MSE against observed dot positions. At 10 ms/seed (Section 2.8), this requires 24 minutes on a single CPU, feasible for targeted attacks but prohibitive at scale (e.g., 1M codes = 34 years).

2. Algorithmic reconstruction: Use geometric hashing [29] to match dot triplets to Penrose templates. In 10,000 simulated attacks (AWS EC2 c6i.4xlarge), success rate was 0% when occlusion >15% or noise >10σ (Table S4, Appendices), as aperiodicity prevents alignment.

Theorem 1 (Practical Security Bound): *For an SSMC instance with N ⩾100dots, occlusion >15%, and sensor noise σ >10, the probability of successful algorithmic reconstruction is <*10^{-4} *in <1 hour on commodity hardware.* Proof: See Appendices Section S2.1.

S13. Color QR Benchmarking Protocol

We reproduced Yang et al.'s color QR [11] using their open-source code (GitHub: ouyangzhibo), with parameters: 8-level RGB (0, 36, 73, 109, 146, 182, 219, 255), V10 grid, 30% Reed–Solomon ECC. Tests were conducted under identical conditions as SSMC (Section 2.6):

1. Chromatic aberration: Simulated via radial distortion (OpenCV cv2.undistort, k1 = 0.1 to 1.5),
2. Illumination: D65 (100–10,000 lux) and A (2856K) via LED panel,
3. Channel crosstalk: Added via matrix $M = \begin{bmatrix} 1 & c & c \\ c & 1 & c \\ c & c & 1 \end{bmatrix}$, $c = 0 - 0.3$.

Decode success was measured using Yang et al.'s CNN decoder (ResNet-18, 224×224 input) [11].

S14. ECC Trade-off Analysis

Although the (3,1) repetition code exhibits a low code rate ($R = 1/3$), its selection is

grounded in SSMC's physical error model, wherein occlusion typically manifests as spatially contiguous dot clusters (burst errors) rather than isolated symbol corruption. To quantify this trade-off, we evaluated three error-correcting schemes, (3,1) repetition, Reed–Solomon RS(255,223) ($R = 0.874$), and LDPC(1024,768) ($R = 0.750$), under two error models: (i) random occlusion (uniform masking, 0–50%) and (ii) burst occlusion (3×3 block masking, 0–30 blocks, simulating scratches or sticker damage). Under random occlusion, RS achieves superior decode success (99.8% vs. 95.2% at 40% occlusion), leveraging its high code rate and algebraic correction capability; however, under burst occlusion, repetition dominates (92.1% vs. 63.4% success at 10 blocks), as RS's syndrome-based decoding fails catastrophically when errors cluster, whereas majority voting in repetition tolerates localized corruption. LDPC exhibits intermediate robustness but incurs 3.2× higher decode latency (168.4 ms vs. 52.3 ms) and 4.2× greater energy consumption (22.1 mJ vs. 5.3 mJ). Crucially, when evaluated by system-level efficiency, defined as the product of success rate, inverse latency, and inverse energy, repetition outperforms RS by 2.8× and LDPC by 4.1× in burst scenarios (Fig. S4, Appendices), confirming its suitability for edge-constrained applications such as IoT sensors, where speed and power efficiency are paramount.

S15. Physical Metrology Protocol

All density calculations adhere to ISO/IEC 15415 (print quality assessment) and ISO/IEC 18004 (QR code specifications), ensuring traceability and reproducibility. For an 800×800-pixel image printed at 300 dpi, the physical dimensions are computed as $\frac{800}{300} \times 25.4 = 67.73mm \times 67.73mm$, yielding a total print area of 4,588 mm^2, while the

module/dot pitch is $\frac{25.4}{300} = \mathbf{84.67}$μm; critically, the effective area is defined as the full image footprint, with no bounding-box approximation. Baseline comparisons encompass QR Codes (Versions 1–40, 21–177 modules, H-level ECC, ISO/IEC 18004:2015), Data Matrix (ECC200, 10×10 to 144×144 modules, ISO/IEC 16022:2006), and Aztec codes (Compact: 15×15 to 31×31; Full: 27×27 to 151×151, ISO/IEC 24778:2008), with payload capacities sourced directly from the official standard tables (ISO/IEC 18004:2015, Table 3; ISO/IEC 16022:2006, Table 4; ISO/IEC 24778:2008, Table S19). SSMC employs 200 dots at 6 bits/dot (1,200 bits raw), applies 3× symbol repetition for ECC (400 bits effective payload), and reserves 16 bits for CRC-16, yielding a net payload of 50 bytes; all physical measurements were validated using an X-Rite i1Pro 3 spectrophotometer, confirming area accuracy within ±0.5%.

S16. Geometric Registration Protocol

While SSMC eliminates finder patterns, geometric registration remains essential under perspective distortion and cropping. Our pipeline uses a coarse-to-fine template-matching approach on dot centroids:

1. Coarse alignment: Detect 3+ dots via connected-components (min area = 9 px^2), compute centroid positions, and solve for similarity transform (scale, rotation, translation) via RANSAC (max 100 iterations, inlier threshold = 3 px).
2. Fine refinement: Iteratively project the full Penrose template onto the image, compute MSE between observed and expected dot positions, and optimize via Levenberg–Marquardt (max 20 iterations, tolerance = 0.5 px).
3. Failure detection: Abort if < 3 inliers (coarse) or MSE > 5 px^2 (fine).

This protocol requires no feature detection (e.g., contours, corners), only centroid localization, preserving SSMC's simplicity. It succeeds for scale distortions ≤±20%, rotation ≤±45°, and cropping ≥70% of original area (Fig. S5, Appendices).

Template alignment requires estimating the similarity transform $\mathbf{T} = [s\boldsymbol{\mathcal{R}}|\mathbf{t}]$ mapping template centroids $\{\mathbf{p}_j\}$ to observed dots $\{\mathbf{q}_j\}$. This is solved via:

Step 1: Coarse Alignment (RANSAC)

(a) Detect centroids (min area = 9 px^2),

(b) Sample triplets to compute candidate $\mathbf{T}$,

(c) Select best $\mathbf{T}$ (≥3 inliers, threshold = 3 px).

Step 2: Fine Refinement (Levenberg–Marquardt)

(a) Minimize $C(\mathbf{T}) = \sum_i \|\mathbf{T}\mathbf{p}_i - \mathbf{q}_i\|^2$,

(b) Converge if $\|\Delta\mathbf{T}\|$<0.5 px or 20 iterations.

Step 3: Dynamic Scale Adaptation

(a) If RANSAC fails (<3 inliers), retry at 3 scales (0.8×, 1.0×, 1.2×),

(b) Increases success under cropping (from 83% → 89% at 65% area).

Failure Thresholds (empirical):

(a) Perspective distortion >45°: template mismatch,

(b) Cropping <65%: <3 inliers,

(c) Pose variation (pitch/yaw >30°): severe foreshortening.

Full distortion analysis is in Table S13 and Figure S10.

S17. Cross-Device Color Stability

To evaluate robustness under real-world color variation, we tested SSMC on four

printers (Epson EcoTank L3210, HP LaserJet Pro M404dn, Canon Selphy CP1500, Zebra ZT411) and six smartphones (iPhone 14 Pro, Samsung S23 Ultra, Google Pixel 7, Xiaomi 13, Huawei P60, OnePlus 11), under D65 (100, 500, 10,000 lux) and A (2856K) illumination. For each (printer, camera) pair:

1. Print 100 identical SSMC snippets (20 dots, payload "TEST"),
2. Capture 10 images per condition,
3. Compute symbol accuracy as % of dots where quantized RGB matches ground truth,
4. Measure color deviation via CIELAB ΔE_{00}^{*} (X-Rite i1Pro 3 spectrophotometer).

Color Confusion Matrix: We constructed a 4×4 matrix (R, G, B levels) showing misclassification rates under worst-case conditions (Zebra thermal, Xiaomi phone, 100 lux, A light).

Channel Decision Thresholds: Empirically derived from 10,000 samples: R ∈ [0,42) → 0, [42,127) → 85, [127,212) → 170, [212,255] → 255; G and B follow identical logic.

Bit-Error-Rate (BER) Curves: BER = 1 − success rate, plotted against ΔE_{00}^{*} thresholds (0–15), with 95% confidence intervals.

S18. FEC Comparison Protocol

To ensure equitable evaluation, we fixed three parameters across all codes:

1. Physical area: 4,588 mm² (800×800 px, 300 dpi),
2. Effective payload: 50 bytes (after ECC and 16-bit CRC),
3. Occlusion model: 40% block occlusion (10×10 px blocks, 500 trials).

Baseline implementations:

1. SSMC: (3,1) repetition, 200 dots, 600 bits raw → 200 bits effective,

2. QR-RS: V10-H, RS (68,50) (ISO/IEC 18004), 50B net,
3. QR-LDPC: V10, LDPC (85,50) (DVB-S2 standard), open-source decoder,
4. QR-Polar: V10, Polar (75,50) (5G NR standard), open-source decoder.

All decoders run on Dell XPS 15 (i7-13700H), with latency/energy measured via Intel VTune. Success rate = % of trials with perfect payload recovery.

S19. Geometric Registration Pipeline

Template regeneration requires estimating the similarity transform $\mathbf{T} = \begin{bmatrix} s\cos\theta & -s\sin\theta & t_x \\ s\sin\theta & s\cos\theta & t_y \end{bmatrix}$ that maps template dot positions $\{\mathbf{p}_j\}$ to observed centroids $\{\mathbf{q}_i\}$. This is solved via a coarse-to-fine approach:

Step 1: Coarse Alignment (RANSAC)

1. Detect connected components (min area = 9 px²) to extract dot centroids $\{\mathbf{q}_i\}$.
2. For each triplet $(\mathbf{q}_a, \mathbf{q}_b, \mathbf{q}_c)$, compute similarity transform $\mathbf{T}_{abc}$ that minimizes $\sum_i \|\mathbf{T}_{abc}\mathbf{p}_i - \mathbf{q}_i\|^2$ for all N dots.
3. RANSAC (max 100 iterations, inlier threshold = 3 px) selects the best $\mathbf{T}$ with maximum inliers.

Step 2: Fine Refinement (Levenberg–Marquardt)

1. Initialize with $\mathbf{T}_{\text{coarse}}$.
2. Minimize cost function $C(\mathbf{T}) = \sum_i \|\mathbf{T}\mathbf{p}_i - \mathbf{q}_i\|^2$ using Levenberg – Marquardt (max 20 iterations, tolerance = 0.5 px).

Convergence Conditions:

1. RANSAC converges if ⩾3 inliers (required for similarity transform).

2. L-M converges if $\|\Delta\mathbf{T}\| < 0.5$ px or iteration count > 20.

Failure Modes:

1. <3 inliers: Abort (e.g., severe occlusion or blur).
2. MSE > 5 px^2: Abort (e.g., perspective distortion >45°).

This pipeline requires no feature detection, only centroid localization, preserving SSMC's simplicity. Failure rate is 4.2% under realistic distortions (Table S10, Appendices).

S20. Security Threat and Deployment Model

SSMC is not cryptographically secure; no physical encoding can be, without secret keying. Instead, it offers structural hardness: a combination of physical copying failure (ink variation), computational cost (seed recovery), and error correction (3× repetition) that makes mass counterfeiting economically infeasible. This aligns with ISO/IEC 30145-2's definition of practical anti-counterfeiting: "a system whose replication cost exceeds the attacker's expected return." We define four attack tiers:

T1: Copying/Rephotographing: Attacker captures SSMC via camera and reprints.

T2: Seed Recovery: Attacker infers Penrose seed (depth, rotation, scale) from observed dots.

T3: Adversarial Color Perturbation: Attacker modifies RGB values to flip bits (e.g., 85 → 170).

T4: Full Fabrication: Attacker generates new SSMC with valid payload (requires seed + payload key).

Defenses:

1. T1: Defeated by physical copying failure (ink/paper variation, Fig. S8);

2. T2: Requires >24 minutes computation per instance (Section 2.9);
3. T3: 3× ECC corrects 1 symbol error/triplet;
4. T4: Requires seed (144,000 combinations) + payload key (2^{128} if AES used).

Forgery Cost: Minimum cost for 1,000 forgeries is $250,000 (Table S11, Appendices), exceeding typical counterfeiting ROI [18], [30], [31].

SSMC operates under a three-tier deployment model, each with explicit threat assumptions:

Tier 1: Open Verification (e.g., public art, product info)

(1) Assumption: No authentication required; anyone may decode.
(2) Parameters: Only public scaffold depth d (e.g., $d = 3$) is used.
(3) Threat model: None — data is public.

Tier 2: Authenticated Decoding (e.g., supply chain, pharma)

(1) Assumption: Decoder is trusted (e.g., customs, retailer); spoofing by untrusted parties must be prevented.
(2) Parameters: Public d + private seed (θ, σ)) shared via secure channel (PKI) or hash-assisted search (Suppl. S22).
(3) Threat model: Active forgery (T1–T4, Suppl. S19); minimum forgery cost = $250K/1,000 units (Table S11).

Tier 3: Secure Payload (e.g., IoT firmware)

(1) Assumption: Both scaffold and payload are confidential.
(2) Parameters: Private d, (θ, σ), and payload key (AES-128) pre-shared.
(3) Threat model: Full adversarial control (side-channel, physical access).

Crucially, SSMC requires no embedded metadata or geometric “key” in the image, all parameters are either public or managed out-of-band. This preserves aesthetic neutrality and prevents visual leakage.

S21. Point Set Analysis

The SSMC scaffold is generated via midpoint subdivision of a pentagonal star, a deterministic process yielding 640 triangles after 3 iterations. While distinct from cut-and-project Penrose tilings [32], this point set exhibits three critical properties:

(i) Non-periodicity: No translational vector $\mathbf{v} \neq \mathbf{0}$ satisfies $\mathcal{P} + \mathbf{v} = \mathcal{P}$, proven via contradiction (Appendices Theorem 2). The centroid set $\mathcal{C} = \{\mathbf{c}_j\}$ contains irrational ratios (e.g., $\|\mathbf{c}_1 - \mathbf{c}_2\| / \|\mathbf{c}_2 - \mathbf{c}_3\| = (1 + \sqrt{5})/2$), precluding lattice formation.

(ii) 5-fold rotational symmetry: CC is invariant under rotation by $2\pi/5$ about the origin (Lemma 1, Appendices S2.2). This enables intrinsic rotation invariance without alignment markers.

(iii) Sampling uniformity: Local density $\rho(\mathbf{x}) = \frac{1}{\pi r^2} |\mathcal{C} \cap B_r(\mathbf{x})|$ satisfies $|\rho(\mathbf{x}) - \rho_0| < \mathbf{0.12}$ for $r = 20$ px (Fig. S9), ensuring fixed-capacity encoding (200 dots) across the image.

These properties collectively provide structural uniqueness and geometric robustness, without requiring true quasicrystallinity.

S22. Metrology and Statistical Protocol

1. Photometric Calibration:
   (i) Illuminance (lux) and luminance (cd/m²) measured with X-Rite i1Pro 3 spectrophotometer (calibrated to NIST SRM 1920, ±2% uncertainty),

(ii) Exposure time fixed at 1/60 s; metric = bits / (mean luminance × area × time),

(iii) Units: bits/($cd \cdot m^{-2} \cdot s$) (SI-traceable; 1 lux = 1 $cd \cdot sr/m^2$, sr ≈ 1 for smartphone FOV).

2. Energy Measurement:

(i) CPU energy (mJ) via Intel RAPL (Running Average Power Limit) for integrated GPU/CPU,

(ii) Cross-validated with JouleScope hardware power monitor (±1% accuracy),

(iii) 50 trials per condition, median reported (non-normal distribution).

3. Statistical Methodology:

(i) Confidence intervals: 95% CI via 1,000 bootstrap resamples (non-parametric),

(ii) Normality: Shapiro–Wilk test ($p > 0.05$ for all metrics except BER),

(iii) Homoscedasticity: Levene's test ($p > 0.1$ for latency, energy, density),

(iv) Hypothesis testing: Two-tailed t-test ($\alpha = 0.001$) for normal metrics; Mann–Whitney U for BER.

4. Sample Independence:

(ii) All trials used independent image captures (different print instances, lighting setups),

(iii) No reuse of images across conditions (confirmed via SHA-256 hash).

S23. Seed Distribution Protocol

SSMC does not embed any geometric "key" or metadata in the printed image, doing

so would compromise structural uniqueness and visual integrity. Instead, scaffold parameters follow a public–private split:

(a) Public: Subdivision depth d (domain-specific, Appendices),

(b) Private: Rotation θ and scale σ (managed via PKI or hash-assisted search, Suppl. S19, Tier 2).

The scaffold is generated from a seed tuple $\mathbf{s} = (d, \theta, \sigma)$, where $d \in \{2,3,4,5\}$ is subdivision depth, $\theta \in \{0.0°, 0.1°, \ldots, 359.9°\}$ is rotation (7,200 values), $and\, \sigma \in \{0.8, 0.9, 1.0, 1.1, 1.2\}$ is radial scale. To balance openness and security:

(a) Public parameters: d is fixed per use case (e.g., $d = 3$ for industrial labeling) and published in application-specific standards (Appendices). No secrecy is required — it governs only scaffold complexity, not identity.

(b) Private seed components: θ and σ are never embedded in the image. Instead, a 12-bit truncated hash $h_{12} = Trunc_{12}(SHA3 - 256(\mathbf{s}))$ is appended to the payload (extending CRC-16 to 28 bits total). During decoding, the receiver:

   (1) Extracts candidate payloads (Suppl. S15),

   (2) Computes MSE for all 36,000 (θ, σ) combinations,

   (3) Selects the seed with minimum MSE and matching h12h12.

This "hash-aided brute-force" requires only 87 µs per seed (Section 2.6), completing in <3.2 s total, accelerated to <100 ms via GPU or precomputed lookup (GPU version). For high-security applications (e.g., pharma), θ and σ are distributed via PKI (e.g., X.509 certificates), eliminating search entirely.

S24. Color Calibration Sensitivity and Mitigation Strategies

SSMC’s 6-bit encoding (RGB ∈ {0, 85, 170, 255}) is sensitive to device-specific color reproduction. To quantify this, we tested under four conditions:

(1) Illuminance: 100, 500, 10,000 lux (D65 and A light),

(2) White balance: Auto, Daylight, Tungsten,

(3) Printer variation: Epson EcoTank, HP LaserJet, Canon Selphy, Zebra thermal,

(4) Camera variation: iPhone 14 Pro, Samsung S23 Ultra, Google Pixel 7.

Failure Modes:

(1) F1: Quantization error — RGB value shifts beyond decision boundary (e.g., 85 → 170), causing bit flip.

(2) F2: Channel crosstalk — R/G/B values bleed due to ink/paper interaction.

(3) F3: Low-light noise — SNR < 20 dB causes centroid drift.

Mitigation Strategies:

(1) M1: Coarse quantization — 4-level palette tolerates $\Delta E^*_{00} \leqslant$ 5.0 (Table S8).

(2) M2: Multi-exposure capture — Capture 3 images (EV −1, 0, +1); select best per dot. Restores 92% success at ΔE^*_{00} = 12.0.

(3) M3: Adaptive thresholds — Dynamically adjust R=42–127 → 85 based on local histogram (mean ± 2σ). Reduces F1 by 68%.

All tests used X-Rite i1Pro 3 spectrophotometer for ground-truth CIELAB values. Success rate = % of dots where quantized RGB matches ground truth.

S25. Comparison with Representative Color-Based QR Variants

We compare SSMC against four representative color-based QR approaches from recent literature under strictly matched conditions:

1. Physical area: 4,588 mm² (800×800 px @ 300 dpi),
2. Effective payload: 50 bytes (after error correction and 16-bit CRC),
3. Occlusion: 40% random block occlusion (10×10 px blocks),
4. Capture: iPhone 14 Pro, D65 illumination, X-Rite i1Pro 3 calibrated.

The baselines are:

(a) Halftone Multifunctional Color QR: Embeds grayscale images via halftone dithering in QR modules; uses standard RS (68,50) [33],

(b) Visual Cryptography Color QR: Encodes secret color images by splitting RGB channels across two QR codes; requires perfect alignment [34],

(c) Style-Transfer QR: Uses neural style transfer to embed artistic textures into QR modules; relies on module boundary preservation and RS decoding [35],

(d) High-Capacity Colorful QR: Stacks three monochrome QR codes in R, G, B channels; decodes independently per channel [36].

S26. Advanced ECC Integration Pathways

While the baseline SSMC uses a (3,1) repetition code for maximal robustness and alignment-free decoding, the framework is fully compatible with advanced error-correcting codes. The encoding pipeline is modular:

Step 1: Apply any binary ECC to the raw payload (e.g., RS, BCH, LDPC),

Step 2: Map corrected bits to RGB levels (0/85/170/255) via 6-bit grouping,

Step 3: Render on aperiodic scaffold as usual.

Candidate Codes & Trade-offs:

(a) Reed–Solomon (RS): RS (75,50) over GF (2^8) yields 50B payload in 75B codeword

→ +42% capacity vs. repetition. Requires precise centroid detection (fails under >20° perspective).

(b) BCH: BCH (63,45) corrects up to 9bit errors → suitable for moderate occlusion. Moderate complexity (syndrome decoding).

(c) LDPC: Irregular LDPC (600,400) enables soft-decision decoding using RGB confidence (e.g., distance to level boundary). Best for color-noisy environments ($\Delta E^*_{00} > 8$).

Deployment Guidance:

(a) Uncontrolled settings (art, consumer photos): retain (3,1) for alignment-free operation,

(b) Controlled settings (factory, pharma): use RS/LDPC for higher throughput.

Thus, SSMC is not an ECC-specific system, it is a general optical channel that decouples geometry from coding.

S27. Limitations and Mitigation Pathways

No system is without trade-offs. SSMC's 33.3% ECC overhead (vs. QR's 30%) slightly reduces raw capacity for ultra-long payloads. However, this is offset by higher base density (net gain: 2.13×, Table 1). For applications demanding >1 KB payloads, hybrid approaches are viable:

a. Hierarchical encoding: Use SSMC for headers/authenticators, QR for bulk data,

b. Spectral expansion: 4-bit/channel quantization (256 levels) → 12 bits/dot, validated in simulation (2.4× density gain, ±3% accuracy drop).

Second, color calibration remains a challenge for low-end printers. Our tests show that

consumer inkjet devices (Epson EcoTank) maintain >90% decode accuracy at 300 dpi, but grayscale-only printers require a monochrome SSMC variant, currently in development.

S28. Future Directions

Three frontiers await exploration:

a. Multi-spectral SSMC: Extend to UV/IR bands for covert authentication (e.g., banknotes),
b. 3D-Surface SSMC: Generalize tiling to non-developable surfaces (e.g., spheres) via spherical Penrose mappings,
c. Neural Decoders: Replace template regeneration with lightweight CNNs for real-time pose estimation on AR glasses.

Critically, SSMC is not a replacement for QR in all contexts, legacy infrastructure matters. But for *new deployments* where data density, robustness, and aesthetics converge (smart packaging, IoT, metaverse anchors), SSMC offers a post-QR foundation. SSMC provides practical anti-counterfeiting: while not cryptographically unclonable, it exhibits physical copying failure in 25% due to ink/paper variation (Fig. S12), computational seed recovery cost ($250K for 1,000 forgeries), and ECC resilience makes mass counterfeiting economically infeasible, a critical threshold for pharmaceuticals and electronics where ROI < $200K [18], [30], [31]. This is a socioeconomic barrier, not an information-theoretic one, and it is sufficient for many high-value applications (pharma, electronics) where detection need only outpace profit.

S29. Potential Prospects & Validation Roadmap

The demonstrated capabilities of SSMC, high density, robustness, and aesthetic neutrality,

suggest several promising applications. However, these remain hypotheses requiring large-scale validation. Below, we frame each prospect as a testable hypothesis, define success criteria, and outline a third-party validation plan. All validation data will be shared under FAIR principles (Findable, Accessible, Interoperable, Reusable) via Zenodo.

H8.1: Seamless Artistic Integration

Hypothesis: SSMC can be embedded in artworks without perceptual disruption (distraction score ≤ 2.0 on 1–5 scale).

Validation Plan:

1. Method: Double-blind user study (N=200), ISO/IEC 25010 visual quality metrics,
2. Partners: Guilin Museum of Modern Art (GMoMA), Guilin,
3. Timeline: Year 1 (pilot), Year 2 (large-scale).

H8.2: Label-Free Industrial Authentication

Hypothesis: SSMC embedded in product pigments reduces label use by ≥90% while maintaining decode success ≥95%.

Validation Plan:

1. Method: Life-cycle assessment (LCA) per ISO 14040/44, ASTM F3152 (anti-counterfeiting),
2. Partners: Manufacturing Company (Hamadun Group of Companies), Shanghai,
3. Timeline: Year 2 (pilot), Year 3 (industry trial).

H8.3: Curved-Surface Robustness

Hypothesis: SSMC maintains ≥90% decode success on cylinders (radius ≥ 30 mm) under 10,000× handling cycles.

Validation Plan:

1. Method: ISO 18938 (imaging durability), ASTM D3359 (adhesion),
2. Partners: Lijiang AI Institute, Guilin,
3. Timeline: Year 1 (lab), Year 2 (field).

H8.4: Environmental Resilience

Hypothesis: SSMC survives 1,000 hrs UV (Xenon arc, ISO 4892-2) with ≤5% BER increase.

Validation Plan:

1. Method: ISO 4892-2 (UV), ISO 4892-3 (weathering),
2. Partners: SGS, Intertek,
3. Timeline: Year 1 (certification).

Table S21. SSMC Canonical Parameters (Default Configuration)

Parameter	Value	Description
**Seed Tuple**	$s = (d = 3, \theta = 0.0^0, \sigma = 1.0)$	Subdivision depth, rotation, scale
**Scaffold**	Pentagonal star → 3-level midpoint subdivision	Yields 640 triangles → 200 centroids
**Palette**	RGB ∈ {0, 85, 170, 255}	4 levels per channel → 64 colors total
**Dot Radius**	9 px (at 300 DPI)	Circular dot, area = 254 px^2
**ECC**	(3,1) repetition	3 dots per bit; majority vote
**Payload**	50 bytes + 16-bit CRC	Total encoded bits = 600
**Print Size**	800 × 800 px @ 300 DPI	Physical area = 4,588 mm^2

**Capture**	iPhone 14 Pro, D65 light, auto WB	No flash, 1/60 s exposure

Table S22. Heatmap: QR Code (V10-H) Decode Success Rate

**Occlusion \ Noise**	**0σ**	**5σ**	**10σ**	**15σ**	**20σ**	**25σ**	**30σ**
**0%**	100	99.5	97.2	89.3	72.1	48.6	24.3
**10%**	99.8	97.0	85.4	62.7	34.5	12.1	3.2
**20%**	95.2	82.1	54.3	26.8	8.4	1.1	0.0
**30%**	2.1	0.0	0.0	0.0	0.0	0.0	0.0
**40%+**	0.0	0.0	0.0	0.0	0.0	0.0	0.0
