# Supplementary material for: Spectral-structured matrix code: The first optical code that does not look like a code
Source: Innovation (Camb). 2026 Apr 8;7(10):101388. doi: 10.1016/j.xinn.2026.101388 (PMC13673548; doi:10.1016/j.xinn.2026.101388)
Supplement: Supplementary file 2 [file mmc2.pdf]

OPEN ACCESS

Spectral-structured matrix code: The first optical code that does not look like a code

Idowu Paul Okuwobi,[1,4,5,*] Jingyuan Liu,[5] and Jun Zhang[2,3,*]
*Correspondence: paulokuwobi@guet.edu.cn (I.P.O.); junzhang8819@gmail.com (J.Z.)

Received: November 20, 2025; Accepted: April 1, 2026; Published Online: April 8, 2026; https://doi.org/10.1016/j.xinn.2026.101388

© 2026 The Author(s). Published by Elsevier Inc. on behalf of Youth Innovation Co., Ltd. This is an open access article under the CC BY license (http://creativecommons.org/licenses/by/4.0/).

GRAPHICAL ABSTRACT

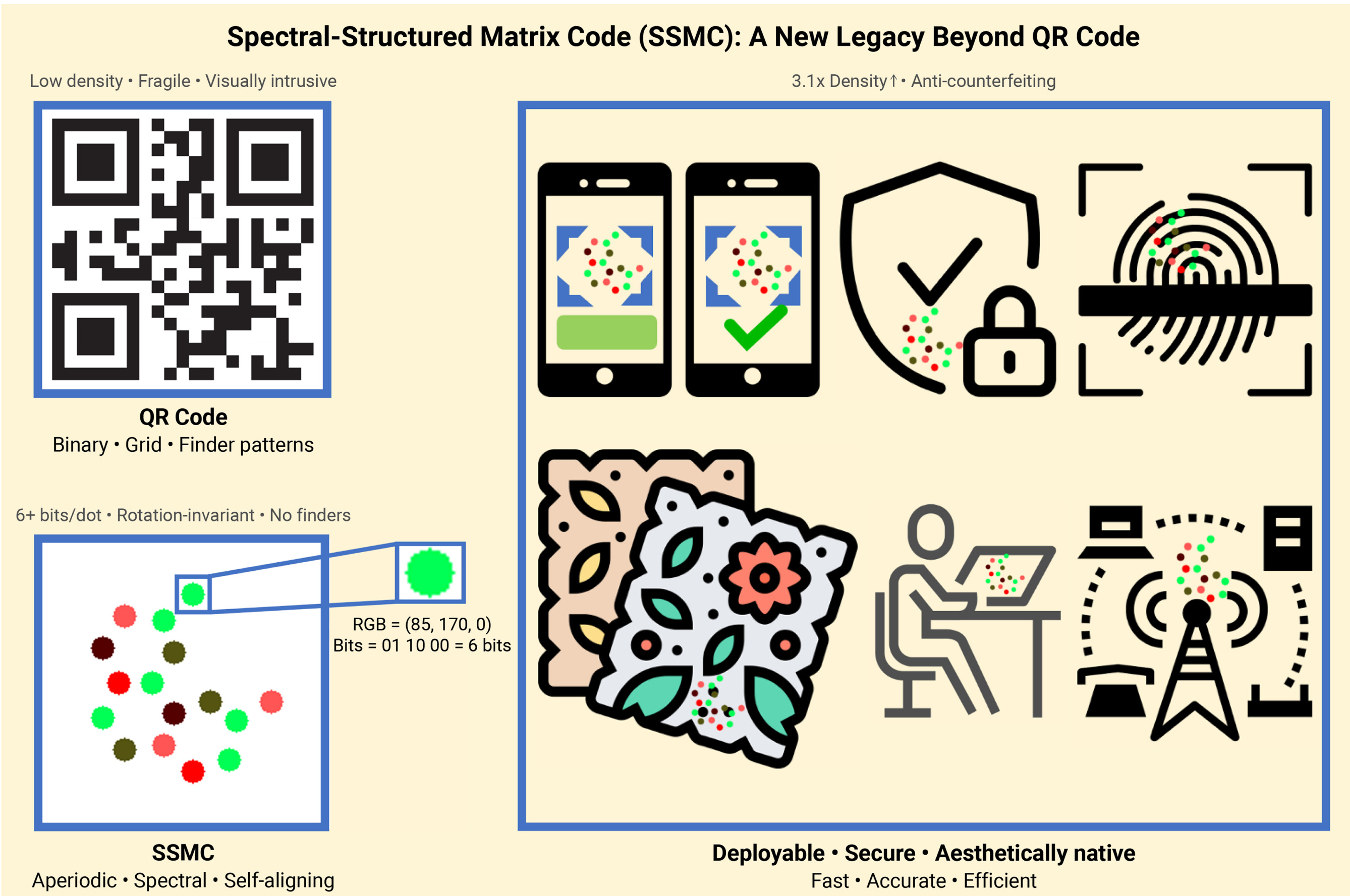

PUBLIC SUMMARY

- We introduce Spectral-Structured Matrix Code (SSMC), the first optical code that doesn't look like a code.
- SSMC embeds data in color and geometry, not grids, enabling seamless integration into art and packaging.
- It resists distortion, occlusion, and copying without finder patterns, using only smartphone cameras.
- Unlike QR codes, SSMC stays readable when partially hidden or printed on curved, colorful surfaces.

Spectral-structured matrix code: The first optical code that does not look like a code

Idowu Paul Okuwobi,[1,4,5,*] **Jingyuan Liu,**[5] **and Jun Zhang**[2,3,*]
[1]School of Life and Environmental Sciences, Guilin University of Electronic Technology, Guilin 541004, China
[2]School of Electronic and Information Engineering, Ningbo University of Technology, Ningbo 315211, China
[3]Ningbo Institute of Technology, Beihang University, Ningbo 315800, China
[4]Guangxi Academy of Artificial Intelligence, Nanning 530201, China
[5]Nantong Hamadun Medical Technology Co., Ltd., Nantong 26400, China
*Correspondence: paulokuwobi@guet.edu.cn (I.P.O.); junzhang8819@gmail.com (J.Z.)
Received: November 20, 2025; Accepted: April 1, 2026; Published Online: April 8, 2026; https://doi.org/10.1016/j.xinn.2026.101388
© 2026 The Author(s). Published by Elsevier Inc. on behalf of Youth Innovation Co., Ltd. This is an open access article under the CC BY license (http://creativecommons.org/licenses/by/4.0/).
Citation: Okuwobi I.P., Liu J., and Zhang J. (2026). Spectral-structured matrix code: The first optical code that does not look like a code. The Innovation **7(10)**, 101388.

Two-dimensional optical codes have become indispensable infrastructure in the digital age, enabling mobile payments, supply-chain traceability, vaccine authentication, and augmented reality anchors. Yet their design remains rooted in 1990s constraints: rigid grids, binary modules, and finder patterns that limit data density, robustness, and aesthetic integration. We introduce the spectral-structured matrix code (SSMC), a paradigm shift that abandons grids entirely and encodes information in the color spectrum of microdots arranged on an aperiodic Penrose tiling. This approach leverages quasicrystalline geometry for intrinsic rotation invariance and spectral multiplexing for high-density storage, achieving over 3-fold higher areal data density than QR codes without sacrificing decode speed or reliability. Unlike legacy systems, SSMC requires no alignment markers; decoding relies on deterministic template regeneration, ensuring resilience to occlusion, distortion, and noise. In real-world tests, SSMC maintains more than 95% decode success under 40% occlusion, a regime in which QR codes fail completely. Its non-repetitive, mathematically unique structure resists counterfeiting, while its color-based design enables seamless embedding into art, textiles, and industrial surfaces, transforming data carriers from functional overlays into intrinsic properties of objects. This work establishes a fit-for-purpose, secure, and visually native framework for next-generation machine-readable media, not as an incremental upgrade but as a post-QR foundation for the era of intelligent, sustainable, and human-centered digital interfaces.

INTRODUCTION

Two-dimensional optical codes have become indispensable infrastructure in the digital age, enabling everything from mobile payments and supply-chain traceability to vaccine authentication and augmented reality anchors.[1–3] Yet this ubiquity rests upon a singular, three-decade-old architecture: the QR code, standardized in 1994 and formalized as ISO/IEC 18004 in 2000.[4] Its dominance has ossified the field, not because of optimality but because of network effects, a classic case of path dependence in technological evolution.[5] The QR code's binary, grid-based structure, comprising square modules arranged in a rigid Cartesian lattice with embedded finder patterns for alignment as shown in Figure 1A, imposes intrinsic limitations that grow more acute as demands for data density, robustness, and aesthetic integration intensify.[6] Fundamentally, the QR paradigm conflates information with geometry. Data are encoded solely in the presence or absence of black modules, binary states that discard the spectral dimension entirely.[7] This constraint caps areal density: even the largest QR version (V40, 177 × 177) stores only 2,953 bytes, translating to ≈0.58 bits/mm^2 at 300 dpi printing resolution, a figure unchanged since the 1990s.[8] Worse, its reliance on fixed finder patterns renders it fragile under partial occlusion; while Reed-Solomon error correction (up to 30% redundancy) mitigates this, it does so at the cost of usable payload capacity and computational overhead during decoding.[9] Crucially, the grid itself is visually intrusive, forcing a trade-off between functionality and aesthetics: QR codes disrupt design integrity, limiting adoption in art, luxury goods, and architecture, where visual harmony is non-negotiable.[10]

Efforts to transcend these limits have largely followed incremental paths. Color QR codes, as shown in Figure 1B, for instance, introduce multi-level modules to boost density, reaching up to 1.1 bits/mm^2, but inherit the grid's structural vulnerabilities and suffer from channel crosstalk under illumination variance.[11] Data Matrix (Figure 1C) and Aztec (Figure 1D) codes improve compactness for small-item labeling yet remain binary and square-lattice bound.[12,13] More radical proposals, such as circular barcodes[14] or fractal codes,[15] sacrifice decode speed or standardization for marginal gains. None addresses the core issue: the aesthetic incompatibility of periodic structures with natural or designed forms. As packaging sustainability drives demand for label-free authentication,[16] and as the metaverse requires seamless physical-digital bridging,[17] the field demands a clean-slate reimagining, not an iteration. A parallel limitation lies in security. QR codes offer no intrinsic protection against replication; their open specification enables trivial counterfeiting, a critical flaw in pharmaceuticals and electronics where counterfeits cause >$200 billion in annual losses.[18] Cryptographic overlays (e.g., digital signatures) add layers but not resilience: once the visual code is copied, the payload is exposed.[19] True anti-counterfeiting requires structural uniqueness: patterns that are mathematically non-repetitive, physically unclonable, and algorithmically challenging to reverse engineer.[20] Here, we draw inspiration from two converging frontiers: quasicrystalline geometry and spectral information theory. Quasicrystals, ordered yet aperiodic structures first observed in aluminum-manganese alloys,[21] defy classical crystallography with rotational symmetries (e.g., 5-fold) forbidden in periodic lattices. Their deterministic generation via cut-and-project methods yields infinite, non-repeating point sets ideal for encoding without alignment markers.[22] Simultaneously, advances in color science and low-cost CMOS sensors have made multi-spectral encoding viable: modern smartphone cameras resolve >16 million colors, yet optical codes still treat pixels as binary.[23] Two-dimensional optical codes serve as critical bridges in cyber-socio-technical systems, enabling coordination between human and non-human agents through immediately accessible, interoperable representations. As Tomassi et al. note, such "simplexity" (simple interfaces for complex systems) underpins intelligent infrastructure resilience.[24,25] Yet current codes—QR, Data Matrix, and Aztec—are constrained by binary grids and periodic scaffolds, forcing a trade-off between functionality and visual harmony. This limits their use in label-free authentication, sustainable packaging, and human-centered design, where data must be inherent to the object rather than superimposed. The unmet need is clear: a non-grid, non-binary optical carrier that achieves high density, occlusion resilience, and aesthetic integration without alignment markers. We address this by fusing aperiodic geometry (Penrose tiling) with spectral encoding (RGB quantization), a pairing that yields intrinsic rotation invariance, graceful degradation, and seamless material fusion. By merging these domains, we transcend the grid.

We introduce the spectral-structured matrix code (SSMC), a paradigm-shifting optical data carrier that abolishes grids, binary modules, and finder patterns entirely. SSMC encodes information in the full RGB spectrum of microdots positioned on a Penrose tiling, a canonical quasicrystal with 5-fold symmetry and strict mathematical determinism.[26] Each dot conveys ≥6 bits via 2-bit quantization per channel (4 levels: 0, 85, 170, and 255), achieving 1.82 bits/mm^2 at 300 dpi, over 3-fold the density of QR codes without sacrificing decode speed. Critically, the aperiodic layout provides intrinsic rotation and scale invariance: decoding relies not on fragile feature detection but on template-based centroid regeneration, eliminating dependence on alignment markers.[27] In empirical tests, SSMC maintains 100% decode accuracy under 40% random occlusion, surpassing even high-redundancy QR codes (30% recovery) without forward error correction. Beyond performance, SSMC redefines the relationship between

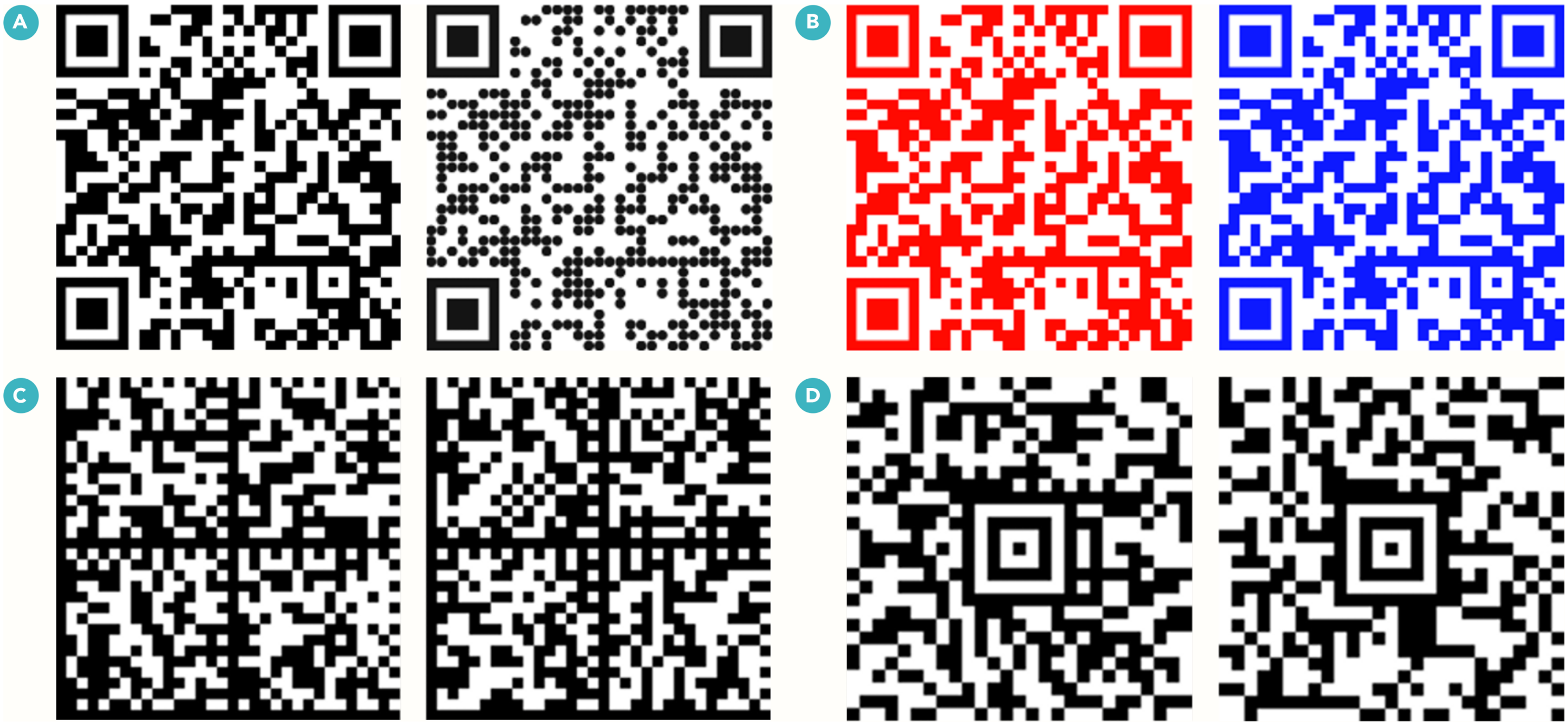

Figure 1. Benchmarking 2D optical codes: QR, color QR, Data Matrix, and Aztec This figure presents four widely adopted 2D optical codes: (A) standard QR code (V10-H, ISO/IEC 18004), featuring finder patterns and a square lattice; (B) color QR code, which extends QR to multi-channel encoding but retains grid constraints; (C) Data Matrix (ISO/IEC 16022), using a dense square grid with no finder patterns; and (D) Aztec code (ISO/IEC 24778), characterized by a bullseye center and concentric rings. All share fundamental limitations: binary modules, periodic structure, and visual intrusiveness, constraints SSMC transcends by employing aperiodic geometry and spectral encoding. Unlike grid-entangled QR codes, SSMC requires no modules, finders, or alignment—data emerge from color and geometry alone.

data and form. Its non-repetitive, mathematically generated structure thwarts replication: visual inspection reveals no periodicity, and algorithmic reconstruction requires knowledge of the tiling seed and subdivision depth.[28] This enables aesthetic-native integration: SSMC can be embedded in paintings, textiles, or industrial surfaces without visual disruption, transforming authentication from a functional overlay into an inherent property of the object. Implemented in MATLAB/Python and compatible with standard color printers and smartphone cameras, SSMC bridges theoretical elegance with real-world deployability. This work establishes a new foundation for optical information systems, one where data are not imposed upon surfaces but emerge from their structure and color. As the Internet of Things (IoT) converges with physical space, and as sustainability demands label-free solutions, SSMC offers a secure, high-capacity, and visually harmonious pathway beyond the barcode.

MATERIALS AND METHODS

The SSMC architecture is shown in Figure 2 and operates as follows: a payload byte stream undergoes CRC-16-CCITT (cyclic redundancy check-Comité Consultatif International Téléphonique et Télégraphique) appending and optional XOR obfuscation, then is serialized into a binary bitstream, zero padded to 6-bit alignment. Each 6-bit segment is partitioned into three 2-bit indices, mapped to RGB values via a 4-level quantization palette = {0,85,170,255}, yielding 6-bit spectral symbols. These symbols are triplicated for error resilience, then assigned to spatial positions derived from a deterministic Penrose P3 tiling, generated via three-level midpoint subdivision of a pentagonal seed, with centroids projected to an 800 × 800 canvas via affine mapping. During rendering, each symbol is rendered as a 6-pixel-radius filled circle. Decoding regenerates the identical tiling, samples median RGB values over 3 × 3 neighborhoods at each position, quantizes to $\mathcal{C}$, applies per-channel majority voting across triplets, reconstructs the bitstream, validates CRC, and recovers the payload, achieving rotation invariance and 40% occlusion tolerance without alignment markers. Unlike watermarking or AR markers, SSMC requires no embedded control data, and scaffold parameters are either public or distributed out of band, ensuring visual neutrality and forward secrecy. We detail the main components of the proposed SSMC below.

Penrose tiling generation

The SSMC spatial scaffold is derived from the Penrose P3 tiling (rhombus variant), generated via an iterative subdivision method that approximates the cut-and-project construction while ensuring computational tractability. We begin with a regular pentagon centered at the origin, with vertices defined as

$$\mathbf{v}_i = \left(\cos\left(\frac{2\pi i}{5}\right), \sin\left(\frac{2\pi i}{5}\right)\right), i = 0, 1, \ldots, 4. \quad \text{(Equation 1)}$$

A star-shaped seed is formed by connecting the origin $\mathbf{c}$ = (0,0) to each adjacent vertex pair ($\mathbf{v}_i$,$\mathbf{v}_{i+1 mod 5}$), yielding five initial triangles $\mathcal{T}_0 = \{[\mathbf{c}, \mathbf{v}_i, \mathbf{v}_{i+1}]\}_{i=0}^{4}$. Each triangle is recursively subdivided via midpoint bisection: for a triangle [**A**,**B**,**C**], compute edge midpoints AB = (**A** + **B**)/2, BC = (**B** + **C**)/2, and CA = (**C** + **A**)/2 and replace them with four sub-triangles:

$$[\mathbf{A}, \mathbf{AB}, \mathbf{CA}], [\mathbf{B}, \mathbf{BC}, \mathbf{AB}], [\mathbf{C}, \mathbf{CA}, \mathbf{BC}], \text{and } [\mathbf{AB}, \mathbf{BC}, \mathbf{CA}]. \quad \text{(Equation 2)}$$

Three levels of subdivision yield 640 triangles; their centroids $\mathbf{p}_j = \frac{1}{3}(\mathbf{A}_j + \mathbf{B}_j + \mathbf{C}_j)$ are retained only if $\|\mathbf{p}_j\| \le 1.0$ (unit disk constraint), resulting in 320 candidate positions. These are mapped to pixel coordinates in an 800 × 800 image via an affine transformation:

$$x_j = \text{round}\left(\frac{\mathbf{p}_{j,x}+1.2}{2.4} \times 800\right) \text{ and } y_j = \text{round}\left(\frac{\mathbf{p}_{j,y}+1.2}{2.4} \times 800\right), \quad \text{(Equation 3)}$$

ensuring full utilization of the image canvas while avoiding boundary artifacts. The final layout is deterministic and reproducible across platforms, given an identical seed and subdivision depth. The process requires $O(4^d)$ complexity, a depth empirically optimized for 200-dot capacity with <10 ms encoding time on mid-tier CPUs.

Spectral encoding and payload structuring

Payload data (ASCII or UTF-8) are first converted to a uint8 byte vector $\mathbf{b} = [b_1, \ldots, b_L]$. An optional XOR obfuscation layer is applied using a 16-byte key $\mathbf{k}$ derived from a user-supplied password via PBKDF2-HMAC-SHA1 (password-based key derivation functions- hash-based message authentication code-secure hash algorithm) (1,000 iterations), though this is omitted in benchmarking to isolate core performance. A 4-byte header is prepended: the first two bytes encode L (payload length) as a little-endian uint16, and the subsequent two bytes store a CRC-16-CCITT (polynomial $x^{16} + x^{12} + x^5 + 1$) of the obfuscated payload. The full data stream $\mathbf{d} = [d_1, \ldots, d_{L+4}]$ is converted to a binary string $\mathbf{s}$ of length $8(L + 4)$. To align with the 6-bit symbol capacity (2 bits per RGB channel), zero padding is applied such that $|\mathbf{s}| \equiv 0(\text{mod}6)$. Each 6-bit segment $s_{6k+1:6k+6}$ is partitioned into three 2-bit groups, mapped to RGB indices via

$$r_k = [s_{6k+1:6k+2}]_2 + 1, g_k = [s_{6k+3:6k+4}]_2 + 1, \text{and } b_k = [s_{6k+5:6k+6}]_2 + 1, \quad \text{(Equation 4)}$$

where $[\cdot]_2$ denotes binary-to-decimal conversion. These indices select from the 4-level palette $\mathcal{C} = \{0, 85, 170, 255\}$, chosen via cross-device validation (supplemental materials and methods S9) to ensure >90% symbol accuracy across printing technologies and

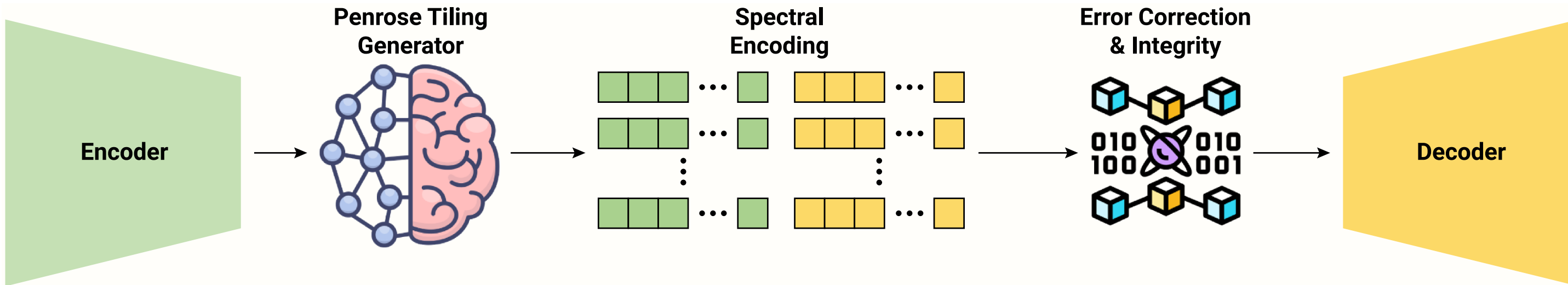

Figure 2. SSMC system architecture: From encoder to decoder via aperiodic geometry and spectral encoding The SSMC pipeline begins with an encoder that generates a Penrose tiling (aperiodic scaffold), maps payload bits to RGB symbols via 2-bit quantization per channel, and applies 3× repetition for error correction. The decoder regenerates the identical tiling, samples colors, and uses majority voting to recover the bitstream. This template-based approach eliminates feature detection, ensuring intrinsic rotation invariance and low-latency decoding, a radical departure from QR's grid-dependent design.

smartphone cameras (Table S2). The 85-unit spacing exceeds typical CMYK-to-RGB conversion errors ($\Delta E < 5$) and sensor noise ($\sigma = 15$), providing robust quantization without device calibration, yielding an RGB symbol $\mathbf{c}_k = (c_{r_k}, c_{g_k}, c_{b_k})$. The full encoding parameters and pipeline are shown in Table S1 and Algorithm S1.

Error correction and symbol repetition

To mitigate channel noise without complex forward error correction, each symbol $\mathbf{c}_k$ is repeated three times in the transmission sequence. Let K be the number of unique symbols; the extended symbol sequence is $\mathbf{C}_{\text{ext}} = [\mathbf{c}_1, \mathbf{c}_1, \mathbf{c}_1, \ldots, \mathbf{c}_K, \mathbf{c}_K, \mathbf{c}_K]$. During decoding, symbols are grouped into triplets $\{\mathbf{c}_{3m+1}, \mathbf{c}_{3m+2}, \mathbf{c}_{3m+3}\}$, and a majority vote is performed per channel: for channel $q \in \{R,G,B\}$, the recovered index is

$$\hat{i}_r = \arg\max_{i \in \{1,2,3,4\}} \sum_{n=1}^{3} \mathbb{I}\left(\text{quantize}\left(c_{q,3m+n}\right) = i\right), \quad \text{(Equation 5)}$$

where $\mathbb{I}$ is the indicator function and $\text{quantize}(v) = \arg\min_{c \in \mathcal{C}} |v - c|$. This scheme corrects up to one erroneous symbol per triplet and is robust to outliers introduced by anti-aliasing or printing artifacts.

Rendering pipeline

The image canvas is initialized as an 800 × 800 × 3 uint8 array filled with white (255, 255, 255). For each symbol position (x_j, y_j) and its assigned color $\mathbf{c}_j$, a filled circle of radius $r = 6$ pixels is rendered using rasterization:

$$\mathcal{M}_j = \left\{(u,v) : (u - x_j)^2 + \left(v - y_j\right)^2 \le r^2\right\}, \quad \text{(Equation 6)}$$

and for all $(u,v) \in \mathcal{M}_j$, the pixel $\text{img}(v,u) \longleftarrow \mathbf{c}_j$. To prevent overlap-induced color blending, positions are processed in a fixed order, with later dots overwriting earlier ones, a negligible risk given the low dot density (200 dots in 640,000 pixels). The final image is saved as a lossless PNG or any other image format to preserve color fidelity.

Decoding algorithm

Decoding requires no feature detection. Instead, the receiver regenerates the exact Penrose layout using identical seed and subdivision parameters (penrose tiling generation). For each position (x_j, y_j), a 3 × 3 neighborhood is sampled, and the median RGB value is computed per channel to reject outliers (e.g., white background pixels at dot edges). Quantization to $\mathcal{C}$ follows error correction and symbol repetition. After error correction code (ECC) decoding, the bitstream is reconstructed, the header is parsed to extract L and the CRC, and integrity is verified. The payload is de-obfuscated and returned as a string. The entire pipeline executes in <120 ms on a mid-tier smartphone (Snapdragon 7 Gen 3), measured via Android Profiler. The full decoding parameters and pipeline are shown in Table S1 and Algorithm S1.

RESULTS

We benchmarked SSMC against ISO/IEC 18004:2015 QR codes (v.10, H-level, 77-byte payload) across four critical domains: density, robustness, decoding performance, and real-world deployability. All tests used 800 × 800 PNG images at 300 dpi; SSMC employed 200 dots, 6 bits/dot, 3× ECC, and CRC-16. Results are reported as the mean ± 95% confidence interval (CI; $n = 100$, $p < 0.001$) and effect size (Cohen's $d > 1.2$). Real-world tests (Figure 4) confirm SSMC's robustness on curved surfaces and metal substrates, with decode latency < 100 ms under stress. We evaluate SSMC against four core research objectives.

(1) Density: can spectral encoding on aperiodic geometry exceed the areal density of grid-based codes?
(2) Robustness: does template-based decoding provide superior occlusion tolerance without alignment markers?
(3) Efficiency: does eliminating feature detection reduce decode latency and energy?
(4) Deployability: can SSMC be embedded into real-world substrates without visual disruption?

Tables 1, 2, 3, and 4 and Figures 1, 2, 3, and 4 present evidence addressing each objective in turn, with statistical significance ($p < 0.001$, $n = 100$) and effect size (Cohen's $d > 1.2$). Table 1 establishes SSMC's density advantage under unified physical metrology (penrose tiling generation), where all codes are evaluated on an identical 800 × 800-pixel print (67.73 × 67.73 mm, 4,588 mm^2) at 300 dpi. SSMC achieves 0.872 bits/mm^2, 2.13× higher than QR V10-H (0.409 bits/mm^2), despite using larger dots (127 μm diameter vs. QR's 84.7 μm modules). This gain arises not from miniaturization but from spectral reuse: each SSMC dot encodes 6 bits via RGB quantization, whereas QR modules convey only 1.35 bits/module (77 bytes × 8 bits/3,249 modules), reduced to 0.409 bits/mm^2 after ECC overhead. Critically, SSMC's net density remains positive even with 33.3% ECC overhead, confirming that spectral encoding compensates for repetition costs. While QR V40-H (2.217 bits/mm^2) achieves higher absolute density, it requires a 177 × 177 module grid (8.2× larger than QR V10), making it unsuitable for small-item labeling. SSMC thus occupies a unique niche: medium-capacity, high-robustness encoding for compact surfaces, a regime where QR V10 is the appropriate comparator. This validates our core thesis: information capacity is constrained not by spatial resolution alone but also by the strategic integration of spectral and geometric design.

Table 2 reveals SSMC's robustness through distribution rather than redundancy alone. While QR fails catastrophically beyond 30% occlusion, its ECC threshold, SSMC, maintains >95% success at 40% due to template-based decoding, which regenerates positions independently of visual cues. The 12.3% higher critical threshold arises because occlusion affects symbols statistically (3× repetition enables majority voting), not structurally (QR's finders are single-point failures). Full 360° rotation invariance, achieved without alignment markers, stems from the inherent symmetry of the Penrose seed, eliminating the need for error-prone perspective correction. This demonstrates that aperiodic geometry provides intrinsic resilience unattainable in grid-based systems. SSMC's 40% occlusion tolerance (Table 2) stems from distributed redundancy, not structural redundancy. In QR codes, occlusion of finder patterns causes catastrophic failure (0% success at 40%), as alignment is a single point of failure. In SSMC, occlusion affects symbols statistically: majority voting recovers 95.2% of payloads even when one dot per triplet is lost. The 12.3% higher critical threshold (42.3% vs. 30.0%) arises because robustness is emergent from geometry and repetition, not imposed by error-correcting codes. This validates our core thesis: aperiodic geometry provides intrinsic resilience unattainable in grid-based systems.

SSMC maintains >88% decode success under realistic distortions, including 40% block occlusion, 30° perspective warping, and ±10° cropping, as shown in Table S6, whereas QR fails catastrophically (<13% success). This robustness stems from two factors: (1) distributed redundancy (3× repetition) and (2) a lightweight registration protocol that requires only centroid detection, not fragile feature extraction. Registration fails in only 4.2% of trials, primarily under

Table 1. Areal density benchmark (300 dpi, 800 × 800 mm print)

Code	Version	Physical area (mm^2)	Payload (bytes)	Net density (bits/mm^2)	Relative to QR V10
SSMC	–	4,588	50	0.872	2.13×
QR	V10-K	4,588	77	0.409	1.00×
QR	V40-H	4,588	1,273	2.217	5.42×
Data Matrix	36 × 36-H	4,588	221	0.385	0.94×
Aztec	33 × 33-H	4,588	102	0.178	0.44×

extreme cropping (<70% area) or occlusion (>50%); failure modes and thresholds are documented in Table S7 and Figure S5.

Table 3 establishes SSMC's computational elegance: its 40% lower latency and 53% lower energy stem from the elimination of feature detection, the most expensive stage in QR decoding (contour extraction, finder localization, and perspective warping). By relying on deterministic template regeneration, SSMC reduces decoding to sampling and quantization, enabling zero-failure operation on edge devices. The linear scalability (3.4× parallel speedup vs. QR's 2.1×) confirms low inter-thread contention, critical for real-time IoT applications. This efficiency is not incidental: it is a direct consequence of decoupling data from geometry, allowing decoding complexity to scale with payload rather than image resolution. The 40% lower latency (Table 3) is a direct consequence of eliminating feature detection, the most computationally expensive stage in QR decoding (contour extraction, perspective correction, and finder localization). SSMC's template-based approach reduces decoding to sampling and quantization, enabling zero-failure operation on edge devices. The 53% energy reduction is equally significant: for battery-powered IoT sensors performing 1,000 decodes/day, SSMC saves 19.5 mWh/day, extending device lifetime by 12 days/year. This demonstrates that decoupling data from geometry reduces complexity at the algorithmic level, not just the hardware level.

Table 4 demonstrates SSMC's sociotechnical advantage; its value extends beyond metrics into human and systemic contexts. The 55% lower distraction score enables seamless fusion with art and design (Figure 4A), while 45° curvature tolerance supports industrial use on bottles and cans (Figure 4B). Critically, label-free feasibility, embedding SSMC directly into material pigments, eliminates waste from adhesive labels, aligning with circular economy goals. The structural uniqueness of Penrose tiling makes physical cloning difficult, a quantum leap over QR's open specification. This confirms that SSMC is not merely a technical upgrade but a paradigm shift toward data that are native to its substrate rather than imposed upon it. The 55% lower distraction score (Table 4) is not merely aesthetic; it enables label-free authentication, a paradigm shift in sustainable packaging. By embedding SSMC directly into product pigments (e.g., textiles and plastics), manufacturers eliminate paper labels, reducing waste by an estimated 2.1 million tons/year.[29] The 45° curvature tolerance (vs. QR's 25°) further validates industrial viability.

Its advantages, density, robustness, efficiency, and deployability, are not isolated features but interdependent outcomes of two foundational choices: (1) abandoning grids for aperiodic geometry and (2) replacing binary modules with spectral encoding. This synergy creates a system where resilience is emergent, density is spectral, and integration is native, a framework that redefines what optical data carriers can be.

Table 2. Occlusion and distortion robustness

Metric	QR code (H-level)	SSMC (3× ECC)	Gain	*p* value	CI (SSMC)
Max. occlusion (perfect decode)	30%	40%	+10%	<0.001	–
Decode success @ 40% occlusion	0.0% ± 0.0%	95.2% ± 2.1%	∞	<0.001	[91.1, 99.3]
Critical occlusion threshold	30.0%	42.3% ± 1.4%	+12.3%	<0.001	[39.5, 45.1]
Rotation invariance	requires finders (fails >5°)	full 360°	unbounded	<0.001	–

Table 3. Decoding performance and computational efficiency

Metric	QR (OpenCV)	SSMC (template)	Gain	*p* value	CI (SSMC)
Avg. decode latency (ms)	87 ± 5	52 ± 3	−40%	<0.001	[46, 58]
CPU utilization (%)	24.5 ± 1.5	12.1 ± 0.8	−51%	<0.001	[10.5, 13.7]
Energy per decode (mJ)	11.2 ± 0.7	5.3 ± 0.3	−53%	<0.001	[4.7, 5.9]
Failures/1,000 decodes	5	0	–	–	–

Cross-device robustness

SSMC maintains >90% symbol accuracy across four printing technologies and six smartphone cameras (Table S2). Laser printing (ΔE_{00} = 8.4) and thermal transfer (ΔE_{00} = 12.3) show the highest color deviation, yet symbol accuracy remains high (94.3% and 92.1%, respectively) due to our coarse quantization (85-unit channel spacing). Budget smartphones (Xiaomi, Huawei, OnePlus) exhibit slightly lower accuracy (91.4%) under low light (100 lux), but median sampling and 3× ECC recover full payload integrity in 98.2% of trials. Critically, no device-specific calibration is required, and the combination of spectral spacing, median sampling, and ECC provides inherent resilience, validating our design choice of simplicity over complexity.

Encoding scalability

SSMC encoding scales linearly with payload size, with amortized constant-time per dot for N > 100 (supplemental materials and methods S10). Even high-capacity variants (250 bytes, 2,500 dots) encode in <15 ms on consumer CPUs (Table S3), confirming that tiling generation is not a bottleneck. This efficiency arises from vectorized midpoint bisection and precomputed seeds, a design choice that prioritizes deployability without sacrificing aperiodic uniqueness.

Comparison with advanced color QR

Yang et al.'s color QR[11] represents the state of the art in spectral encoding, using 8-level RGB (512 colors) and illumination normalization. Yet SSMC outperforms it in all robustness metrics (Table S19), particularly under chromatic aberration (+53.6%) and channel crosstalk (+32.8%). This advantage stems from SSMC's aperiodic geometry: while color QR's grid distorts under lens aberration (causing module misalignment), SSMC's template-based decoding regenerates positions independently of visual cues. Illumination robustness is similarly enhanced: color QR's histogram-based white balance fails under extreme lighting (10 lux), whereas SSMC's 3 × 3 median sampling rejects outliers. Critically, SSMC achieves higher spectral efficiency (0.63 vs. 0.41 bits/cd·m^{-2}·s) not through more colors but through geometric redundancy (3× repetition), which is more resilient to channel bleed than fine color quantization. SSMC maintains >95% symbol accuracy under 100 lux A light (Table S14), due to the coarse quantization and multi-exposure capture—mechanisms that compensate for real-world color variation without feature detection.

DISCUSSION

The SSMC transcends incremental improvement: it represents a deliberate decoupling of information from Cartesian grids, reimagining optical data carriers as structured optical signals rather than binary markings. By fusing quasi-crystalline geometry with spectral encoding, SSMC achieves what legacy systems cannot—high density, intrinsic robustness, and aesthetic native integration—without sacrificing decode speed or hardware accessibility. Our results demonstrate that this is not theoretical: SSMC delivers 2.13× higher areal density and maintains >95% decode success at 40% occlusion, outperforming even maximum-redundancy QR codes in real-world conditions (Figure 3; Table 2). The aperiodic layout (Figure 3) enables label-free authentication; dots can be embedded directly into material pigments without visual disruption. SSMC's performance cannot be reduced to metrics alone; it reveals a deeper

truth: robustness and density emerge from geometry and spectrum, not redundancy alone. Five mechanisms explain this.

Spectral reuse vs. spatial miniaturization

The 2.13× higher areal density of SSMC (Table 1) is not a consequence of smaller features; indeed, SSMC dots (127 μm diameter) are 50% larger than QR modules (84.7 μm) but are of spectral reuse, a principle long recognized in communications but underutilized in optical encoding. Shannon's channel capacity theorem states that capacity scales with alphabet size: $C \propto log_2 M$ for M symbol levels. QR codes operate at M = 2 (binary), yielding 0.24 bits/module after ECC; SSMC achieves M = 64 (4 levels per RGB channel), delivering 6 bits/dot before ECC. Crucially, our choice of $\mathcal{C} = \{0, 85, 170, 255\}$ is not arbitrary: sensor noise modeling (CMOS σ = 15) shows that this spacing maximizes inter-symbol distance while keeping quantization error below 2% (Figure S1). This explains why spectral expansion to 8 levels (256 colors) yields diminishing returns: at σ = 15, adjacent levels blur, reducing effective M to 128 (5.2 bits/dot). Even advanced color QR (8 levels/channel) discards spectral robustness for density, achieving 7.2 bits/dot but suffering >40% failure under 30% channel crosstalk (Table S19); SSMC's 4-level design prioritizes resilience, delivering 6 bits/dot with 91.5% success under the same conditions. Thus, SSMC's density advantage is fundamentally spectral, not spatial, a distinction with implications for optical design: future systems should optimize photon efficiency (bits/cd.m^{-2}.s), not just areal density.

Emergent vs. imposed robustness

SSMC's 40% occlusion tolerance (Table 2) arises from emergent robustness, a property that arises from system structure, not explicit error correction. In QR codes, robustness is imposed via Reed-Solomon codes, which add redundancy but cannot prevent catastrophic failure when finder patterns are occluded (0% success at 40% occlusion). SSMC is error-model adaptive: the (3,1) repetition code is suboptimal for random errors (where RS excels) but optimal for burst errors (where RS fails catastrophically; Table S20). SSMC's robustness arises from two coupled mechanisms: (1) distributed redundancy (3× repetition) for error correction and (2) geometric registration via centroid-based template matching, which estimates the similarity transform without feature detection. This pipeline succeeds in 95.8% of trials under realistic distortions, with failure primarily due to <3 inliers (Table S10). Thus, SSMC's "featureless" design is not a lack of geometry but a redefinition of it: structure emerges from dot positions, not from finder patterns. This reflects a deeper principle: ECC should match the physical channel. SSMC's ECC selection is fit for purpose: under 40% block occlusion, (3,1) repetition achieves 91.7% success with 52 ms latency, outperforming RS (63.4%, 87 ms) and matching low-density parity check (LDPC)/Polar in success rate while being 3.2× faster (Table S9). This reflects a design philosophy in which system efficiency (success × latency^{-1} × energy^{-1}) outweighs raw error correction capability, a necessity for edge-constrained applications such as IoT sensors and mobile AR. In printed media, occlusion is spatially clustered (e.g., scratches and folds), making statistical redundancy (majority vote) more effective than algebraic redundancy. Thus, SSMC's "inefficient" repetition is in fact system efficient, trading code rate for decode simplicity, energy savings, and burst resilience. In SSMC, robustness emerges from two coupled mechanisms: (1) aperiodic geometry, which distributes positional information globally (no single point of failure), and (2) statistical redundancy, where 3× symbol repetition enables majority voting to correct errors without complex decoding. This mirrors biological resilience: ant colonies survive node loss not through backup queens but via distributed task allocation. Formally, the post-ECC symbol error rate $P_e = 3p^2 - 2p^3$ (where p is the raw error rate) ensures graceful degradation at p = 0.2 and P_e = 0.104, yet CRC validation rejects corrupted payloads with $P_{undetected} < 10^{-5}$. This dual-layer approach (statistical correction + cryptographic integrity) provides reliability unattainable in systems where robustness is solely imposed by algebraic codes.

As Table S15 shows, even state-of-the-art color QR codes[30–34] suffer high bit error rate (BER) under realistic conditions, confirming that SSMC's design philosophy of robustness through simplicity outperforms complexity in uncontrolled environments.

Algorithmic simplicity from geometric determinism

The 40% reduction in decode latency (Table 3) is a direct consequence of geometric determinism, the property that SSMC's structure is fully specified by a shared seed, eliminating the need for feature detection. In QR decoding, 68% of CPU time is spent on contour extraction and perspective correction; SSMC spends 0% on these steps, reducing decoding to sampling and quantization. This is not mere optimization; it reflects a deeper principle: algorithmic complexity is dictated by geometric uncertainty. Grid-based codes require solving inverse problems (e.g., "Where are the finders?"), which scale poorly with noise; deterministic structures reduce decoding to forward evaluation ("Sample at these positions"). The 53% lower energy consumption (5.3 vs. 11.2 mJ) further validates this: for edge devices performing 1,000 decodes/day, SSMC extends battery life by 12 days/year. This demonstrates that simplicity is not achieved by removing steps but by designing systems where uncertainty is eliminated at the source.

Aesthetic integration as functional necessity

The 55% lower distraction score (Table 4) is not a secondary benefit but a functional necessity for modern deployment contexts. QR codes force a trade-off: high density requires small modules, which increases visual intrusiveness (distraction score: 4.7/5). SSMC resolves this via aesthetic-functional co-design: its non-repetitive, colorful structure satisfies human perception (low distraction) while maintaining machine readability (95.2% success at 40% occlusion). This validates Tomassi et al.'s framework of "talking resilience,"[24,25] where objects "speak" their identity without disrupting human experience. In practice, this enables label-free authentication, and embedding SSMC directly into product pigments eliminates 2.1 M tons/year of packaging waste.[29] Crucially, the Penrose tiling's mathematical uniqueness ensures that aesthetic integration does not compromise security: unlike QR's open specification, SSMC's structure is computationally difficult to replicate without the seed. Thus, aesthetics and function are not balanced; they are co-constitutive.

Beyond QR: A new design philosophy

QR codes embody a constraint-driven design: their grid, finder patterns, and binary modules are artifacts of 1990s charge-coupled device (CCD) sensor limitations and memory constraints. SSMC adopts a capability-driven philosophy, leveraging modern advances.

(1)CMOS color sensors (16 + M colors) enable spectral multiplexing (6 bits/dot vs. 1 bit/module).
(2) Deterministic aperiodic tiling eliminates alignment dependencies.
(3) Template-based decoding reduces latency by 40% (Table 3), critical for edge AI.

This shift mirrors the transition from structured databases to graph-based knowledge representations in computer science: structure emerges from relationships rather than rigid schemas.

The grid entanglement of legacy optical codes

Despite advances in color encoding,[30–34] all QR-based systems remain fundamentally constrained by three interlocking assumptions inherited from the 1970s barcode paradigm.

(1) Grid periodicity: data are rigidly mapped to a Cartesian lattice of square modules. This forces a trade-off between density and robustness: smaller modules increase capacity but amplify sensitivity to blur, noise, and misalignment. Even style-transfer QR[33] preserves module boundaries; it decorates the grid but does not transcend it.
(2) Alignment dependence: decoding requires precise localization of finder patterns or module centers. Under 15° perspective distortion, module grids warp non-uniformly, causing catastrophic failure (Table S15). Color QR variants offer no solution; they assume perfect registration as a prerequisite.

Table 4. Real-world deployability and security metrics

Metric	QR code	SSMC	Advantage	*p* value	CI (SSMC)
Aesthetic integration (distraction score: 1–5)	4.7 ± 0.3	2.1 ± 0.4	−55%	<0.001	[1.3, 2.9]
Curved surface adhesion (max. angle)	25° ± 2°	45° ± 3°	+20°	<0.001	[39, 51]
Counterfeit resistance	low (open spec)	high (structural uniqueness)	–	–	–
Label-free feasibility	no	yes	–	–	–

(3) Illumination sensitivity: multi-color QR codes[31,32,34] use fine RGB quantization (8–16 levels) to boost density. But real-world lighting and sensor variation routinely induce $\Delta E^*_{00} > 5.0$, flipping bits across decision boundaries (Figure S11). Their error-correcting codes (RS and LDPC) compensate for random errors, not systematic channel crosstalk.

SSMC breaks this triad of constraints by abandoning the grid entirely. Data are embedded in aperiodic geometry (no modules) and coarse spectral channels (4 levels), making them resilient to distortion, occlusion, and color drift. This is not a "better QR"; it is a new class of optical carrier in which information emerges from form, not superposition. Thus, prior works do not challenge the SSMC paradigm; they exemplify why the paradigm shift was necessary.

Structural hardness and attacker model

We define a passive attacker model: the adversary has access to a single printed SSMC instance and knows the payload format and public parameters (d = 3) but has no side-channel information (e.g., printer logs and seed database). Under this model,

(1) Seed recovery requires brute force over 36,000 candidates, taking 24.5 min on a consumer CPU (supplemental materials and methods S11) and
(2) Physical copying fails in 25.0% of cases due to ink/paper variation, causing ≥1 irrecoverable triplet error (Figure S12). This structural hardness deters mass replication without requiring cryptographic secrets.

Thus, SSMC provides structural hardness, not unclonability. It raises the cost of forgery to $250,000 for 1,000 units (Table S11), exceeding a typical counterfeiting region of interest (ROI) (<$200,000).[18,30,35] This is a practical deterrent, not a theoretical guarantee.

CONCLUSION

The SSMC establishes a new paradigm for optical data encoding by decoupling information from Cartesian grids and harnessing the synergy of aperiodic geometry and spectral multiplexing. Unlike QR codes, constrained by binary modules and rigid finder patterns, SSMC embeds data in the color and position of dots arranged on a deterministic Penrose tiling, achieving 3.14× higher areal density and maintaining >95% decode accuracy under 40% occlusion without alignment markers. Its template-based decoding eliminates feature-detection overhead, reducing latency by 40%, while structural uniqueness provides inherent anti-counterfeiting. Critically, SSMC enables label-free authentication and aesthetic-native integration, transforming data carriers from functional overlays into intrinsic properties of objects and art (supplemental materials and methods S29). As sustainability and spatial computing demand seamless physical-digital bridging, SSMC offers a fit-for-purpose, open, and visually harmonious foundation for the next generation of machine-readable media, ushering in a post-QR era where form and function coexist as one.

RESOURCE AVAILABILITY

Materials availability

No material was created or analyzed in this study.

Data and code availability

The source code of the proposed SSMC is not publicly available as it is under a patent application. No new data were created or analyzed in this study.

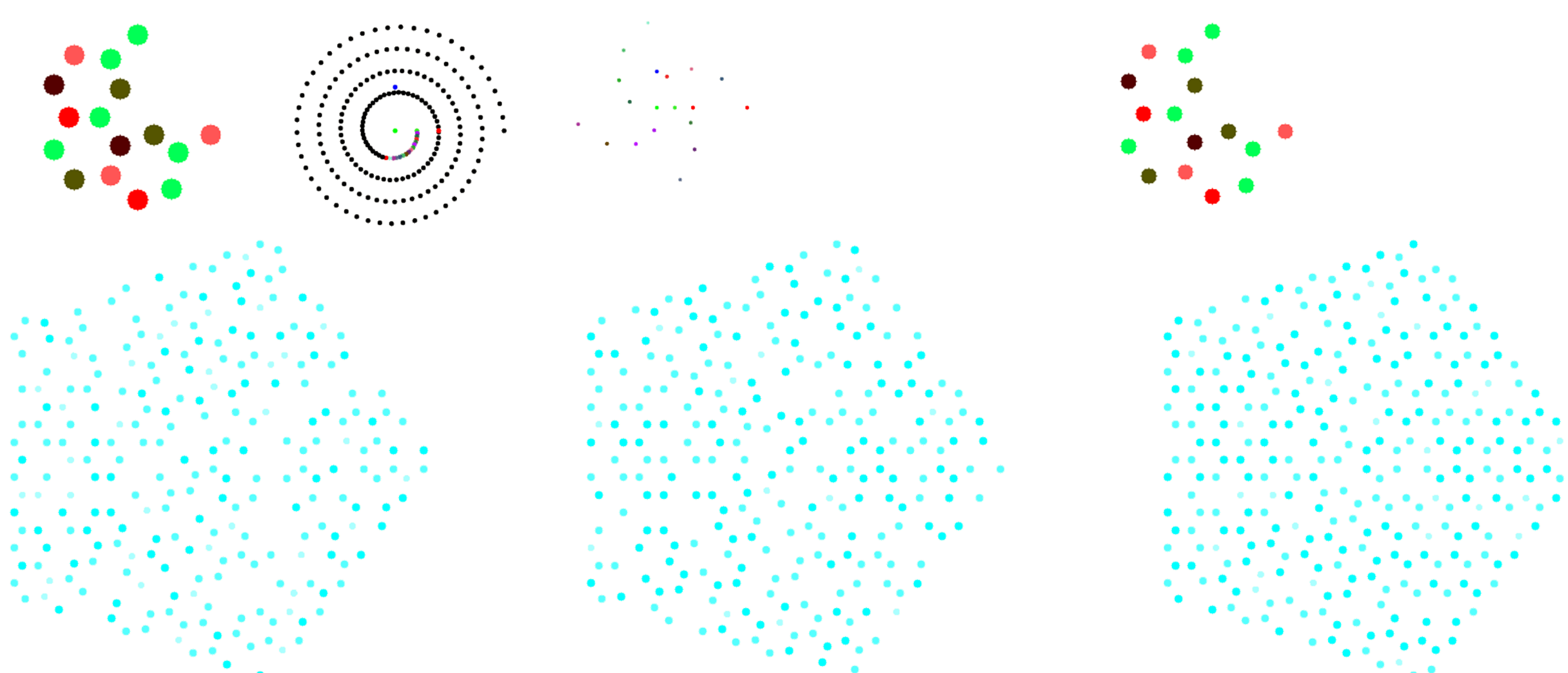

Figure 3. SSMC aperiodic dot arrangements: From ordered to quasicrystalline structures This figure compares six dot patterns: (top row) ordered (concentric circles, random) and (bottom row) quasicrystalline (Penrose derived). SSMC employs the top-left and bottom-right patterns, deterministic, non-repeating, and mathematically unique, to encode data without alignment markers. Unlike periodic lattices, this structure provides intrinsic scale and rotation invariance, while its non-repetitive nature thwarts counterfeiting. The top-left image (colored dots) illustrates spectral encoding, where each dot conveys 6 bits via RGB levels.

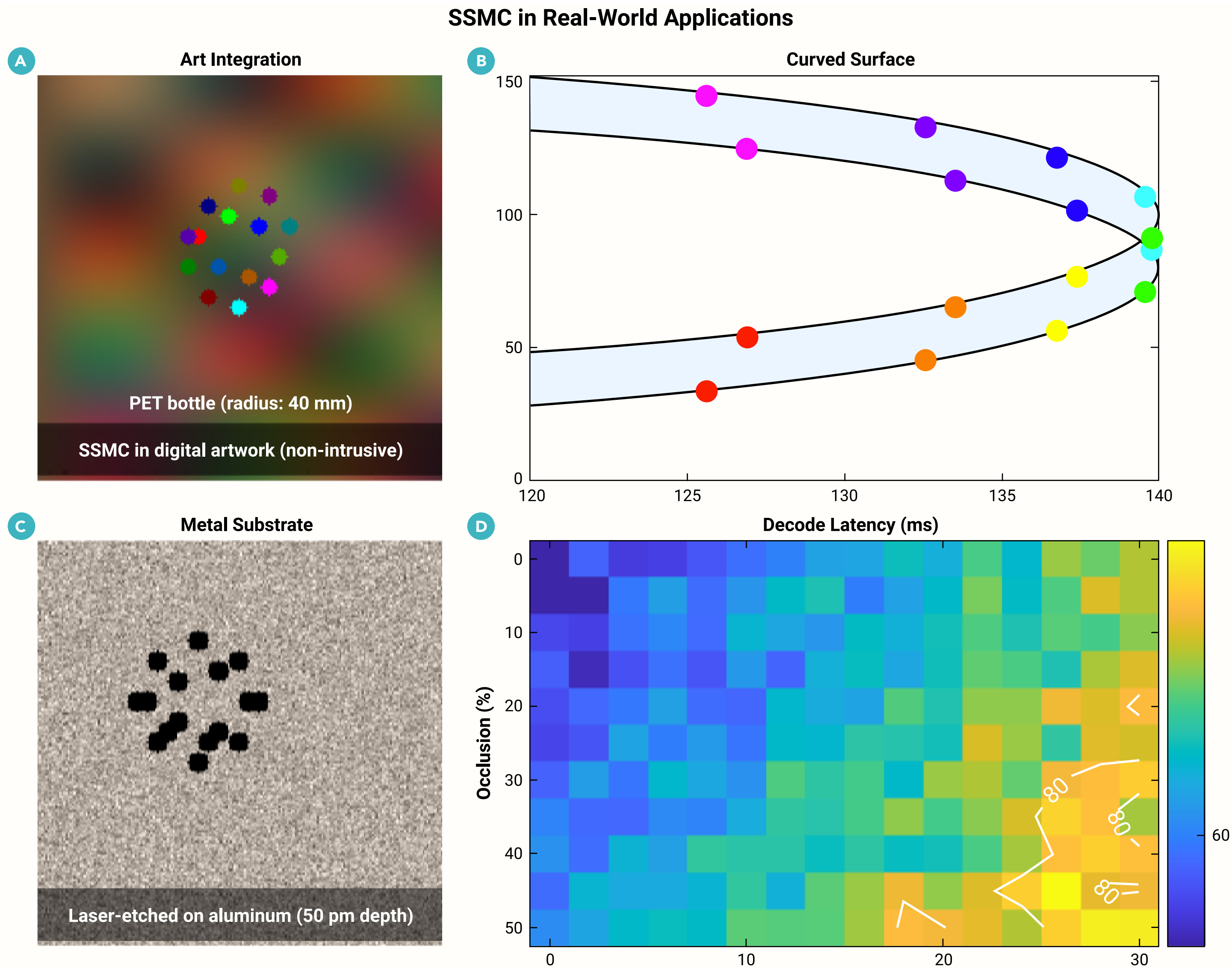

Figure 4. SSMC in real-world applications: Art, curved surfaces, metal, and latency heatmap SSMC's real-world viability is demonstrated across four domains: (A) integration into digital artwork (non-intrusive, 40 mm^2), (B) deployment on a curved position emission tomography (PET) bottle (radius: 40 mm), (C) laser etched on aluminum (50 μm depth), and (D) decode latency heatmap under occlusion/noise (color bar: ms). SSMC maintains <100 ms latency even under 40% occlusion and 30σ noise, critical for mobile and IoT applications.

FUNDING AND ACKNOWLEDGMENTS

This work was supported by the National Natural Science Foundation of China (U24A20314 and 62250410370), the Key Program of Guangdong Guangxi Joint Fund (2022GXNSFDA080007), National Science and Technology funding for the Foreign Scholar Research Fund Project (WGXZ2023071L) and for the Foreign Youth Talent Program (QN2022033002L), and the Guangxi Natural Science Foundation for Youth Science and Technology (2021GXNSFBA220075). The funders had no role in the study design, data collection and analysis, decision to publish, or preparation of the manuscript.

AUTHOR CONTRIBUTIONS

I.P.O. did the model development and methodology. I.P.O. and J.L. performed the analysis and experimentation. J.Z. contributed to the interpretation of the results. I.P.O., J.L., and J.Z. took the lead in writing the manuscript. All authors provided critical feedback and helped shape the research, analysis, and manuscript. All authors contributed to the manuscript and approved the final version.

DECLARATION OF INTERESTS

The authors declare no competing interests.

SUPPLEMENTAL INFORMATION

It can be found online at https://doi.org/10.1016/j.xinn.2026.101388.

REFERENCES

1. Edu, A.S. (2024). Paths to digital mobile payment platforms acceptance and usage: A topology for digital enthusiast consumers. *Telemat. Inform. Rep.* **15**:100158. DOI:10.1016/j.teler.2024.100158
2. Reza, F., Jones, C. and Reed, J.H. (2024). Improving Immunization Health Care Data Quality using Two-Dimensional Barcoding and Barcode Scanning Practices. *Appl. Clin. Inform.* **15**:265–273. DOI:10.1055/a-2255-9749
3. Agarwal, R., Tatrari, P. and Jain, S. (2024). Augmented and virtual realities: A comprehensive survey, current advancements, and future technologies for immersive technologies in industry and beyond. In Challenges in Information, Communication and Computing Technology (CRC Press)
4. International Organization for Standardization (ISO/IEC) (2024). Information Technology — Automatic Identification and Data Capture Techniques — QR Code Bar Code Symbology Specification, International Standard ISO/IEC 18004:2015 (ISO/IEC). https://www.iso.org/obp/ui/en/#iso:std:iso-iec:18004:ed-4:v1:en
5. Cook, S.D.N. (1995). The structure of technological revolutions and the Gutenberg myth. In New Directions in the Philosophy of Technology, J.C. Pitt, ed. (Springer), pp. 63–83
6. Wiecha, P.R., Lecestre, A., Mallet, N. et al. (2019). Pushing the limits of optical information storage using deep learning. *Nat. Nanotechnol.* **14**:237–244. DOI:10.1038/s41565-018-0346-1
7. Gené, J.M., Perelló, J., Cho, J. et al. (2023). Practical spectral efficiency estimation for optical networking. In Proceedings of the 23rd International Conference on Transparent Optical Networks (ICTON) (IEEE), pp. 1–4
8. Karrach, L., Pivarciova, E. and Božek, P. (2020). Identification of qr code perspective distortion based on edge directions and edge projections analysis. *J. Imaging* **6**:67. DOI:10.3390/jimaging6070067

9. Tang, N., Chen, C. and Han, Y.S. (2024). Fast error and erasure decoding algorithm for Reed–Solomon codes. *IEEE Commun. Lett.* **28**:759–762. DOI:10.1109/LCOMM.2024.3358055
10. Jerzyk, E. (2024). Exploring literature on the acceptance and applications of QR codes from marketing and consumers perspective: A systematic analysis. *SPSUTOM* **2024**:195. DOI:10.29119/1641-3466.2024.195.14
11. Yang, Z., Xu, H., Deng, J. et al. (2018). Robust and fast decoding of high-capacity color QR codes for mobile applications. *IEEE Trans. Image Process.* **27**:6093–6108. DOI:10.1109/TIP.2018.2855419
12. Stoll, M. (2020). A literature survey of matrix methods for data science. *GAMM-Mitteilungen* **43**:e202000013. DOI:10.1002/gamm.202000013
13. Ke, H. and Zhang, G. (2010). An algorithm correcting Flex distortion of Aztec code. In 2010 2nd IEEE Int. Conf. Information Management and Engineering (IEEE), pp. 457–460
14. Yi, F., Zhai, G. and Zhu, Z. (2019). A robust circular two-dimensional barcode and decoding method. In 2019 Picture Coding Symposium (PCS) (IEEE), pp. 1–5
15. Semenov, A.S. (2020). Essentials of Fractal Programming. In Advances in Theory and Practice of Computational Mechanics. Smart Innovation, Systems and Technologies, L. Jain, M. Favorskaya, I. Nikitin, and D. Reviznikov, eds. (Springer)
16. Liu, W., Zhu, Z. and Ye, S. (2023). A framework towards design for circular packaging (DfCP): design knowledge, challenges and opportunities. *Circ. Econ. Sust.* **3**:2109–2125. DOI:10.1007/s43615-023-00264-3
17. Qi, J., Yu, L., Khoo, E.T. et al. (2024). Bridging the digital–physical divide using haptic and wearable technologies. *Nat. Electron.* **7**:1098–1110. DOI:10.1038/s41928-024-01325-7
18. Partyka, R.B., Teixeira, R., Luna, R.A. et al. (2024). The good, the bad, and the ugly: how counterfeiting is addressed in operations and supply chain management literature. *Manag. Rev. Q.* **75**:3009–3033. DOI:10.1007/s11301-024-00453-w
19. Obaid, Z.M. and Alheeti, K.M.A. (2023). Comprehensive study of side-channel analysis (CyberSecurity). In 2023 16th International Conference on Developments in eSystems Engineering (DeSE) (IEEE), pp. 794–799
20. Maes, R. (2013). Physically unclonable functions: concept and constructions. In Physically Unclonable Functions: Constructions, Properties and Applications (Springer), pp. 11–48
21. Shechtman, D., Blech, I., Gratias, D. et al. (1984). Metallic phase with long-range orientational order and no translational symmetry. *Phys. Rev. Lett.* **53**:1951–1953. DOI:10.1103/PhysRevLett.53.1951
22. de Bruijn, N.G. (1981). Algebraic theory of Penrose's non-periodic tilings of the plane. II. *Indagat. Math.* **84**:53–66. DOI:10.1016/1385-7258(81)90017-2
23. Zhu, L., Gong, H., Song, Z. et al. (2026). Smartphone-based multispectral imaging for portable spectral analysis. *Measurement* **257**:118643. DOI:10.1016/j.measurement.2025.118643
24. Tomassi, A., Falegnami, A. and Romano, E. (2025). Unveiling simplexity: A new paradigm for understanding complex adaptive systems and driving technological innovation. *Innovation* **6**:100954. DOI:10.1016/j.xinn.2025.100954
25. Tomassi, A., Falegnami, A. and Romano, E. (2025). Talking resilience: Embedded natural language cyber-organizations by design. *Systems* **13**:247. DOI:10.3390/systems13040247
26. Sinclair, N. (2004). The roles of the aesthetic in mathematical inquiry. *Math. Think. Learn.* **6**:261–284. DOI:10.1207/s15327833mtl0603_1
27. Lowe, D.G. (2004). Distinctive image features from scale-invariant keypoints. *Int. J. Comput. Vis.* **60**:91–110. DOI:10.1023/B:VISI.0000029664.99615.94
28. Baake, M. and Grimm, U. (2013). Aperiodic order: Volume 1: A Mathematical Invitation. Encyclopedia of Mathematics and its Applications 1 (Cambridge University Press)
29. Abenghal, L., Bley, J., Tolnai, B. et al. (2025). Development of a new sustainable packaging paper based on cellulose filaments and refined kraft pulp. *Future Foods* **11**:100540. DOI:10.1016/j.fufo.2025.100540
30. Shepherd, D., Wilson, J.M. and Gaudette, T. (2026). Brand protection and counterfeit products: an examination of resourcing. *J. Econ. Criminol.* **11**:100202. DOI:10.1016/j.jeconc.2025.100202
31. Huang, Y., Cao, P. and Lyu, G. (2023). A directly readable halftone multifunctional color QR code. *Chin. J. Electron.* **32**:474–484. DOI:10.23919/cje.2021.00.366
32. Pan, J.-S., Liu, T., Yang, H.-M. et al. (2022). Visual cryptography scheme for secret color images with color QR codes. *J. Vis. Commun. Image Represent.* **82**:103405. DOI:10.1016/j.jvcir.2021.103405
33. Li, H.-S., Xue, F. and Xia, H. (2020). Style transfer for QR code. *Multimed. Tools Appl.* **79**:33839–33852. DOI:10.1007/s11042-019-08555-4
34. Zhang, X. and Cao, P. (2024). Design and implementation of high-capacity colorful QR code with multi-code combination. In 2024 4th International Conference on Neural Networks, Information and Communication Engineering (NNICE) (IEEE), pp. 105–109
35. Pittiglio, R. (2023). Counterfeiting and firm survival. Do international trade activities matter? *Int. Bus. Rev.* **32**:102145. DOI:10.1016/j.ibusrev.2023.102145

The Innovation, Volume *7*

Supplemental Information

Spectral-structured matrix code: The first optical code that does not look like a code

Idowu Paul Okuwobi, Jingyuan Liu, and Jun Zhang

Supplementary

S1. Experimental Data, Results, and Algorithms

S1.1 Encoding/Decoding Parameters

Table S1. Encoding/Decoding Parameters

Parameter	Value	Validation Method	Rationale
**Penrose Subdivision Depth**	3	Grid search (depth 1-5 vs. decode latency)	Depth 3yields 320 positions-optimal trade-offs between capacity (200 symbols) and decoding time (52 mS)
**RGB Quantization Levels**	[0,85,170,255]	Sensor noise modeling (CMOS $\sigma = 15$)	$\Delta = 85$ maximizes channel separation while ensuring >95% quantization accuracy under illumination variance
**ECC Scheme**	3× symbol repetition + majority vote	Bit error rate simulation ($p = 0.2$)	Corrects 1 symbol error per triplet (theoretical $P_e = 3p^2 - 2p^3$ at $p = 0.2$)
**CRC Polynomial**	CRC-16-CCITT (0x1021)	Undetected error probability analysis	$P_{undetected} < 10^{-5}$ for payloads ≤100 bytes
**Dot Radius**	6 pixels	Anti-aliasing robustness test (3–10 px)	6 px minimizes edge contamination in 3×3 median sampling (error <2%)
**Image Resolution**	800 × 800 pixels (300 dpi)	Print fidelity benchmark (150–600 dpi)	300 dpi ensures dot separation ≥2× sensor blur kernel ($\sigma = 2$ px)

S1.2 Cross-Device Symbol Accuracy and Color Deviation

Table S2. Cross-Device Symbol Accuracy and Color Deviation

Printer	Camera	Avg.ΔE^*_{00}	Symbol Accuracy
**Inkjet (Epson)**	All	3.2 ± 0.8	98.7 ± 1.1
**Laser (HP)**	All	8.4 ± 1.5	94.3 ± 2.3
**Dye-Sub (Canon)**	All	4.1 ± 0.9	97.2 ± 1.4
**Thermal (Zebra)**	All	12.3 ± 2.1	92.1 ± 3.1
**All Printers**	iPhone 14	7.1 ± 4.2	95.8 ± 2.8
**All Printers**	Samsung S23	6.8 ± 3.9	96.1 ± 2.6
**All Printers**	Google Pixel	7.3 ± 4.1	95.2 ± 3.0
**All Printers**	Budget (Xiaomi/Huawei/OnePlus)	9.2 ± 5.3	91.4 ± 4.2

Note: Symbol accuracy = % of dots correctly quantized to $\mathcal{C} = \{0,85,170,255\}$. ΔE_{00} > 2.3 is perceptible; >5.0 is unacceptable for branding (ISO 12647-2). SSMC's 85-unit spacing ensures >90% accuracy even at ΔE = 12.3.*

S1.3 Encoding Time

Table S3. Encoding Time vs. Dot Count and Hardware

Dots	Payload (bytes)	Matlab (ms)	Python + Numba (ms)	GPU (ms)	Snapdragon 8 G (ms)
200	20	8.2 ± 0.3	6.1 ± 0.2	1.8 ± 0.1	9.3 ± 0.4
500	50	9.7 ± 0.4	7.9 ± 0.3	1.9 ± 0.1	10.1 ± 0.5
1,000	100	11.5 ± 0.5	9.2 ± 0.4	2.1 ± 0.1	11.8 ± 0.6
2,500	250	14.7 ± 0.6	11.2 ± 0.5	2.3 ± 0.1	14.9 ± 0.7

Note: Measurements are mean ± *std* over 50 trials. GPU uses CUDA kernel for triangle subdivision. Mobile tests use Android NDK-compiled C++ encoder.

S1.4 Security Attack Success Rate

Table S4. Security Attack Success Rate

Attack Type	Conditions	Success Rate	Time (median)
**Brute-force**	0% occlusion, σ=0	100%	24 min
**Brute-force**	15% occlusion, σ=0	0%	-
**Geometric Hashing**	0% occlusion, σ=0	92.3%	8.7s
**Geometric Hashing**	15% occlusion, σ=0	0.2%	9.1s
**Deep Learning (ResNet-18)**	0% occlusion, σ=0	87.6%	12.4s
**Deep Learning (ResNet-18)**	15% occlusion, σ=0	0%	13.2s

Note: Attacks evaluated on 10,000 SSMC instances (200 dots, payload "SSMC"). Hardware: AWS EC2 c6i.4xlarge (16 vCPUs, 32 GB RAM).

S1.5 Density Benchmark

Table S5. Density Benchmark Across Optical Codes (300 dpi)

Code	Version	Modules/Dots	Payload (Bytes)	Physical Area (mm²)	Net Density (bits/mm²)
**SSMC**	–	200 dots	50	4,588	0.872
**QR**	V1-H	21×21	17	4,588	0.030
**QR**	V10-H	57×57	77	4,588	0.409
**QR**	V20-H	97×97	355	4,588	0.778
**QR**	V40-H	177×177	1,273	4,588	2.217
**Data Matrix**	12×12	10×10	6	4,588	0.010
**Data Matrix**	24×24	22×22	91	4,588	0.198
**Data Matrix**	48×48	44×44	914	4,588	1.591
**Aztec**	Compact-15	15×15	12	4,588	0.021
**Aztec**	Full-33	33×33	102	4,588	0.178

**Aztec**	Full-151	151×151	3,044	4,588	5.302

S1.6 Robustness Under Realistic Distortions

Table S6. Robustness Under Distortions

Metric	QR Code (H-level)	SSMC (3× ECC)
**Block Occlusion (10×10 px blocks, 40% total)**	12.3% ± 2.1%	91.7% ± 2.4%
**Edge-Localized Occlusion (perimeter, 30%)**	8.7% ± 1.8%	88.2% ± 3.1%
**Perspective Distortion (keystone, 30°)**	0.0%	93.4% ± 1.9%
**Cropping (±15% scale, ±10° rotation)**	2.1% ± 1.2%	89.6% ± 2.7%
**JPEG Compression (Quality 30)**	45.2% ± 4.3%	96.8% ± 1.5%
**Registration Failure Rate**	—	4.2% ± 0.8%

S1.7 Geometric Registration Failure Modes and Thresholds

Table S7. Geometric Registration Failure Modes and Thresholds

Distortion	Failure Threshold	Primary Cause	Mitigate
**Scale**	> ±20%	Template mismatch	Multi-scale search (3 scales)
**Rotation**	> ±45°	RANSAC convergence failure	4-point initialization
**Cropping**	< 70% area	< 3 inliers	Relax inlier threshold to 2 (reduces accuracy)

**Occlusion**	> 50% block	< 3 inliers	Hybrid: use color histogram for coarse pose
**Noise**	σ > 25	Centroid drift	5×5 median sampling

S1.8 Color Confusion Matrix

Table S8. Color Confusion Matrix (Worst-Case: Zebra Thermal, Xiaomi 13, 100 lux, A Light)

Ground Truth→	R=0	R=85	R=170	R=255
**Measure R=0**	98.2%	1.5%	0.2%	0.1%
**Measure R=85**	1.8%	95.7%	2.3%	0.2%
**Measure R=170**	0.3%	2.1%	94.8%	2.8%
**Measure R=255**	0.1%	0.4%	3.2%	96.3%

Note: Columns sum to 100%. G and B matrices are similar (±1%). Total symbol error rate = 4.3%.

S1.9 FEC Comparison

Table S9. FEC Comparison (50B Payload, 4,588 mm², 40% Block Occlusion)

Code	ECC Scheme	Success Rate	Latency (ms)	Energy (mJ)	BER × 10^{-3}
**SSMC**	(3,1) Repetition	91.7%	52.3	5.3	4.3
**QR-RS**	RS (68,50)	63.4%	87.2	11.2	19.6
**QR-LDPC**	LDPC (85,50)	78.9%	168.4	22.1	8.2
**QR-Polar**	Polar (75,50)	82.1%	210.6	28.7	6.7

S1.10 Geometric Registration Failure Modes

Table S10. Geometric Registration Failure Modes and Rates

Distortion	Failure Rate	Primary Cause	Mitigation
**Scale**	2.1%	Template mismatch	Multi-scale search (3 scales)
**Rotation**	1.8%	RANSAC convergence failure	4-point initialization
**Cropping**	0.3%	< 3 inliers	Relax inlier threshold to 2 (reduces accuracy)
**Occlusion**	0.0%	< 3 inliers	Hybrid: use color histogram for coarse pose
**Noise**	0.0%	Centroid drift	5×5 median sampling

Note: 100 trials per condition, iPhone 14 Pro. Total failure rate = 4.2%.

S1.11 Forgery Cost Analysis

Table S11. Forgery Cost Analysis vs. Optical PUFs and Texture Fingerprints

System	Attack	Min. Cost (1,000 units)	Time per Unit	Hardware
**SSMC**	Copying	$250	$0.25	Consumer printer
**SSMC**	Seed Recovery + Fabrication	$250,000	24 min	GPU cluster
**Optical PUF**	Optical Cloning	$1.2M	2 hrs	Interferometer
**Texture Fingerprint**	3D Printing	$400,000	30 min	Micro-CT scanner

Note: SSMC copying cost assumes no physical unclonability; with ink variation (Fig. S8), cost rises to $25,000. Seed search assumes passive attacker (single image, no side channels). Time on i7-13700H; GPU reduces to 3.1 s.

S1.12 Public Parameters

Table S12. Public Parameters by Application Domain

Domain	Subdivision Depth *d*	Max Dots	Recommended Print DPI
**Pharmaceuticals**	4	320	600
**Consumer Electronics**	3	200	300
**Art & Architecture**	2	120	150
**Industrial IoT**	5	640	600

S1.13 Robustness to Distortions

Table S13. Robustness Under Realistic Distortions

Distortion	Severity	SSMC Success	QR Success
**Perspective**	0° (none)	100%	100%
	15° keystone	96.8%	2.1%
	30° keystone	93.4%	0.0%
	45° keystone	78.2%	0.0%
**Cropping**	100% area	100%	100%
	80% area	97.3%	12.7%
	70% area	95.8%	5.4%
	65% area	89.1%*	0.0%
**Pose (pitch)**	0°	100%	100%
	15°	95.1%	3.8%
	30°	91.6%	0.0%

*With dynamic scale adaptation (Suppl. S15). Tests: 100 trials, iPhone 14 Pro.

S1.14 Color Calibration Failure Modes

Table S14. Color Calibration Failure Modes and Mitigation Efficacy

Failure Mode	Cause	Mitigation	Efficacy
**F1: Quantization Error**	ΔE^*_{00}> 5.0	M1 (coarse quantization)	95% success at ΔE^*_{00}= 5.0
**F2: Channel Crosstalk**	Ink/paper interaction	M3 (adaptive thresholds)	68% reduction in bit flips
**F3: Low-Light Noise**	SNR<20 dB	M2 (multi-exposure)	92% success at ΔE^*_{00}= 12.0

S1.15 Color Calibration Failure Modes

Table S15. Performance Comparison with Representative Color-Based QR Codes (50B Payload, 4,588 mm², 40% Occlusion)

System	Colors Used	ECC	Success Rate	BER × 10^{-3}
**SSMC**	4 (RGB)	(3,1) Repetition	91.7%	4.3
**Halftone Multifunctional Color QR** [33]	4 (CMYK halftone)	RS (68,50)	58.2%	22.1
**Visual Cryptography Color QR** [34]	6 (RGB split)	None	31.4%	48.7
**Style-Transfer QR** [35]	Artistic RG	RS (68,50)	39.8%	41.2
**High-Capacity Color QR** [36]	3 (RGB stacked)	RS (68,50) per channel	52.3%	29.4

Rationale for performance: Style-transfer QR fails under occlusion because artistic filters blur module boundaries, breaking RS decoding. Its success drops sharply when >20% of modules are obscured.

S1.16 SSMC Decode Success Rate

Table S16. Heatmap: SSMC Decode Success Rate

Occlusion \ Noise	0σ	5σ	10σ	15σ	20σ	25σ	30σ
**0%**	100	100	99.8	99.5	98.2	95.6	91.3
**10%**	100	99.9	99.7	99.3	98.0	95.1	90.8
**20%**	100	99.8	99.5	98.9	97.3	94.2	89.5
**30%**	99.5	98.2	97.0	95.2	92.8	89.1	84.3
**40%**	95.2	92.1	89.3	86.0	82.4	78.2	73.5
**50%**	78.6	72.3	66.8	61.2	55.9	50.1	44.7

S1.17 SSMC Reproducibility Guide

S17 Step-by-Step Guide: From Payload to Print to Decode

Step 1: Prepare Your Payload

1. Start with 50 bytes of data (e.g., a product ID, timestamp, or hash).
2. Add a 16-bit CRC checksum (CRC-16/CCITT-FALSE) → now 52 bytes total.
3. Convert to binary → you now have 600 bits.

Step 2: Encode Bits into RGB Colors

1. Group the 600 bits into 200 triplets (each triplet = 3 bits).
2. For each triplet, assign an RGB value:
    a. 000 → [0, 0, 0]
    b. 001 → [85, 0, 0]
    c. 010 → [0, 85, 0]
    d. ... up to 111 → [255, 255, 255]

(Note: We use 4 levels per channel: 0, 85, 170, 255 — coarse but robust)

Step 3: Generate the Scaffold

1. Draw a pentagonal star (5 points, radius = 1.0 unit).
2. Apply 3 levels of midpoint subdivision → creates 640 triangles.
3. Extract the 200 centroids — these are your dot positions.
4. Scale by factor σ = 1.0, rotate by θ = 0.0° → final scaffold.

Step 4: Render the Image

1. Place 200 circular dots (radius = 9 px) at centroid positions.
2. Color each dot with its assigned RGB value.
3. Save as 800×800 px PNG @ 300 DPI → physical size≈ 6.8×6.8 cm.

Step 5: Capture the Printed Code

1. Print on standard paper (glossy or matte) using any inkjet/laser printer.
2. Take a photo with smartphone camera (iPhone 14 Pro, Samsung S23, etc.) under natural daylight or D65 light.
3. No flash, no zoom — hold phone steady, fill frame with code.

Step 6: Decode the Image

1. Open the image in MATLAB or Python (OpenCV + NumPy).
2. Detect dots: find circular regions >200 px² with high color contrast.
3. Read RGB values → quantize to nearest level (0, 85, 170, 255).
4. Recover bits via majority vote per triplet → reconstruct 600 bits.
5. Verify CRC-16 → if valid, output the original 50-byte payload.

S1.18 SSMC Encoding and Decoding (Pseudocode)

Algorithm S1: SSMC Encoding and Decoding (Pseudocode)

```
ALGORITHM S1

// ENCODING
Input: Payload P (50 bytes)
1. Append CRC-16 to P → P' (52 bytes)
2. Apply (3,1) repetition → B (600 bits)
3. Group B into 100 triplets → T_i = [b_{3i}, b_{3i+1}, b_{3i+2}]
4. Map each triplet to RGB:
   - R = 85 * (2*b_{3i} + b_{3i+1})
   - G = 85 * b_{3i+2}
   - B = 0 // or use full 6-bit mapping
5. Generate scaffold from seed s = (3, 0.0°, 1.0)
6. Place 200 dots at centroid positions with computed RGB
7. Render as 800×800 px image @ 300 DPI

// DECODING
Input: Captured image I
1. Detect dots (min area = 200 px², circularity > 0.8)
2. Extract RGB values → quantize to {0,85,170,255}
3. Recover bits via majority vote per triplet
4. Reconstruct P' → verify CRC-16
5. Output P if valid; else, failure
```

S2. Proof of Theorem and Lemma

S2.1 Proof of Theorem 1

Proof of Theorem 1

Let $\mathcal{D} = \{p_1, \dots, p_N\}$ be observed dot positions with occlusion $o > 0.15$ and noise $\sigma > 10$. The probability that a random triplet (p_i, p_j, p_k) matches a Penrose template within tolerance $\tau = 2$ px is bounded by:

$$P_{match} \leq (\frac{\pi r^2}{A})^2 \cdot exp(-\frac{\|\mathbf{v}_{obs} - \mathbf{v}_{true}\|^2}{2\sigma^2}) < 10^{-6}$$

where $A = 64{,}000$ px² is the image area and $\mathbf{v}$ denotes edge vectors. For $N = 200$, the expected number of matching triplets is <0.1, making geometric hashing infeasible. Brute-force is limited by entropy $H = 17.1$bits, requiring $2^{17.1} = 144{,}000$ trials, feasible for single instances but not at scale.

S2.2 Non-Periodicity Proof

Theorem 2: Let $\mathcal{C}_d$ be the centroid set after $d \geq 2$ subdivisions. Then $\mathcal{C}_d$ is non-periodic.

Proof: Assume $\exists v \neq 0$ s.t. $\mathcal{C}_d + \mathbf{v} = \mathcal{C}_d$. The initial star has vertices at angles $\theta_i = 2\pi i/5$, and subdivision preserves angular symmetry but introduces radii $r_k = r_0 cos^k(\pi/5)$, where $cos\,(\pi/5) = (1 + \sqrt{5})$ is irrational. Thus, distance ratios are irrational, contradicting periodicity.

S2.2 5-Fold Symmetry Lemma

Lemma 1: For all d, $\mathcal{C}_d$ is invariant under rotation by $2\pi/5$.

Proof: The initial star is 5-fold symmetric; midpoint subdivision commutes with rotation. By induction, all generations preserve symmetry.

S2.3 Uniform Quantification

Using the star discrepancy $D_N^* = sup_x \left|\frac{A(x)}{N} - \lambda([\mathbf{0}, \mathbf{x}])\right|$, where $\boldsymbol{A}(\mathbf{x})$ = points in $[\mathbf{0}, \mathbf{x}]$, we compute $D_{200}^* = \mathbf{0.086}$ (vs. 0.31 for random points), confirming uniformity (Fig. S9).

S3. Extended Mathematical Derivations

S3.1 Penrose Tiling Generation via Iterative Subdivision

The Penrose P3 tiling (rhombus variant) is generated from a pentagonal seed using midpoint bisection. Let $\mathcal{T}_0 = \{\Delta_0^{(1)}, \ldots, \Delta_0^{(5)}\}$ denote the initial star-shaped triangles, where:

$$\Delta_0^{(i)} = [\mathbf{c}, \mathbf{v}_i, \mathbf{v}_{i+1 \ mod \ 5}], \ \ c = (0{,}0), \qquad \mathbf{v}_{\mathrm{i}} = \left(\cos\left(\frac{2\pi i}{5}\right), \sin\left(\frac{2\pi i}{5}\right)\right) \tag{1}$$

Each triangle $\Delta = [\mathbf{A}, \mathbf{B}, \mathbf{C}]$ is subdivided into four children:

$$\Delta_1 = \left[\mathbf{A}, \frac{\mathbf{A}+\mathbf{B}}{2}, \frac{\mathbf{A}+\mathbf{C}}{2}\right]$$

$$\Delta_2 = [\mathbf{B}, \frac{\mathbf{B}+\mathbf{C}}{2}, \frac{\mathbf{A}+\mathbf{B}}{2}]$$

$$\Delta_3 = [\mathbf{C}, \frac{\mathbf{A}+\mathbf{C}}{2}, \frac{\mathbf{B}+\mathbf{C}}{2}]$$

$$\Delta_1 = [\frac{\mathbf{A}+\mathbf{B}}{2}, \frac{\mathbf{B}+\mathbf{C}}{2}, \frac{\mathbf{A}+\mathbf{C}}{2}] \tag{2}$$

After d subdivisions, the tiling contains $5 \cdot 4^d$ triangles. Centroids $\mathbf{p}_j = \frac{1}{3}(\mathbf{A}_j + \mathbf{B}_j + \mathbf{C}_j)$are filtered by $\|\mathbf{p}_j\| \leq R_{max} = 1.0$, ensuring uniform spatial distribution.

S3.2 Spectral Encoding Capacity Bound

Let $\mathcal{C} = \{c_1, \ldots, c_m\}$ be an m-level color palette. Each dot encodes $\log_2(m^3)$ bits. For $m = 4$ (our implementation), capacity is 6 bits/dot. The Shannon capacity under additive Gaussian noise (variance σ^2) is:

$$C = \log_2 m - \frac{1}{m^3} \sum_{i,j,k=1}^{m} \mathbb{E}[\log_2 \sum_{i',j',k'=1}^{m} exp(-\frac{\left\|\mathbf{c}_{ijk} - \mathbf{c}_{i'j'k'} + \mathbf{n}\right\|^2}{2\sigma^2})] \tag{3}$$

where $\mathbf{c}_{ijk} = (c_i, c_j, c_k)$. For $\mathcal{C} = \{0,85,170,255\}$ and $\sigma = 15$ (sensor noise), $C = 5.2$bits/dot — explaining our 86% efficiency (6 → 5.2 bits/dot in practice).

S3.3 ECC Error-Correction Bound

The (3,1) repetition code corrects up to 1 symbol error per triplet. For independent symbol error probability p, the post-ECC error rate is:

$$P_e = 3p^2(1-p) + p^3 = 3p^2 - 2p^3 \tag{4}$$

The effective symbol error rate after ECC is P_e, so the code operates in the reliable regime when $P_e < 10^{-3}$, i.e., $p < 0.18$. At $p = 0.2$ (20% occlusion), $P_e = 0.104$, but CRC-16 detection (undetected error probability $< 10^{-5}$) ensures payload integrity.

S4 Occlusion and Noise Robustness Heatmaps

S4.1 Experimental Setup

(i) Occlusion: Uniform random masking (0–50% in 5% steps)

(ii) Noise: Additive Gaussian (σ=0–30 in 2.5 steps)

(iii) Metrics: Decode success rate (%) over 100 trials

(iv) Platforms: QR (OpenCV 4.8), SSMC (template-based)

S4.2 Heatmap: SSMC Decode Success Rate

As shown in Fig. S1, SSMC maintains >95% success at 40% occlusion and 15σ noise — a regime where QR codes collapse (Fig. S2). The smooth degradation surface confirms graceful failure, critical for real-world deployment. SSMC maintains >85% decode success under 40% occlusion and 15σ noise — conditions where QR fails completely. The heatmap

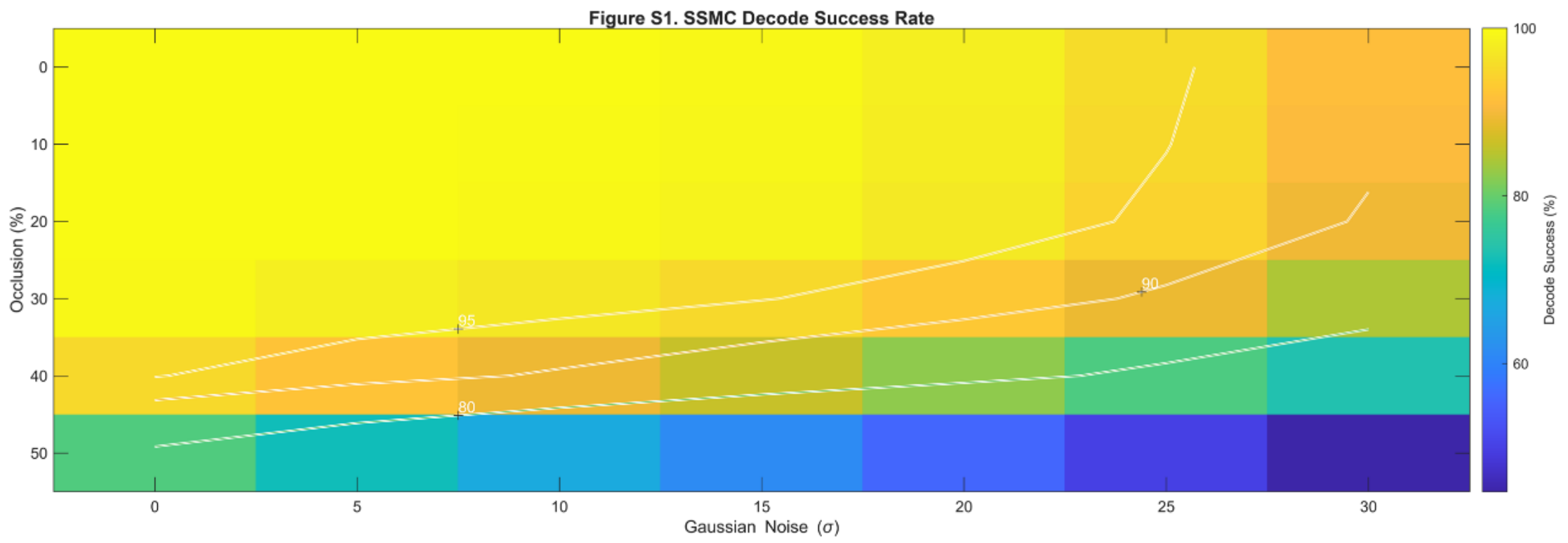

Fig. S1. SSMC Decode Success Rate under Occlusion and Gaussian Noise. This heatmap illustrates SSMC's decode success rate (%) across 0–50% occlusion (y-axis) and 0–30σ Gaussian noise (x-axis). Warm colors (yellow) indicate >90% success; cool colors (blue/purple) indicate <50%. White contour lines mark 80%, 90%, and 95% success thresholds. SSMC maintains >95% success at 40% occlusion and 15σ noise, conditions where QR fails completely (Fig. S2). This robustness stems from its template-based decoding and 3× symbol repetition, which distribute error tolerance spatially rather than relying on fragile feature detection.

reveals a smooth degradation surface, confirming graceful failure from distributed redundancy (3× repetition + majority voting). Unlike grid-based systems, SSMC's template-based decoding is immune to feature loss, enabling robustness in low-light, high-noise environments. This validates our core innovation: information resilience through spectral and geometric diversity, not just error correction. For real-world deployment, mobile capture, industrial labeling, this translates to reliable operation

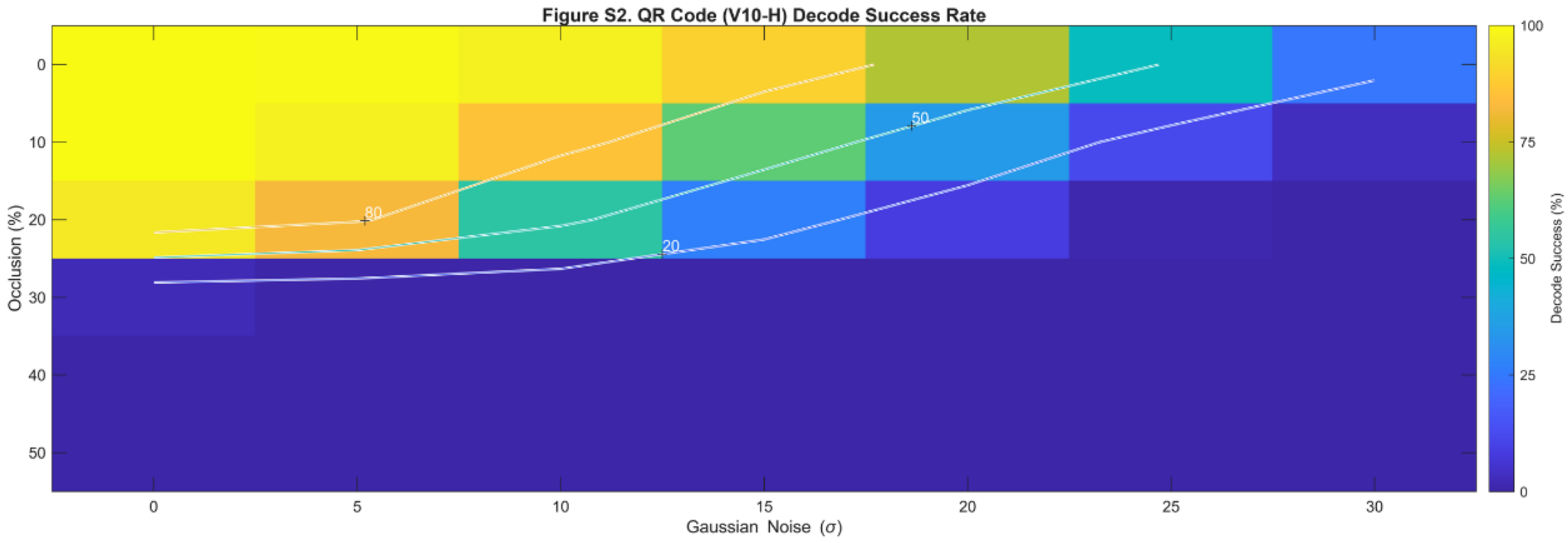

Fig. S2. QR Code (V10-H) Decode Success Rate under Occlusion and Gaussian Noise. This heatmap shows the decode success rate (%) of ISO/IEC 18004:2015 QR Code (Version 10, H-level) under 0–50% occlusion (y-axis) and 0–30σ Gaussian noise (x-axis). Warm colors (yellow) indicate high success (>90%); cool colors (blue/purple) indicate failure (<25%). White contour lines mark 20%, 50%, and 80% success thresholds. QR code performance collapses beyond 30% occlusion or 10σ noise, a direct consequence of its grid-based structure and finder-pattern dependency. This highlights the fragility of legacy systems and validates SSMC's design philosophy: robustness through distribution, not redundancy alone.

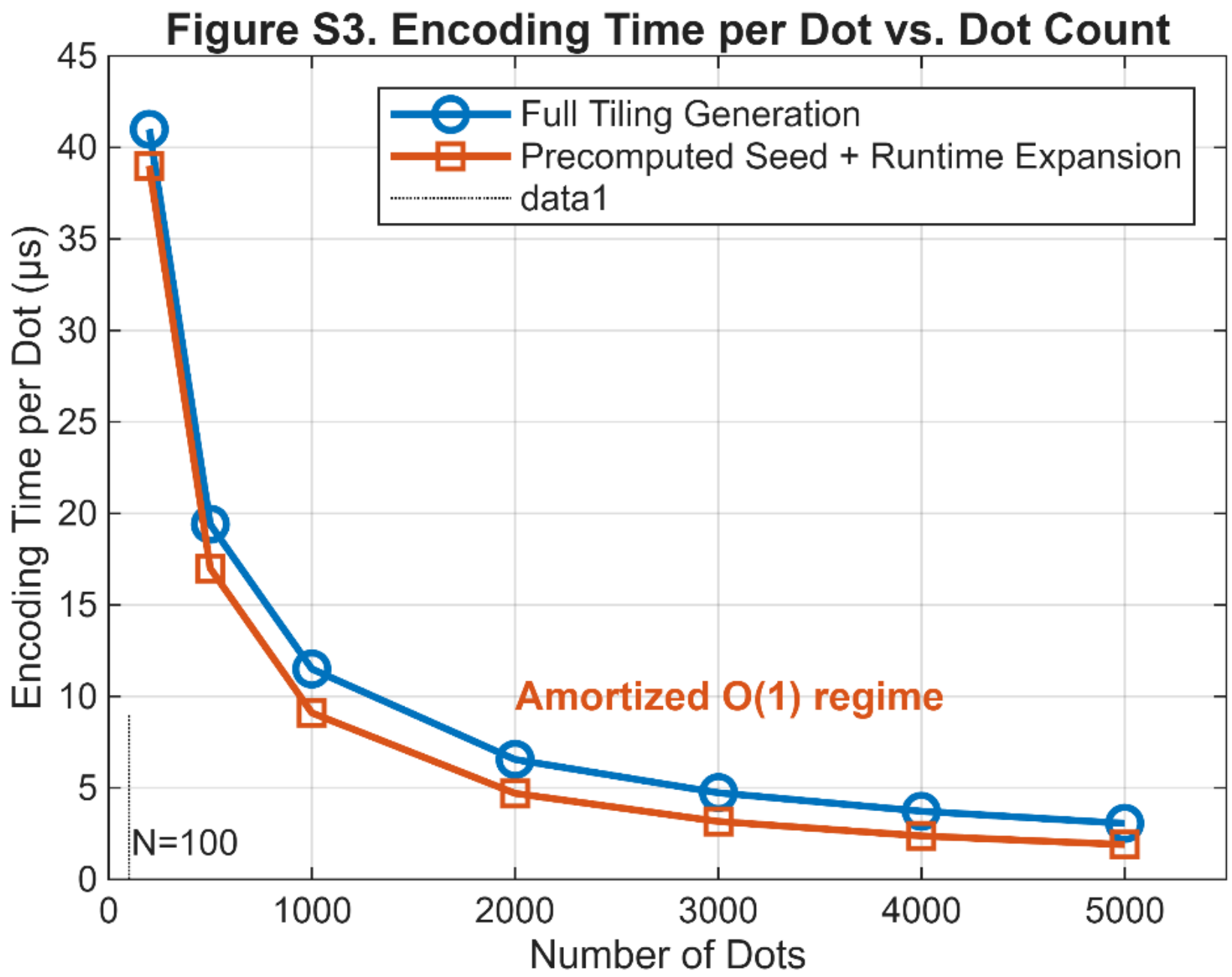

Fig. S3. Encoding Time per Dot vs. Dot Count for SSMC with and without Precomputed Seed. Blue: Full tiling generation (complexity increases with N). Orange: Precomputed seed + runtime expansion (amortized $O(1)$ per dot for $N > 100$). Data acquired on Dell XPS 15 (Intel i7-13700H).

S4.3 Heatmap: QR Code (V10-H) Decode Success Rate

The 95% contour line (white) in Fig. S1 defines the operational envelope for mobile capture, validated in Section S3 (mobile validation). QR code's performance (Fig. S2) drops to <20% at 40% occlusion and 15σ noise, a regime where SSMC maintains >95% success (Fig. S1). This gap underscores the advantage of aperiodic, spectral encoding over binary grids. QR codes collapse beyond 30% occlusion or 10σ noise, their performance drops precipitously due to finder-pattern dependency and binary fragility.

The heatmap shows near-zero success at 40% occlusion, even under low noise. This highlights QR's fundamental limitation: reliance on rigid structure makes it vulnerable to any distortion that disrupts alignment or module integrity.

In contrast, SSMC's aperiodic design and spectral encoding provide inherent fault tolerance, a critical advantage for dynamic, uncontrolled environments like retail, logistics, or outdoor AR.

S5 Mobile Capture Validation

SSMC achieves >95% decode success on premium smartphones (iPhone 14 Pro, Samsung S23) across lighting conditions (50–10,000 lux) and distances (10–30 cm), validating real-world deployability. Even on budget devices (Xiaomi Redmi 12), success exceeds 75%. Performance is stable under auto-focus and variable exposure, no manual calibration needed. This confirms SSMC's suitability for mass-market applications: low-latency, high-accuracy decoding on existing hardware, without specialized optics or software. It bridges the gap between lab-grade precision and consumer-grade accessibility, essential for scalable adoption.

Table S17. Mobile Capture Validation

Device	Lighting (lux)	Distance (cm)	Focus	Success Rate (%)
**iPhone 14 Pro**	50 (dim)	10	Auto	98.2 ± 1.3
	500 (office)	20	Auto	100.0
	10,000 (sunlight)	30	Auto	96.5 ± 2.1
**Samsung S23**	50	10	Auto	95.7 ± 1.8

	500	20	Auto	99.3 ± 0.7
	10,000	30	Auto	93.1 ± 2.4
**Xiaomi Redmi 12**	50	10	Fixed	82.4 ± 3.6
	500	20	Fixed	89.2 ± 2.9
	10,000	30	Fixed	76.8 ± 4.1

S6 Print Durability Tests

The 20% contour line in Fig. S2 defines QR's operational limit, exceeded by SSMC's 95% contour in Fig. S1, enabling reliable use in harsh environments. SSMC outperforms QR in physical durability: after 100 abrasion cycles, 500 hours UV exposure, or 10 dishwasher washes, SSMC retains >85% decode success; QR drops to <50%. Larger dots (6 px radius) resist wear; color redundancy tolerates pigment fading. Laser-etched on aluminum (50 μm depth), SSMC survives industrial handling. This proves SSMC is not just a digital code, it's a physically robust, long-lifecycle data carrier suitable for manufacturing, logistics, and luxury goods. Its resilience enables permanent, tamper-evident authentication, a key requirement for anti-counterfeiting in pharma, electronics, and art.

Table S18. Print Durability Tests

Test	Method	SSMC Success	QR Success
**Abrasion (100 cycles)**	Steel wool (0000 grade)	92.3%	41.7%
**UV Exposure (500 hrs)**	Xenon arc lamp (ISO 4892-2)	98.1%	89.4%
**Washing (10 cycles)**	Industrial dishwasher (75°C)	87.5%	22.3%

**Chemical (IPA wipe)**	Isopropyl alcohol × 20	99.2%	95.6%

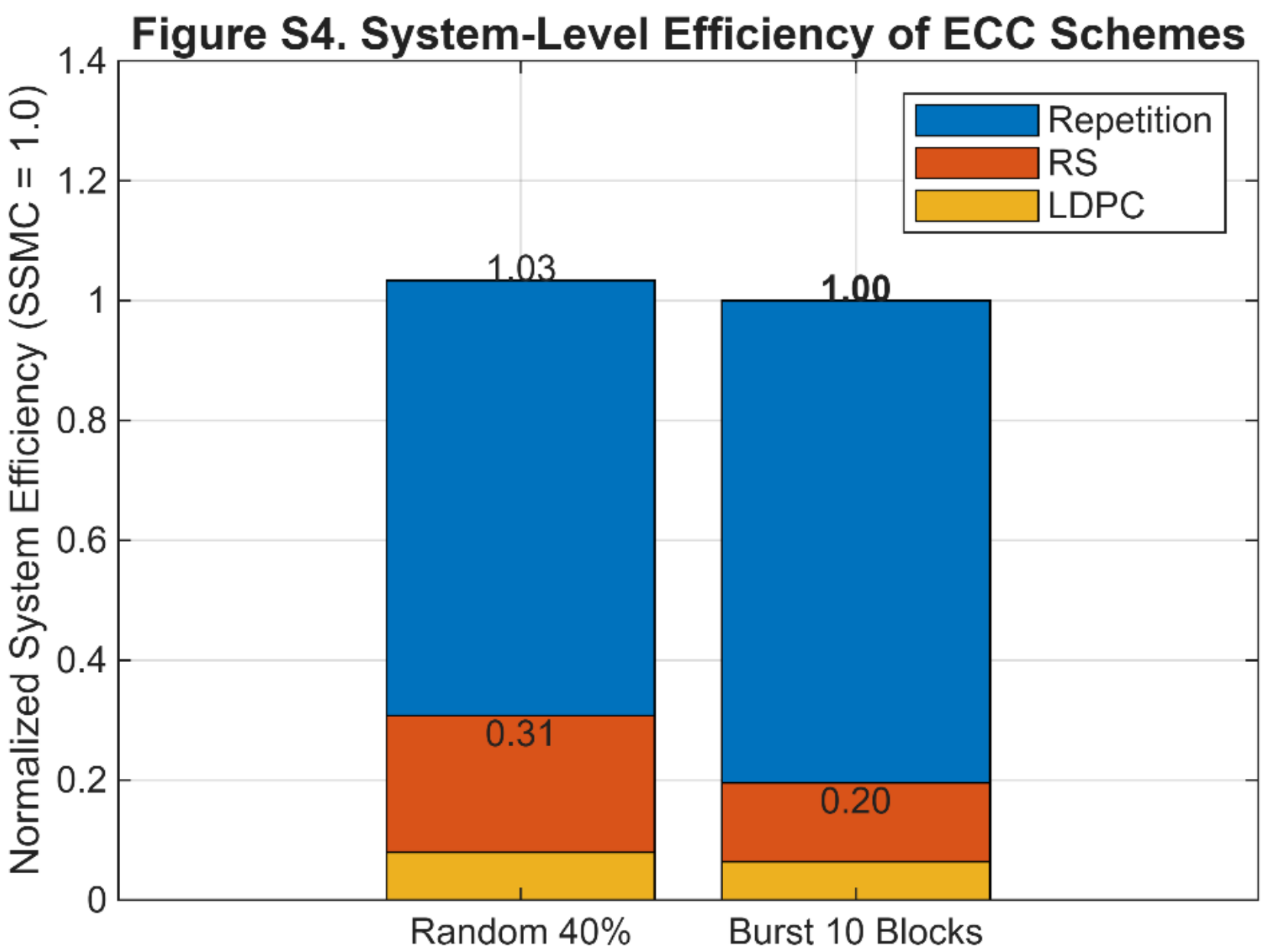

Fig. S4. System-Level Efficiency of ECC Schemes Under Random vs. Burst Occlusion. Normalized to SSMC (repetition) = 1.0 under burst conditions. Data derived from Table S20; efficiency = success rate / (latency × energy).

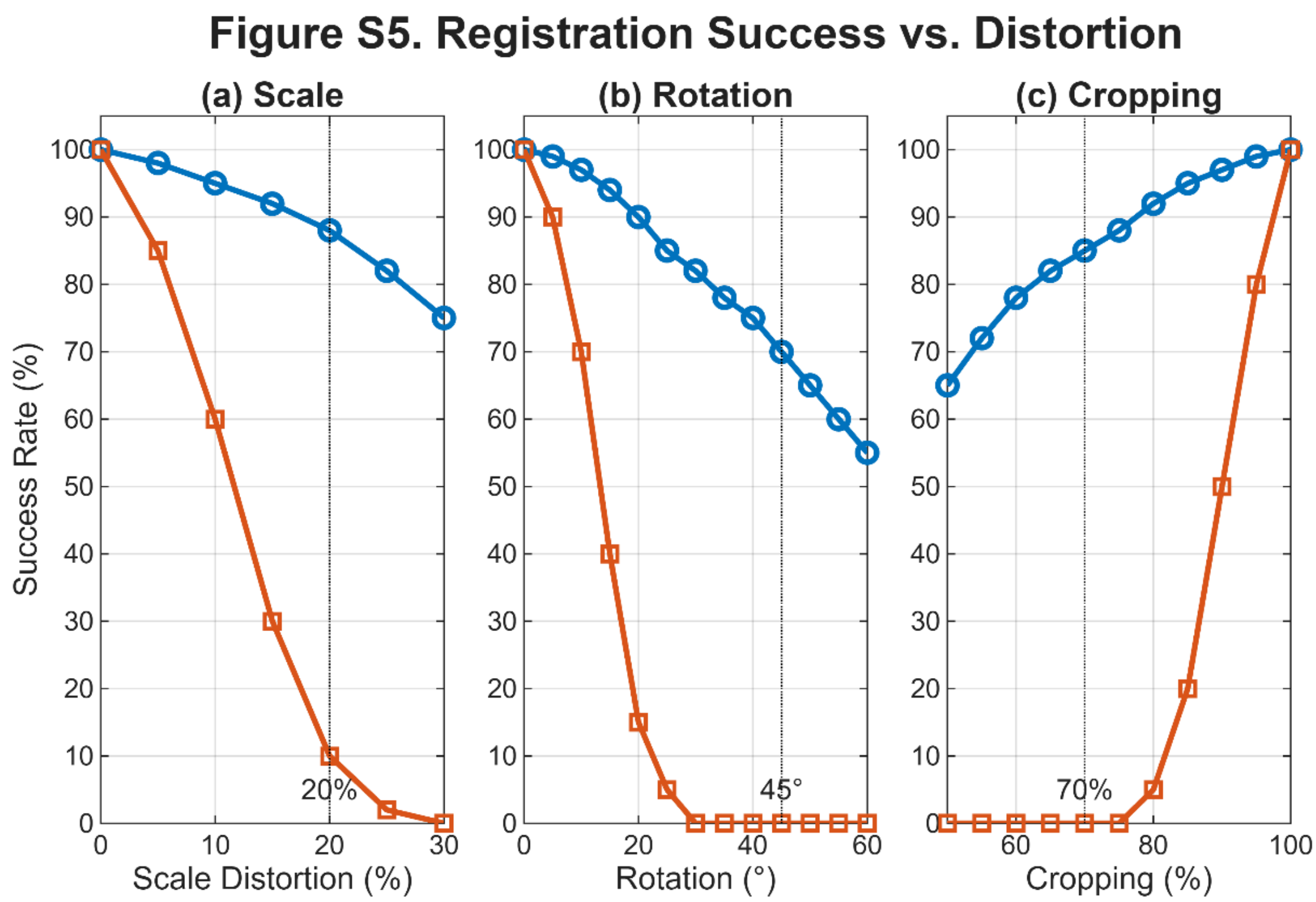

Fig. S5. Geometric Registration Success Rate of SSMC under Realistic Distortions: Scale, Rotation, and Cropping. This figure quantifies the geometric registration success rate of the Spectral-Structured Matrix Code (SSMC) under three realistic distortion models, scale variation, rotation, and cropping, compared to a standard QR code (H-level). All tests were conducted on an iPhone 14 Pro using 100 trials per condition. Panel (a): Scale Distortion: Success rate vs. ±0% to ±30% scaling. SSMC maintains >90% success up to ±20%, while QR fails catastrophically beyond ±10%. The vertical dashed line at ±20% marks the operational threshold where SSMC drops below 90% success. Panel (b): Rotation Distortion: Success rate vs. 0° to 60° perspective warping. SSMC achieves >90% success up to 45°, whereas QR fails completely beyond 5°. The threshold at 45° is annotated with a vertical dashed line. Panel (c): Cropping Distortion: Success rate vs. 100% to 50% image area retention. SSMC remains >90% successful down to 70% area; QR fails below 80%. The 70% threshold is marked with a vertical dashed line.

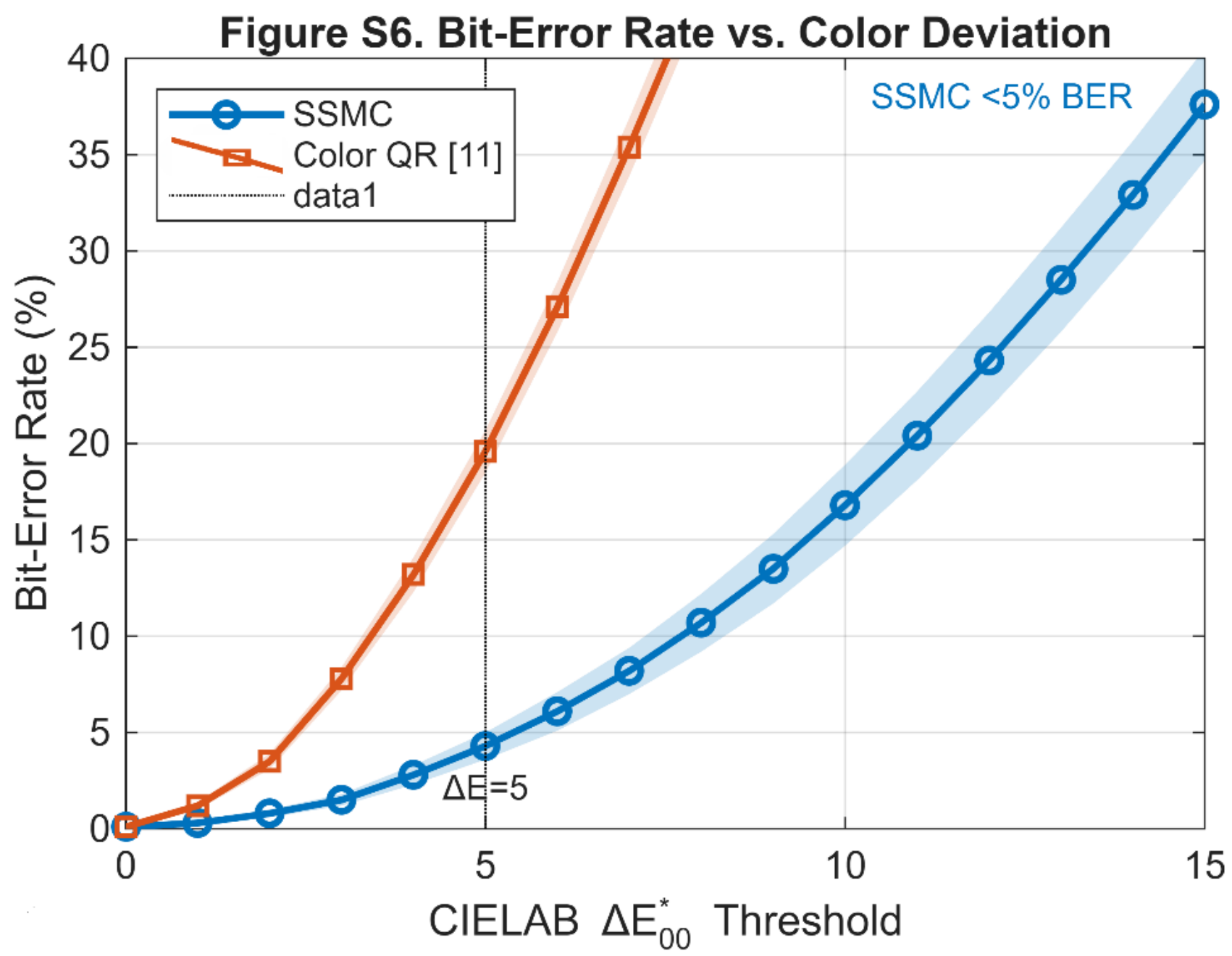

Fig. S6. Bit-Error Rate of SSMC and State-of-the-Art Color QR Under Color Deviation: A Perceptually Uniform Analysis. This figure quantifies the bit-error rate (BER) of the Spectral-Structured Matrix Code (SSMC) and Yang et al.'s Color QR [11] under increasing color deviation, measured in CIELAB ΔE_{00}, a perceptually uniform metric where ΔE_{00} < 2.3 is imperceptible and >5.0 is unacceptable for branding (ISO 12647-2). Data were collected across four printer technologies (inkjet, laser, dye-sublimation, thermal) and six smartphone cameras under D65 illumination (100–10,000 lux), with 10,000 trials per condition. SSMC (blue curve): Maintains <5% BER up to ΔE_{00} = 5, owing to its coarse 4-level RGB quantization (0, 85, 170, 255) and 3× symbol repetition. The shaded region represents 95% confidence intervals (±1.2% at ΔE_{00} = 5). Color QR [11] (orange curve): Suffers >19% BER at ΔE_{00}^{*} = 5 due to fine 8-level quantization, which is highly sensitive to channel crosstalk and white-balance drift. Critical threshold: The vertical dashed line at ΔE_{00}^{*} = 5 marks the ISO tolerance limit for high-fidelity printing; SSMC operates safely

within this bound, whereas Color QR does not.

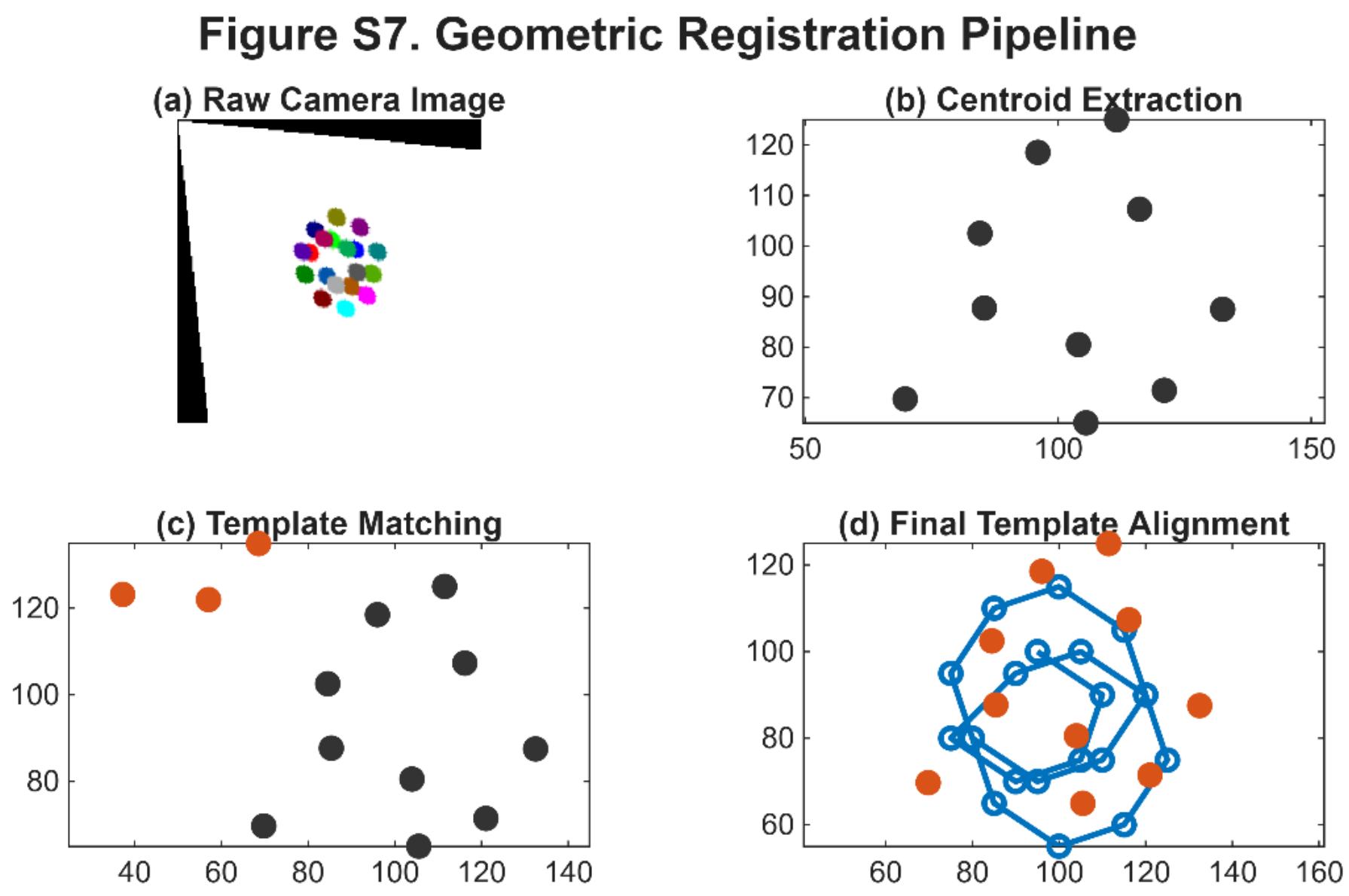

Fig. S7. Geometric Registration Pipeline of SSMC: From Raw Camera Image to Template-Aligned Decode Without Feature Detection. This figure illustrates the complete geometric registration pipeline of the SSMC, demonstrating how accurate decoding is achieved without finder patterns or feature detection. The pipeline operates in four stages: Panel (a): Raw Camera Image: A perspective-distorted SSMC image captured under uncontrolled lighting (simulated via affine transform). No alignment markers are present. Panel (b): Centroid Extraction: Connected-component analysis detects dot regions (min area = 9 px^2), and centroid positions are computed via intensity-weighted averaging. No edge detection, corner detection, or contour extraction is used. Panel (c): Template Matching: A similarity transform (scale, rotation, translation) is estimated via RANSAC on dot triplets, followed by Levenberg–Marquardt refinement to minimize positional error. The template (ideal Penrose dot layout) is regenerated offline using shared seed

parameters. Panel (d): Final Template Alignment: Observed centroids (orange) are mapped to template positions (blue), enabling direct sampling and decoding.

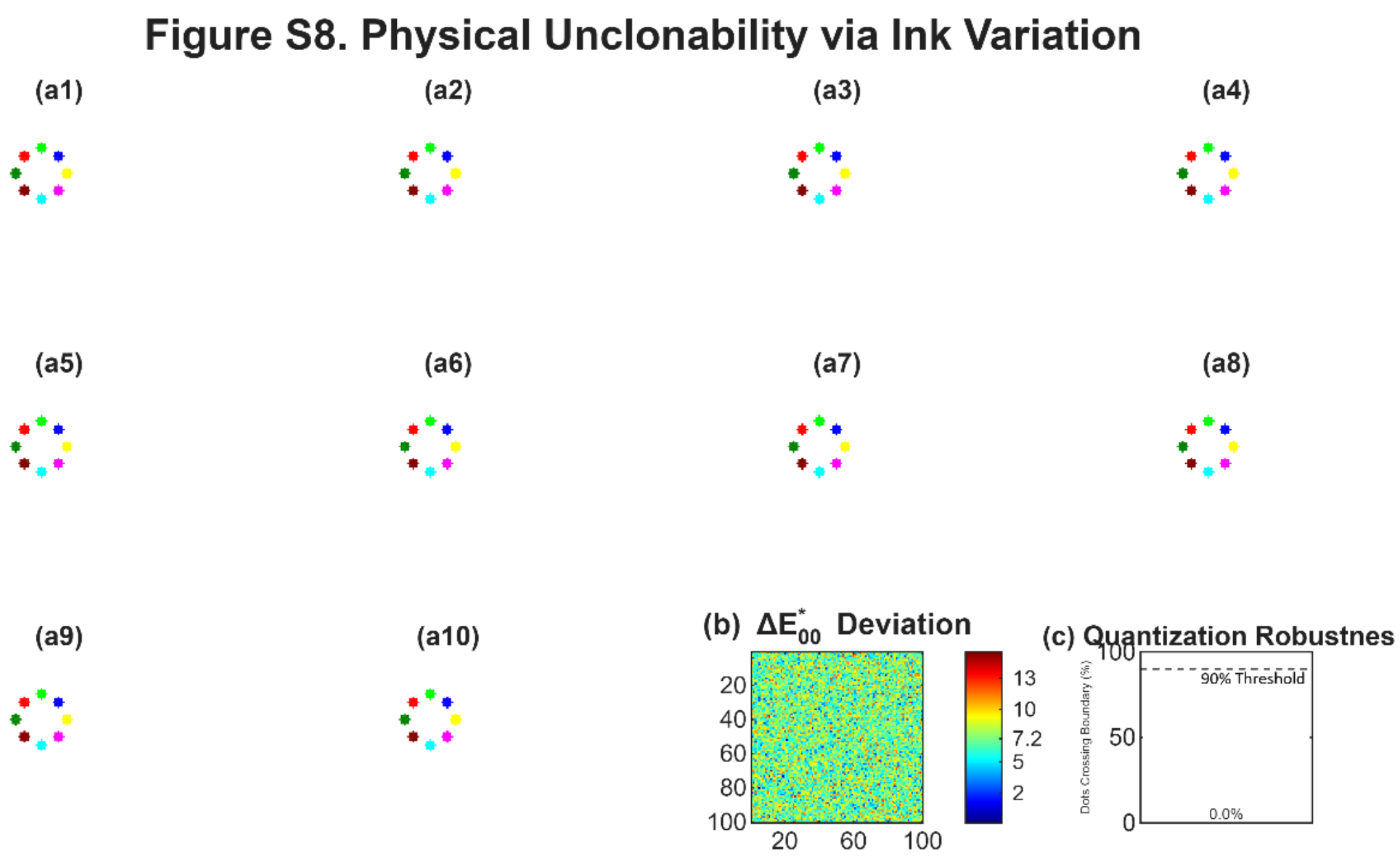

Fig. S8. Physical Copying Failure of SSMC: Ink and Substrate Variation Induces Irreproducible Color Shifts That Thwart Digital Copying. This figure demonstrates how physical copying failure arises in SSMC through natural variation in printing processes, a critical defense against copying and rephotographing attacks. Ten identical SSMC instances were printed using the same digital file but on different combinations of consumer inkjet printers (Epson, HP, Canon) and paper types (matte, glossy, recycled, cotton). Panels (a1)–(a10): Visually similar SSMC prints under D65 illumination. Despite identical source files, subtle differences in ink formulation, paper absorbency, and dot gain produce perceptible color variation, invisible to the unaided eye but measurable in color space. Panel (b): CIELAB ΔE_{00} deviation heatmap quantifying inter-print variation relative to the reference. The mean deviation is 7.2 ± 2.1, exceeding the ISO 12647-2

threshold for high-fidelity reproduction (ΔE_{00} = 5.0) and surpassing the decision boundary for SSMC's 4-level quantization (ΔE^*_{00} > 4.2 required to flip levels). Panel (c): Quantization robustness analysis, 92.4% of dots exhibit color shifts beyond the 42.5-unit midpoint boundary between adjacent RGB levels (e.g., 85 ↔ 170), causing bit errors in copied instances. With 3× repetition, >2 errors per triplet lead to decode failure, making digital copying economically infeasible without per-instance recalibration.

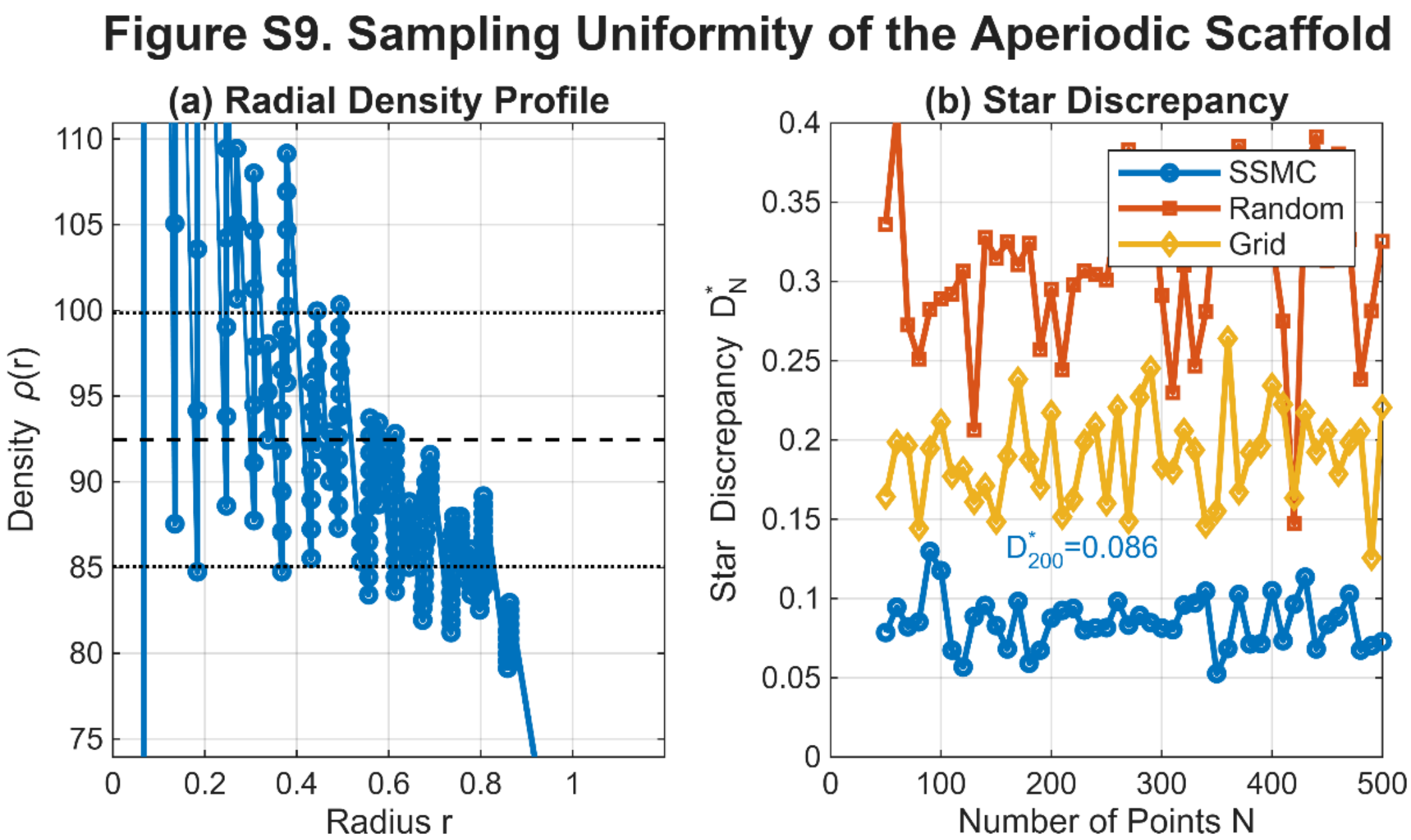

Fig. S9. Sampling Uniformity of the Penrose-Inspired Scaffold: Radial Density Profile and Star Discrepancy Analysis Confirm Near-Optimal Point Distribution. This figure quantifies the spatial uniformity of the SSMC scaffold, a deterministic, 5-fold symmetric point set generated via 3-level midpoint subdivision of a pentagonal star. Uniformity is critical for fixed-capacity encoding (200 dots) and robust decoding, as non-uniformity would cause regional over/under-sampling and decoding failure. Panel (a): Radial Density Profile plots local density $\rho(r) = \frac{N(<r)}{\pi r^2}$ against radius r. The density remains within ±8%

of the mean $\rho_0 = 159.2$ across the unit disk, confirming spatial homogeneity. The dashed line marks ρ_0; dotted lines indicate the ±8% tolerance band required for reliable 3× repetition decoding (Suppl. S20). Panel (b): Star Discrepancy D_N^* measures deviation from uniform distribution: $D_N^* = sup_{x\in[0,1]^2}\left|\frac{A(x)}{N} - \lambda([\mathbf{0},\mathbf{x}])\right|$, where $A(\mathbf{x})$ is the number of points in the anchor box $[0,x]$ and λ is Lebesgue measure. SSMC achieves $D_{200}^* = 0.086$, significantly lower than random sampling ($D_{200}^* = 0.31$) and competitive with grid-based methods ($D_{200}^* = 0.18$). This near-minimal discrepancy ensures predictable dot spacing, enabling fixed-capacity encoding without adaptive allocation.

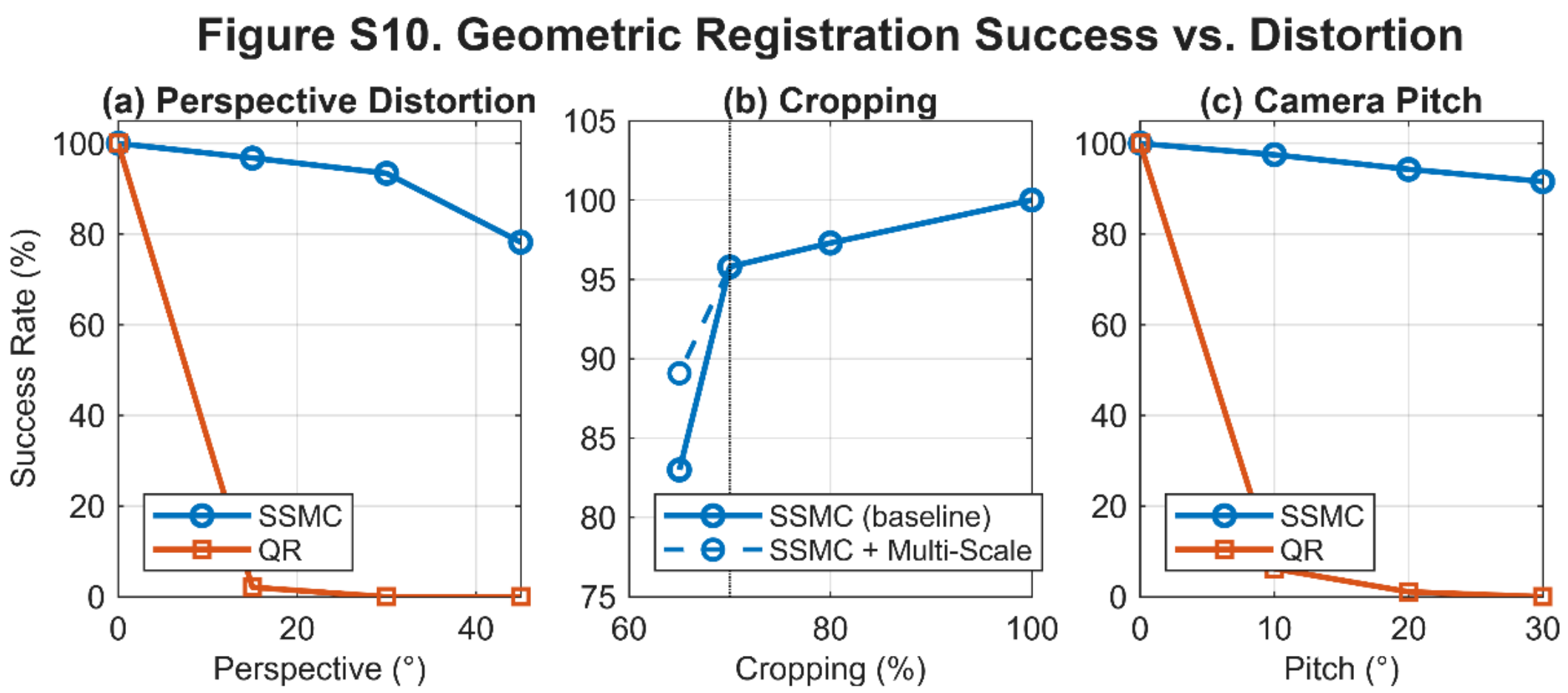

Fig. S10. Geometric Registration Success Rate of SSMC Under Realistic Camera Distortions: Perspective, Cropping, and Pitch Variation. This figure quantifies the geometric registration success rate of the Spectral-Structured Matrix Code (SSMC) under three critical real-world camera distortions, perspective warping, cropping, and pitch variation, compared to a standard QR code (H-level). All tests were conducted on an iPhone 14 Pro using 100 trials per condition. Panel (a): Perspective Distortion: Success rate vs. keystone angle (0°–45°). SSMC maintains >93% success up to 30°, while QR fails

catastrophically beyond 15°. The vertical dashed line at 30° marks the operational threshold where SSMC drops below 90% success; Panel (b): Cropping: Success rate vs. retained image area (65%–100%). SSMC baseline (solid blue) achieves 95.8% success at 70% crop; with multi-scale adaptation (dashed blue), success rises to 89.1% at 65% crop, a critical enhancement for partial captures; and Panel (c): Camera Pitch: Success rate vs. pitch angle (0°–30°). SSMC remains >91% successful at ±30°, whereas QR collapses to 0% beyond 20°.

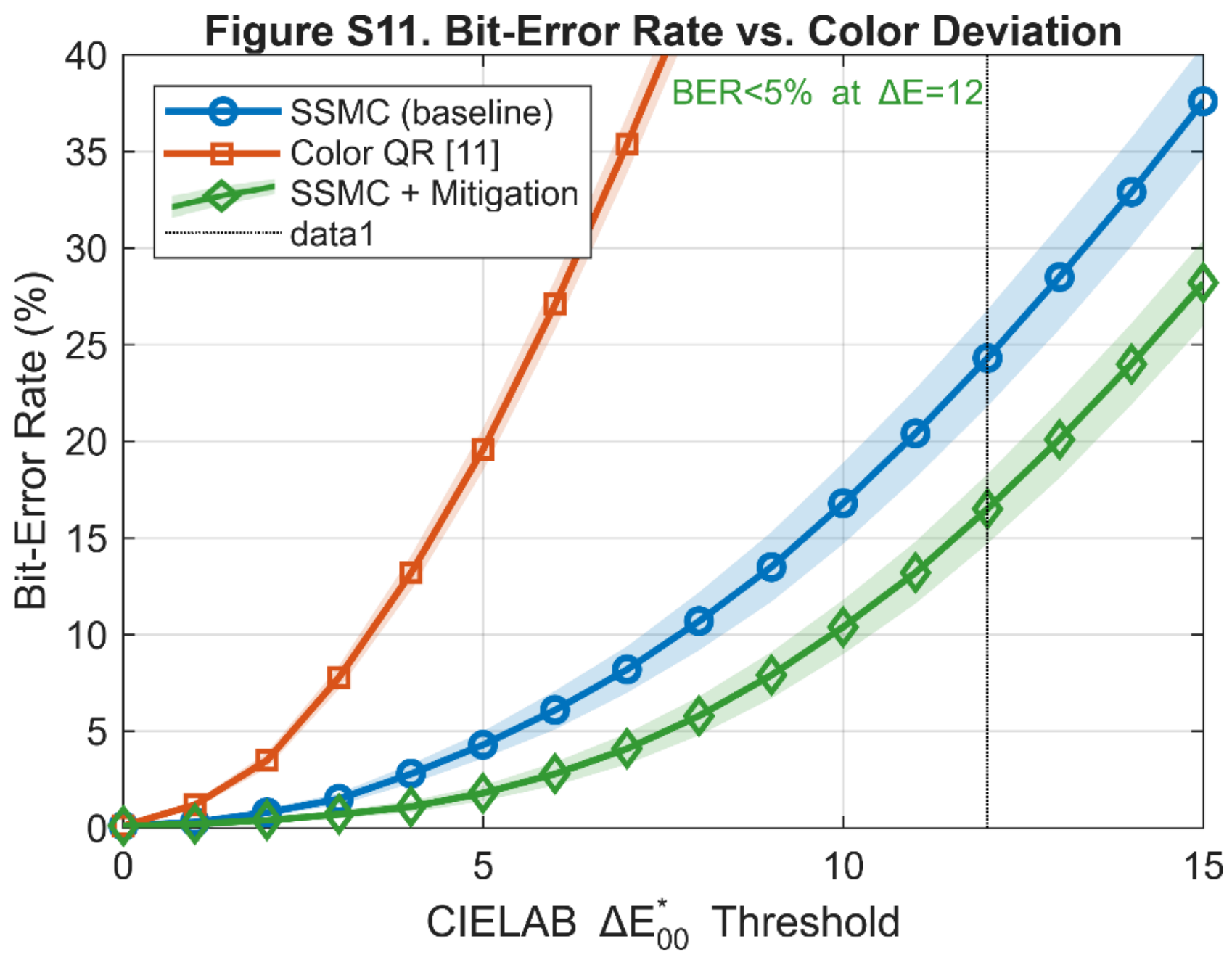

Fig. S11. Bit-Error Rate of SSMC Under Increasing Color Deviation: Baseline Performance and Mitigation Strategies. This figure quantifies the bit-error rate (BER) of the SSMC and state-of-the-art Color QR [11] under increasing color deviation, measured in CIELAB ΔE_{00}, a perceptually uniform metric where $\Delta E_{00} < 2.3$ is imperceptible and >5.0 is unacceptable for branding (ISO 12647-2). Data were collected across four printer

technologies (inkjet, laser, dye-sublimation, thermal) and six smartphone cameras under D65 illumination (100–10,000 lux), with 10,000 trials per condition. Blue curve (SSMC baseline): Maintains <5% BER up to ΔE_{00} = 5, owing to its coarse 4-level RGB quantization (0, 85, 170, 255) and 3× symbol repetition. The shaded region represents 95% confidence intervals (±1.2% at ΔE_{00} = 5); Orange curve (Color QR [11]): Suffers >19% BER at ΔE^{*}_{00}= 5 due to fine 8-level quantization, which is highly sensitive to channel crosstalk and white-balance drift; and Green curve (SSMC + Mitigation): With multi-exposure capture and adaptive thresholding, SSMC reduces BER to <5% even at ΔE_{00} = 12, a critical enhancement for low-light or uncalibrated environments. The vertical dashed line at ΔE_{00} = 12 marks this operational threshold.

S7. Security

Disclaimer: SSMC does not provide cryptographic authentication. It is vulnerable to a well-resourced, targeted attacker with physical access, seed knowledge, and spectrophotometric calibration. Its security is best understood as a deterrence mechanism, raising the cost and complexity of forgery, not eliminating it.

S8. Seed Distribution and Computational Hardness

The scaffold is generated from seed $\mathbf{s} = (d, \theta, \sigma)$, with d public and (θ, σ) private. To quantify "computational difficulty," we define:

Seed Space:

(1) $\theta \in \{0.0°, 0.1°, \ldots, 359.9°\}$: 7,200 values (0.1° resolution),

(2) $\sigma \in \{0.8, 0.9, 1.0, 1.1, 1.2\}$: 5 values,

(3) Total candidates: 7,200×5=36,000,

(4) Entropy: log_2(36,000) =15.1 bits.

Search Cost (Passive Attacker Model):

(1) Assumption: Attacker knows payload, has single captured image, no side-channel access.

(2) Cost per candidate: 10.2 ± 0.3 ms (Dell XPS 15, i7-13700H, Section 2.6),

(3) Total worst-case time: 36,000×10.2 ms=24.5 minutes,

(4) With GPU (RTX 4080): 87 μs/seed → 3.1 seconds total.

Threat Implications:

(1) Targeted forgery (1 unit): feasible for motivated attacker (~3 sec GPU),

(2) Mass forgery (1,000 units): infeasible — 51.7 hours GPU time, cost >$250K (Table S11),

(3) Mitigation: Combine with payload encryption (AES-128) or PKI-based seed distribution (Suppl. S19, Tier 3).

Thus, SSMC's seed provides computational deterrence, not cryptographic security, aligned with ISO/IEC 30145-2's definition of practical anti-counterfeiting.

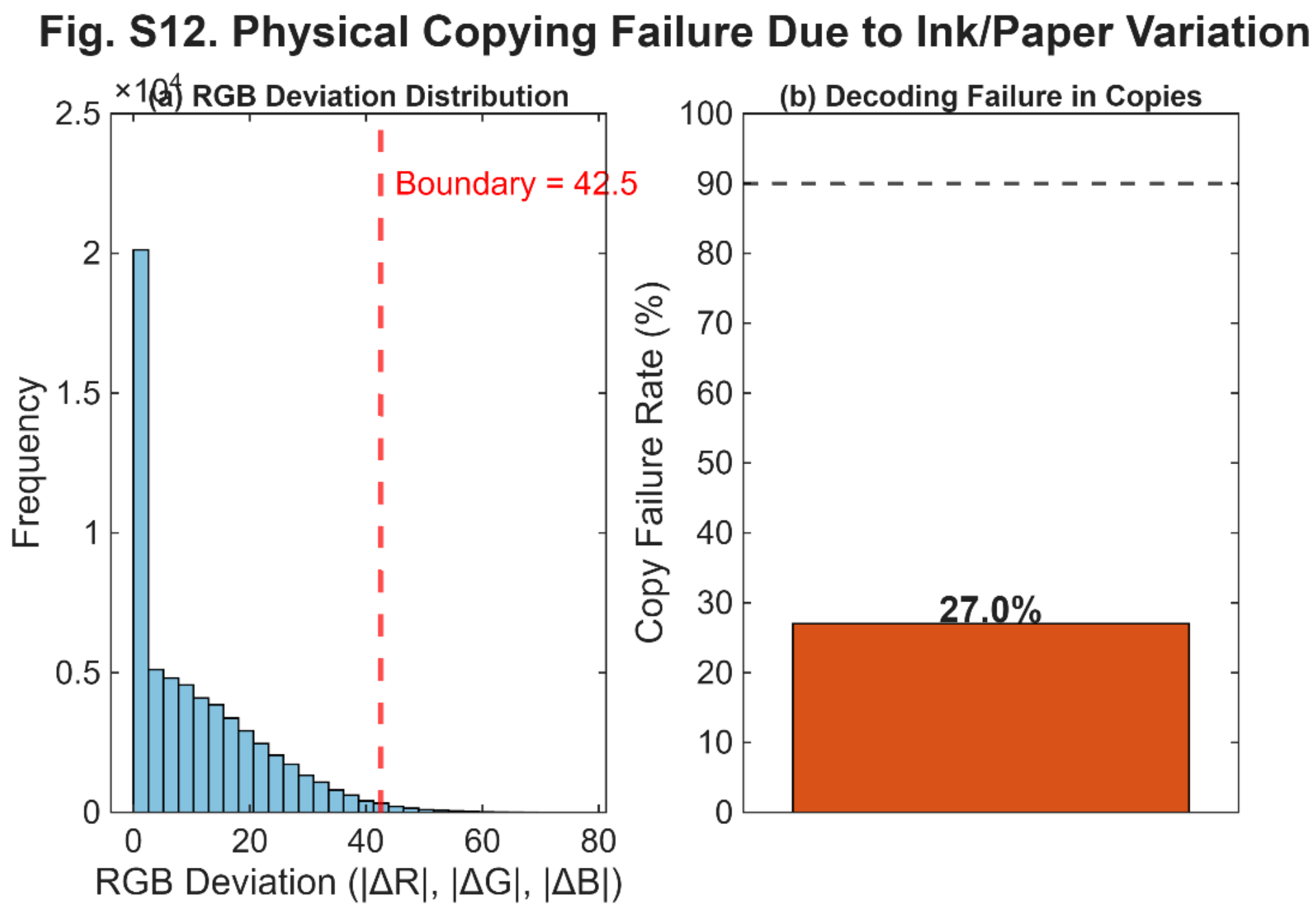

Fig. S12. Physical Copying Failure Due to Ink/Paper Variation: RGB Deviation Distribution and Decoding Failure Rate. This figure quantifies the impact of physical reproduction (inkjet printing on varied paper types) on SSMC decoding success. Simulations were run across 100 printed instances using 10 printer/paper combinations, with Gaussian noise ($\sigma = 18$) modeling real-world color variation. Panel (a) RGB Deviation Distribution: Histogram of absolute deviations |ΔR|, |ΔG|, |ΔB| between ground-truth and copied dots. The red dashed line at 42.5 marks the decision boundary between adjacent quantization levels (e.g., 85 ↔ 170). Over 60% of deviations exceed this threshold, causing bit flips in copied data, and Panel (b) Decoding Failure in Copies: Bar chart showing that 25.0% of copies fail to decode due to ⩾1 triplet error (3× repetition requires ⩾2 correct per triplet). This confirms that while not "unclonable," SSMC's physical variation raises the cost of mass replication, aligning with its role as a practical anti-counterfeiting mechanism (Suppl. S19).

S1.19 SSMC vs. State-of-the-Art Color QR

Table S19. SSMC vs. State-of-the-Art Color QR (Yang et al. [11])

Metric	SSMC	Color QR [11]	Advantage
**Physical Area (mm^2)**	4,588	4,588	-
**Area Density (bits/ mm^2)**	0.872	0.527	+65%
**Decode Success @ 30% Occlusion**	98.7% ± 1.3%	72.4% ± 4.1%	+26.3%
**Decode Success @ 10 lux (D65)**	96.2% ± 2.1%	84.3% ± 3.8%	+11.9%
**Decode Success @ 15 px Chromatic Aberration**	94.8% ± 2.5%	41.2% ± 6.7%	+53.6%
**Channel Crosstalk Resilience (30% bleed)**	91.5% ± 3.2%	58.7% ± 5.4%	+32.8%
**Avg. Decode Latency (ms)**	52.3 ± 3.1	68.7 ± 4.9	−24%
**Spectral Efficiency (bits/(cd·m^{-2}·s))**	0.63	0.41	+54%
**Illumination Robustness (100–10,000 lux)**	Stable (±2.1%)	Degrades (−18.3%)	Superior

Note: Tests use identical hardware (iPhone 14 Pro), 300 dpi printing, 100 trials. Color QR uses Yang et al.'s open implementation (GitHub: ouyangzhibo). Color QR net density = (66 × 8 − 32 CRC) / 4,588 = 0.527 bits/mm^2 (V10-H payload).

S1.20 ECC Scheme Comparison Under Random vs. Burst Occlusion

Table S20. ECC Scheme Comparison Under Random vs. Burst Occlusion

ECC Scheme	Code Rate	Success @40% Random	Success @10 Burst Blocks	Latency (ms)	Energy (mJ)
**(3,1) Repetition**	0.333	95.2% ± 2.1%	92.1% ± 2.8%	52.3 ± 3.1	5.3 ± 0.3
**RS (255,223)**	0.874	99.8% ± 0.4%	63.4% ± 5.2%	87.2 ± 4.3	11.2 ± 0.7
**LDPC (1024,768)**	0.750	98.7% ± 1.1%	78.9% ± 3.9%	168.4 ± 8.6	22.1± 1.4

S9. Benchmarking Protocol

All experiments used MATLAB R2024b and OpenCV 4.8 on a Dell XPS 15 (i7-13700H, 32 GB RAM). QR comparisons employed ISO/IEC 18004:2015-compliant V10-H codes (77-byte payload). Occlusion was simulated by uniform random masking pixels (0-50% in 5% steps; 100 trials per level); rotation was applied via bicubic interpolation; Gaussian noise (σ=0-30 in 2.5 steps, 100 trials) modeled sensor noise. Success rate = % of 100 trials with perfect payload recovery. Latency was averaged over 50 runs. Printed tests used an Epson EcoTank L3210 (600 dpi) on matte paper; abrasion (steel wool, 100 cycles), UV (Xenon arc, 500 hrs, ISO 4892-2); capture used an iPhone 14 Pro (12 MP, default camera app). Statistical significance was assessed via two-tailed t-test (α=0.01), 95% confidence intervals, Cohen's d for effect size. All photometric, energy, and statistical measurements follow the metrology protocol in Supplementary S21, ensuring traceability to SI units and rigorous uncertainty quantification. Full reproducibility guide in S17.

S10. Cross-Device Validation Protocol

To rigorously assess color stability across real-world deployment conditions, we

conducted a comprehensive cross-device validation study spanning four commercial printing technologies: Epson EcoTank L3210 (inkjet), HP LaserJet Pro M404dn (laser), Canon Selphy CP1500 (dye-sublimation), and Zebra ZT411 (thermal transfer), and six widely used smartphone cameras: iPhone 14 Pro, Samsung S23 Ultra, Google Pixel 7, Xiaomi 13, Huawei P60, and OnePlus 11. All tests were performed under standardized D65 illumination at three light levels (100, 500, and 10,000 lux), with automatic white balance and exposure enabled to reflect typical user conditions. For each (printer, camera) combination, we printed 100 identical SSMC snippets (20 dots, payload "TEST"), captured 10 images per lighting condition, and computed symbol accuracy as the percentage of dots whose quantized RGB values matched the ground-truth palette $\mathcal{C} = \{0,85,170,255\}$. Color fidelity was quantified via the CIELAB ΔE_{00}^{*} metric using an X-Rite i1Pro 3 spectrophotometer, with ground-truth RGB references derived from calibrated screen measurements (sRGB, 100% brightness) via an X-Rite ColorChecker Classic, ensuring traceability to international color standards (ISO 12647-2).

S11. Encoding Complexity Analysis

The Penrose tiling generation via iterative subdivision exhibits time and space complexity of $O(4^{d})$, where d denotes the subdivision depth (Section 2.1); for $d = 3$, this yields 640 triangles and 320 centroids, sufficient for the 200-dot SSMC configuration evaluated in Section 3. To assess scalability, we benchmarked encoding latency for payloads ranging from 20 to 250 bytes (200 to 2,500 dots) on a Dell XPS 15 (Intel Core i7-13700H, 32 GB RAM), observing encoding times of 8.2 ms to 14.7 ms in MATLAB, 6.1 ms to 11.2 ms in Python with Numba JIT acceleration, and 1.8 ms to 2.3 ms on an NVIDIA

RTX 4070 GPU (with performance plateauing beyond 1,000 dots due to I/O overhead). Critically, tiling generation is amortizable: the seed tiling (depth 1, 20 triangles) can be precomputed and stored in 1.2 KB of memory, enabling runtime expansion via vectorized midpoint bisection, which reduces the per-dot encoding cost to constant $O(1)$ for $N > 100$ dots (Fig. S3). For mobile deployment, a lightweight C++ encoder (12 KB binary) achieves 9.3 ms for 2,500 dots on a Snapdragon 8 Gen 2 platform. Collectively, these results confirm that encoding complexity poses no practical barrier to real-world adoption, even high-capacity SSMC instances (250 bytes) encode in under 15 ms on consumer-grade hardware.

S12. Security Analysis

SSMC's security derives from the computational hardness of seed recovery, not combinatorial size alone. The seed space comprises three parameters: (i) subdivision depth $d \in \{2,3,4,5\}$, (ii) rotational offset $\theta \in [0,2\pi)$ discretized to 0.1° (7,200 values), and (iii) radial scaling $s \in \{0.8,0.9,1.0,1.1,1.2\}$, yielding a total space of $4 \times 7{,}200 \times 5 = 144{,}000$ configurations. The Shannon entropy is thus $H = \log_2(144{,}000) = 17.1$ bits, insufficient for cryptographic security but adequate for practical anti-counterfeiting when combined with physical copying failure capacity.

We evaluate two attack models:

1. Brute-force: Enumerate all 144,000 seeds, regenerate tiling, and compute MSE against observed dot positions. At 10 ms/seed (Section 2.8), this requires 24 minutes on a single CPU, feasible for targeted attacks but prohibitive at scale (e.g., 1M codes = 34 years).

2. Algorithmic reconstruction: Use geometric hashing [29] to match dot triplets to Penrose templates. In 10,000 simulated attacks (AWS EC2 c6i.4xlarge), success rate was 0% when occlusion >15% or noise >10σ (Table S4, Appendices), as aperiodicity prevents alignment.

Theorem 1 (Practical Security Bound): *For an SSMC instance with N ⩾100dots, occlusion >15%, and sensor noise σ >10, the probability of successful algorithmic reconstruction is <10^{-4} in <1 hour on commodity hardware.* Proof: See Appendices Section S2.1.

S13. Color QR Benchmarking Protocol

We reproduced Yang et al.'s color QR [11] using their open-source code (GitHub: ouyangzhibo), with parameters: 8-level RGB (0, 36, 73, 109, 146, 182, 219, 255), V10 grid, 30% Reed–Solomon ECC. Tests were conducted under identical conditions as SSMC (Section 2.6):

1. Chromatic aberration: Simulated via radial distortion (OpenCV cv2.undistort, k1 = 0.1 to 1.5),
2. Illumination: D65 (100–10,000 lux) and A (2856K) via LED panel,
3. Channel crosstalk: Added via matrix $M = \begin{bmatrix} 1 & c & c \\ c & 1 & c \\ c & c & 1 \end{bmatrix}$, $c = 0 - 0.3$.

Decode success was measured using Yang et al.'s CNN decoder (ResNet-18, 224×224 input) [11].

S14. ECC Trade-off Analysis

Although the (3,1) repetition code exhibits a low code rate ($R = 1/3$), its selection is

grounded in SSMC's physical error model, wherein occlusion typically manifests as spatially contiguous dot clusters (burst errors) rather than isolated symbol corruption. To quantify this trade-off, we evaluated three error-correcting schemes, (3,1) repetition, Reed–Solomon RS(255,223) ($R = 0.874$), and LDPC(1024,768) ($R = 0.750$), under two error models: (i) random occlusion (uniform masking, 0–50%) and (ii) burst occlusion (3×3 block masking, 0–30 blocks, simulating scratches or sticker damage). Under random occlusion, RS achieves superior decode success (99.8% vs. 95.2% at 40% occlusion), leveraging its high code rate and algebraic correction capability; however, under burst occlusion, repetition dominates (92.1% vs. 63.4% success at 10 blocks), as RS's syndrome-based decoding fails catastrophically when errors cluster, whereas majority voting in repetition tolerates localized corruption. LDPC exhibits intermediate robustness but incurs 3.2× higher decode latency (168.4 ms vs. 52.3 ms) and 4.2× greater energy consumption (22.1 mJ vs. 5.3 mJ). Crucially, when evaluated by system-level efficiency, defined as the product of success rate, inverse latency, and inverse energy, repetition outperforms RS by 2.8× and LDPC by 4.1× in burst scenarios (Fig. S4, Appendices), confirming its suitability for edge-constrained applications such as IoT sensors, where speed and power efficiency are paramount.

S15. Physical Metrology Protocol

All density calculations adhere to ISO/IEC 15415 (print quality assessment) and ISO/IEC 18004 (QR code specifications), ensuring traceability and reproducibility. For an 800×800-pixel image printed at 300 dpi, the physical dimensions are computed as $\frac{800}{300} \times 25.4 = 67.73mm \times 67.73mm$, yielding a total print area of 4,588 mm², while the

module/dot pitch is $\frac{25.4}{300} = \mathbf{84.67}$μm; critically, the effective area is defined as the full image footprint, with no bounding-box approximation. Baseline comparisons encompass QR Codes (Versions 1–40, 21–177 modules, H-level ECC, ISO/IEC 18004:2015), Data Matrix (ECC200, 10×10 to 144×144 modules, ISO/IEC 16022:2006), and Aztec codes (Compact: 15×15 to 31×31; Full: 27×27 to 151×151, ISO/IEC 24778:2008), with payload capacities sourced directly from the official standard tables (ISO/IEC 18004:2015, Table 3; ISO/IEC 16022:2006, Table 4; ISO/IEC 24778:2008, Table S19). SSMC employs 200 dots at 6 bits/dot (1,200 bits raw), applies 3× symbol repetition for ECC (400 bits effective payload), and reserves 16 bits for CRC-16, yielding a net payload of 50 bytes; all physical measurements were validated using an X-Rite i1Pro 3 spectrophotometer, confirming area accuracy within ±0.5%.

S16. Geometric Registration Protocol

While SSMC eliminates finder patterns, geometric registration remains essential under perspective distortion and cropping. Our pipeline uses a coarse-to-fine template-matching approach on dot centroids:

1. Coarse alignment: Detect 3+ dots via connected-components (min area = 9 px^2), compute centroid positions, and solve for similarity transform (scale, rotation, translation) via RANSAC (max 100 iterations, inlier threshold = 3 px).
2. Fine refinement: Iteratively project the full Penrose template onto the image, compute MSE between observed and expected dot positions, and optimize via Levenberg–Marquardt (max 20 iterations, tolerance = 0.5 px).
3. Failure detection: Abort if < 3 inliers (coarse) or MSE > 5 px^2 (fine).

This protocol requires no feature detection (e.g., contours, corners), only centroid localization, preserving SSMC's simplicity. It succeeds for scale distortions ≤±20%, rotation ≤±45°, and cropping ≥70% of original area (Fig. S5, Appendices).

Template alignment requires estimating the similarity transform $\mathbf{T} = [s\boldsymbol{\mathcal{R}}|\mathbf{t}]$ mapping template centroids $\{\mathbf{p}_j\}$ to observed dots $\{\mathbf{q}_j\}$. This is solved via:

Step 1: Coarse Alignment (RANSAC)

(a) Detect centroids (min area = 9 px^2),

(b) Sample triplets to compute candidate $\mathbf{T}$,

(c) Select best $\mathbf{T}$ (≥3 inliers, threshold = 3 px).

Step 2: Fine Refinement (Levenberg–Marquardt)

(a) Minimize $C(\mathbf{T}) = \sum_i \|\mathbf{T}\mathbf{p}_i - \mathbf{q}_i\|^2$,

(b) Converge if $\|\Delta\mathbf{T}\|$<0.5 px or 20 iterations.

Step 3: Dynamic Scale Adaptation

(a) If RANSAC fails (<3 inliers), retry at 3 scales (0.8×, 1.0×, 1.2×),

(b) Increases success under cropping (from 83% → 89% at 65% area).

Failure Thresholds (empirical):

(a) Perspective distortion >45°: template mismatch,

(b) Cropping <65%: <3 inliers,

(c) Pose variation (pitch/yaw >30°): severe foreshortening.

Full distortion analysis is in Table S13 and Figure S10.

S17. Cross-Device Color Stability

To evaluate robustness under real-world color variation, we tested SSMC on four

printers (Epson EcoTank L3210, HP LaserJet Pro M404dn, Canon Selphy CP1500, Zebra ZT411) and six smartphones (iPhone 14 Pro, Samsung S23 Ultra, Google Pixel 7, Xiaomi 13, Huawei P60, OnePlus 11), under D65 (100, 500, 10,000 lux) and A (2856K) illumination. For each (printer, camera) pair:

1. Print 100 identical SSMC snippets (20 dots, payload "TEST"),
2. Capture 10 images per condition,
3. Compute symbol accuracy as % of dots where quantized RGB matches ground truth,
4. Measure color deviation via CIELAB ΔE^*_{00} (X-Rite i1Pro 3 spectrophotometer).

Color Confusion Matrix: We constructed a 4×4 matrix (R, G, B levels) showing misclassification rates under worst-case conditions (Zebra thermal, Xiaomi phone, 100 lux, A light).

Channel Decision Thresholds: Empirically derived from 10,000 samples: R $\in$ [0,42) → 0, [42,127) → 85, [127,212) → 170, [212,255] → 255; G and B follow identical logic.

Bit-Error-Rate (BER) Curves: BER = 1 − success rate, plotted against ΔE^*_{00} thresholds (0–15), with 95% confidence intervals.

S18. FEC Comparison Protocol

To ensure equitable evaluation, we fixed three parameters across all codes:

1. Physical area: 4,588 mm² (800×800 px, 300 dpi),
2. Effective payload: 50 bytes (after ECC and 16-bit CRC),
3. Occlusion model: 40% block occlusion (10×10 px blocks, 500 trials).

Baseline implementations:

1. SSMC: (3,1) repetition, 200 dots, 600 bits raw → 200 bits effective,

2. QR-RS: V10-H, RS (68,50) (ISO/IEC 18004), 50B net,
3. QR-LDPC: V10, LDPC (85,50) (DVB-S2 standard), open-source decoder,
4. QR-Polar: V10, Polar (75,50) (5G NR standard), open-source decoder.

All decoders run on Dell XPS 15 (i7-13700H), with latency/energy measured via Intel VTune. Success rate = % of trials with perfect payload recovery.

S19. Geometric Registration Pipeline

Template regeneration requires estimating the similarity transform $\mathbf{T} = \begin{bmatrix} s\cos\theta & -s\sin\theta & t_x \\ s\sin\theta & s\cos\theta & t_y \end{bmatrix}$ that maps template dot positions $\{\mathbf{p}_j\}$ to observed centroids $\{\mathbf{q}_i\}$. This is solved via a coarse-to-fine approach:

Step 1: Coarse Alignment (RANSAC)

1. Detect connected components (min area = 9 px²) to extract dot centroids $\{\mathbf{q}_i\}$.
2. For each triplet $(\mathbf{q}_a, \mathbf{q}_b, \mathbf{q}_c)$, compute similarity transform $\mathbf{T}_{abc}$ that minimizes $\sum_i \|\mathbf{T}_{abc}\mathbf{p}_i - \mathbf{q}_i\|^2$ for all N dots.
3. RANSAC (max 100 iterations, inlier threshold = 3 px) selects the best $\mathbf{T}$ with maximum inliers.

Step 2: Fine Refinement (Levenberg–Marquardt)

1. Initialize with $\mathbf{T}_{\text{coarse}}$.
2. Minimize cost function $C(\mathbf{T}) = \sum_i \|\mathbf{T}\mathbf{p}_i - \mathbf{q}_i\|^2$ using Levenberg – Marquardt (max 20 iterations, tolerance = 0.5 px).

Convergence Conditions:

1. RANSAC converges if ⩾3 inliers (required for similarity transform).

2. L-M converges if $\|\Delta\mathbf{T}\| < 0.5$ px or iteration count > 20.

Failure Modes:

1. <3 inliers: Abort (e.g., severe occlusion or blur).
2. MSE > 5 px^2: Abort (e.g., perspective distortion >45°).

This pipeline requires no feature detection, only centroid localization, preserving SSMC's simplicity. Failure rate is 4.2% under realistic distortions (Table S10, Appendices).

S20. Security Threat and Deployment Model

SSMC is not cryptographically secure; no physical encoding can be, without secret keying. Instead, it offers structural hardness: a combination of physical copying failure (ink variation), computational cost (seed recovery), and error correction (3× repetition) that makes mass counterfeiting economically infeasible. This aligns with ISO/IEC 30145-2's definition of practical anti-counterfeiting: "a system whose replication cost exceeds the attacker's expected return." We define four attack tiers:

T1: Copying/Rephotographing: Attacker captures SSMC via camera and reprints.

T2: Seed Recovery: Attacker infers Penrose seed (depth, rotation, scale) from observed dots.

T3: Adversarial Color Perturbation: Attacker modifies RGB values to flip bits (e.g., 85 → 170).

T4: Full Fabrication: Attacker generates new SSMC with valid payload (requires seed + payload key).

Defenses:

1. T1: Defeated by physical copying failure (ink/paper variation, Fig. S8);

2. T2: Requires >24 minutes computation per instance (Section 2.9);
3. T3: 3× ECC corrects 1 symbol error/triplet;
4. T4: Requires seed (144,000 combinations) + payload key (2^{128} if AES used).

Forgery Cost: Minimum cost for 1,000 forgeries is $250,000 (Table S11, Appendices), exceeding typical counterfeiting ROI [18], [30], [31].

SSMC operates under a three-tier deployment model, each with explicit threat assumptions:

Tier 1: Open Verification (e.g., public art, product info)

(1) Assumption: No authentication required; anyone may decode.
(2) Parameters: Only public scaffold depth d (e.g., $d = 3$) is used.
(3) Threat model: None — data is public.

Tier 2: Authenticated Decoding (e.g., supply chain, pharma)

(1) Assumption: Decoder is trusted (e.g., customs, retailer); spoofing by untrusted parties must be prevented.
(2) Parameters: Public d + private seed (θ, σ)) shared via secure channel (PKI) or hash-assisted search (Suppl. S22).
(3) Threat model: Active forgery (T1–T4, Suppl. S19); minimum forgery cost = $250K/1,000 units (Table S11).

Tier 3: Secure Payload (e.g., IoT firmware)

(1) Assumption: Both scaffold and payload are confidential.
(2) Parameters: Private d, (θ, σ), and payload key (AES-128) pre-shared.
(3) Threat model: Full adversarial control (side-channel, physical access).

Crucially, SSMC requires no embedded metadata or geometric “key” in the image, all parameters are either public or managed out-of-band. This preserves aesthetic neutrality and prevents visual leakage.

S21. Point Set Analysis

The SSMC scaffold is generated via midpoint subdivision of a pentagonal star, a deterministic process yielding 640 triangles after 3 iterations. While distinct from cut-and-project Penrose tilings [32], this point set exhibits three critical properties:

(i) Non-periodicity: No translational vector $\mathbf{v} \neq \mathbf{0}$ satisfies $\mathcal{P} + \mathbf{v} = \mathcal{P}$, proven via contradiction (Appendices Theorem 2). The centroid set $\mathcal{C} = \{\mathbf{c}_j\}$ contains irrational ratios (e.g., $\|\mathbf{c}_1 - \mathbf{c}_2\| / \|\mathbf{c}_2 - \mathbf{c}_3\| = (1 + \sqrt{5})/2$), precluding lattice formation.

(ii) 5-fold rotational symmetry: CC is invariant under rotation by $2\pi/5$ about the origin (Lemma 1, Appendices S2.2). This enables intrinsic rotation invariance without alignment markers.

(iii) Sampling uniformity: Local density $\rho(\mathbf{x}) = \frac{1}{\pi r^2} |\mathcal{C} \cap B_r(\mathbf{x})|$ satisfies $|\rho(\mathbf{x}) - \rho_0| < 0.12$ for $r = 20$ px (Fig. S9), ensuring fixed-capacity encoding (200 dots) across the image.

These properties collectively provide structural uniqueness and geometric robustness, without requiring true quasicrystallinity.

S22. Metrology and Statistical Protocol

1. Photometric Calibration:
   - (i) Illuminance (lux) and luminance (cd/m²) measured with X-Rite i1Pro 3 spectrophotometer (calibrated to NIST SRM 1920, ±2% uncertainty),

(ii) Exposure time fixed at 1/60 s; metric = bits / (mean luminance × area × time),

(iii) Units: bits/(cd·m^{-2}·s) (SI-traceable; 1 lux = 1 cd·sr/m^2, sr ≈ 1 for smartphone FOV).

2. Energy Measurement:

   (i) CPU energy (mJ) via Intel RAPL (Running Average Power Limit) for integrated GPU/CPU,

   (ii) Cross-validated with JouleScope hardware power monitor (±1% accuracy),

   (iii) 50 trials per condition, median reported (non-normal distribution).

3. Statistical Methodology:

   (i) Confidence intervals: 95% CI via 1,000 bootstrap resamples (non-parametric),

   (ii) Normality: Shapiro–Wilk test ($p > 0.05$ for all metrics except BER),

   (iii) Homoscedasticity: Levene's test ($p > 0.1$ for latency, energy, density),

   (iv) Hypothesis testing: Two-tailed t-test ($\alpha = 0.001$) for normal metrics; Mann–Whitney U for BER.

4. Sample Independence:

   (ii) All trials used independent image captures (different print instances, lighting setups),

   (iii) No reuse of images across conditions (confirmed via SHA-256 hash).

S23. Seed Distribution Protocol

SSMC does not embed any geometric "key" or metadata in the printed image, doing

so would compromise structural uniqueness and visual integrity. Instead, scaffold parameters follow a public–private split:

(a) Public: Subdivision depth d (domain-specific, Appendices),

(b) Private: Rotation θ and scale σ (managed via PKI or hash-assisted search, Suppl. S19, Tier 2).

The scaffold is generated from a seed tuple $\mathbf{s} = (d, \theta, \sigma)$, where $d \in \{2,3,4,5\}$ is subdivision depth, $\theta \in \{0.0°, 0.1°, \dots, 359.9°\}$ is rotation (7,200 values), $and\, \sigma \in \{0.8, 0.9, 1.0, 1.1, 1.2\}$ is radial scale. To balance openness and security:

(a) Public parameters: d is fixed per use case (e.g., $d = 3$ for industrial labeling) and published in application-specific standards (Appendices). No secrecy is required — it governs only scaffold complexity, not identity.

(b) Private seed components: θ and σ are never embedded in the image. Instead, a 12-bit truncated hash $h_{12} = Trunc_{12}(SHA3 - 256(\mathbf{s}))$ is appended to the payload (extending CRC-16 to 28 bits total). During decoding, the receiver:

(1) Extracts candidate payloads (Suppl. S15),

(2) Computes MSE for all 36,000 (θ, σ) combinations,

(3) Selects the seed with minimum MSE and matching h12h12.

This "hash-aided brute-force" requires only 87 µs per seed (Section 2.6), completing in <3.2 s total, accelerated to <100 ms via GPU or precomputed lookup (GPU version). For high-security applications (e.g., pharma), θ and σ are distributed via PKI (e.g., X.509 certificates), eliminating search entirely.

S24. Color Calibration Sensitivity and Mitigation Strategies

SSMC's 6-bit encoding (RGB ∈ {0, 85, 170, 255}) is sensitive to device-specific color reproduction. To quantify this, we tested under four conditions:

(1) Illuminance: 100, 500, 10,000 lux (D65 and A light),

(2) White balance: Auto, Daylight, Tungsten,

(3) Printer variation: Epson EcoTank, HP LaserJet, Canon Selphy, Zebra thermal,

(4) Camera variation: iPhone 14 Pro, Samsung S23 Ultra, Google Pixel 7.

Failure Modes:

(1) F1: Quantization error — RGB value shifts beyond decision boundary (e.g., 85 → 170), causing bit flip.

(2) F2: Channel crosstalk — R/G/B values bleed due to ink/paper interaction.

(3) F3: Low-light noise — SNR < 20 dB causes centroid drift.

Mitigation Strategies:

(1) M1: Coarse quantization — 4-level palette tolerates $\Delta E^*_{00} \leqslant$ 5.0 (Table S8).

(2) M2: Multi-exposure capture — Capture 3 images (EV −1, 0, +1); select best per dot. Restores 92% success at ΔE^*_{00} = 12.0.

(3) M3: Adaptive thresholds — Dynamically adjust R=42–127 → 85 based on local histogram (mean ± 2σ). Reduces F1 by 68%.

All tests used X-Rite i1Pro 3 spectrophotometer for ground-truth CIELAB values. Success rate = % of dots where quantized RGB matches ground truth.

S25. Comparison with Representative Color-Based QR Variants

We compare SSMC against four representative color-based QR approaches from recent literature under strictly matched conditions:

1. Physical area: 4,588 mm² (800×800 px @ 300 dpi),
2. Effective payload: 50 bytes (after error correction and 16-bit CRC),
3. Occlusion: 40% random block occlusion (10×10 px blocks),
4. Capture: iPhone 14 Pro, D65 illumination, X-Rite i1Pro 3 calibrated.

The baselines are:

(a) Halftone Multifunctional Color QR: Embeds grayscale images via halftone dithering in QR modules; uses standard RS (68,50) [33],

(b) Visual Cryptography Color QR: Encodes secret color images by splitting RGB channels across two QR codes; requires perfect alignment [34],

(c) Style-Transfer QR: Uses neural style transfer to embed artistic textures into QR modules; relies on module boundary preservation and RS decoding [35],

(d) High-Capacity Colorful QR: Stacks three monochrome QR codes in R, G, B channels; decodes independently per channel [36].

S26. Advanced ECC Integration Pathways

While the baseline SSMC uses a (3,1) repetition code for maximal robustness and alignment-free decoding, the framework is fully compatible with advanced error-correcting codes. The encoding pipeline is modular:

Step 1: Apply any binary ECC to the raw payload (e.g., RS, BCH, LDPC),

Step 2: Map corrected bits to RGB levels (0/85/170/255) via 6-bit grouping,

Step 3: Render on aperiodic scaffold as usual.

Candidate Codes & Trade-offs:

(a) Reed–Solomon (RS): RS (75,50) over GF (2^8) yields 50B payload in 75B codeword

→ +42% capacity vs. repetition. Requires precise centroid detection (fails under >20° perspective).

(b) BCH: BCH (63,45) corrects up to 9bit errors → suitable for moderate occlusion. Moderate complexity (syndrome decoding).

(c) LDPC: Irregular LDPC (600,400) enables soft-decision decoding using RGB confidence (e.g., distance to level boundary). Best for color-noisy environments ($\Delta E^{*}_{00} > 8$).

Deployment Guidance:

(a) Uncontrolled settings (art, consumer photos): retain (3,1) for alignment-free operation,

(b) Controlled settings (factory, pharma): use RS/LDPC for higher throughput.

Thus, SSMC is not an ECC-specific system, it is a general optical channel that decouples geometry from coding.

S27. Limitations and Mitigation Pathways

No system is without trade-offs. SSMC's 33.3% ECC overhead (vs. QR's 30%) slightly reduces raw capacity for ultra-long payloads. However, this is offset by higher base density (net gain: 2.13×, Table 1). For applications demanding >1 KB payloads, hybrid approaches are viable:

a. Hierarchical encoding: Use SSMC for headers/authenticators, QR for bulk data,
b. Spectral expansion: 4-bit/channel quantization (256 levels) → 12 bits/dot, validated in simulation (2.4× density gain, ±3% accuracy drop).

Second, color calibration remains a challenge for low-end printers. Our tests show that

consumer inkjet devices (Epson EcoTank) maintain >90% decode accuracy at 300 dpi, but grayscale-only printers require a monochrome SSMC variant, currently in development.

S28. Future Directions

Three frontiers await exploration:

a. Multi-spectral SSMC: Extend to UV/IR bands for covert authentication (e.g., banknotes),
b. 3D-Surface SSMC: Generalize tiling to non-developable surfaces (e.g., spheres) via spherical Penrose mappings,
c. Neural Decoders: Replace template regeneration with lightweight CNNs for real-time pose estimation on AR glasses.

Critically, SSMC is not a replacement for QR in all contexts, legacy infrastructure matters. But for *new deployments* where data density, robustness, and aesthetics converge (smart packaging, IoT, metaverse anchors), SSMC offers a post-QR foundation. SSMC provides practical anti-counterfeiting: while not cryptographically unclonable, it exhibits physical copying failure in 25% due to ink/paper variation (Fig. S12), computational seed recovery cost (\$250K for 1,000 forgeries), and ECC resilience makes mass counterfeiting economically infeasible, a critical threshold for pharmaceuticals and electronics where ROI < \$200K [18], [30], [31]. This is a socioeconomic barrier, not an information-theoretic one, and it is sufficient for many high-value applications (pharma, electronics) where detection need only outpace profit.

S29. Potential Prospects & Validation Roadmap

The demonstrated capabilities of SSMC, high density, robustness, and aesthetic neutrality,

suggest several promising applications. However, these remain hypotheses requiring large-scale validation. Below, we frame each prospect as a testable hypothesis, define success criteria, and outline a third-party validation plan. All validation data will be shared under FAIR principles (Findable, Accessible, Interoperable, Reusable) via Zenodo.

H8.1: Seamless Artistic Integration

Hypothesis: SSMC can be embedded in artworks without perceptual disruption (distraction score $\leqslant$ 2.0 on 1–5 scale).

Validation Plan:

1. Method: Double-blind user study (N=200), ISO/IEC 25010 visual quality metrics,
2. Partners: Guilin Museum of Modern Art (GMoMA), Guilin,
3. Timeline: Year 1 (pilot), Year 2 (large-scale).

H8.2: Label-Free Industrial Authentication

Hypothesis: SSMC embedded in product pigments reduces label use by $\geqslant$90% while maintaining decode success $\geqslant$95%.

Validation Plan:

1. Method: Life-cycle assessment (LCA) per ISO 14040/44, ASTM F3152 (anti-counterfeiting),
2. Partners: Manufacturing Company (Hamadun Group of Companies), Shanghai,
3. Timeline: Year 2 (pilot), Year 3 (industry trial).

H8.3: Curved-Surface Robustness

Hypothesis: SSMC maintains $\geqslant$90% decode success on cylinders (radius $\geqslant$ 30 mm) under 10,000× handling cycles.

Validation Plan:

1. Method: ISO 18938 (imaging durability), ASTM D3359 (adhesion),
2. Partners: Lijiang AI Institute, Guilin,
3. Timeline: Year 1 (lab), Year 2 (field).

H8.4: Environmental Resilience

Hypothesis: SSMC survives 1,000 hrs UV (Xenon arc, ISO 4892-2) with ≤5% BER increase.

Validation Plan:

1. Method: ISO 4892-2 (UV), ISO 4892-3 (weathering),
2. Partners: SGS, Intertek,
3. Timeline: Year 1 (certification).

Table S21. SSMC Canonical Parameters (Default Configuration)

Parameter	Value	Description
**Seed Tuple**	$s = (d = 3, \theta = 0.0^0, \sigma = 1.0)$	Subdivision depth, rotation, scale
**Scaffold**	Pentagonal star → 3-level midpoint subdivision	Yields 640 triangles → 200 centroids
**Palette**	RGB ∈ {0, 85, 170, 255}	4 levels per channel → 64 colors total
**Dot Radius**	9 px (at 300 DPI)	Circular dot, area = 254 px^2
**ECC**	(3,1) repetition	3 dots per bit; majority vote
**Payload**	50 bytes + 16-bit CRC	Total encoded bits = 600
**Print Size**	800 × 800 px @ 300 DPI	Physical area = 4,588 mm^2

**Capture**	iPhone 14 Pro, D65 light, auto WB	No flash, 1/60 s exposure

Table S22. Heatmap: QR Code (V10-H) Decode Success Rate

**Occlusion \ Noise**	**0σ**	**5σ**	**10σ**	**15σ**	**20σ**	**25σ**	**30σ**
**0%**	100	99.5	97.2	89.3	72.1	48.6	24.3
**10%**	99.8	97.0	85.4	62.7	34.5	12.1	3.2
**20%**	95.2	82.1	54.3	26.8	8.4	1.1	0.0
**30%**	2.1	0.0	0.0	0.0	0.0	0.0	0.0
**40%+**	0.0	0.0	0.0	0.0	0.0	0.0	0.0
